# A Review of the Effects of Supplementary Cementitious Materials on the Autogenous Shrinkage of High-Performance Concrete

**DOI:** 10.3390/ma19173594

**Published:** 2026-08-24

**Authors:** Jianming Zhou, Peihua Zhong, Wulong Zhang, Ziyi Wang, Xinwen Zhou

**Affiliations:** 1 School of Material Science and Engineering, Chongqing Jiaotong University, Chongqing 400074, China; 632308040114@mails.cqjtu.edu.cn (J.Z.);; 2 Jiangsu Sobute New Materials Co., Ltd., Nanjing 211103, China; 3 State Key Laboratory of Engineering Materials for Major Infrastructure, Nanjing 211103, China

**Keywords:** high-performance concrete, autogenous shrinkage, supplementary cementitious materials, prediction models

## Abstract

Autogenous shrinkage is a key factor contributing to early-stage cracking in high-performance concrete (HPC), which significantly affects structural durability and service life. As core components of HPC, supplementary cementitious materials (SCMs) can significantly improve concrete workability, mechanical properties, and durability, as well as reduce the risk of shrinkage cracking in HPC, by regulating hydration kinetics, pore structure, and microstructural evolution. The primary objective of this review is to elucidate the differential mechanisms by which different active pozzolanic materials regulate the autogenous shrinkage of HPC. This paper elucidates the patterns and mechanisms by which typical SCMs in HPC (such as fly ash, slag, silica fume, limestone powder, and nano-silica) affect the autogenous shrinkage of HPC. It analyzes the influence of key factors—including the type of SCMs, dosage, particle characteristics, water-to-binder (w/b) ratio, and composite blending on the autogenous shrinkage of HPC. Research indicates that highly reactive SCMs (such as silica fume and nano-silica) accelerate the self-drying process and increase autogenous shrinkage, whereas low-reactivity SCMs (such as fly ash) suppress autogenous shrinkage through dilution effects and by prolonging the hydration cycle. The combined use of multiple SCMs can achieve synergistic control of autogenous shrinkage and mechanical properties. Furthermore, this paper reviews existing autogenous shrinkage prediction models that account for the influence of SCMs and outlines future research directions. At the same time, this review identifies the limitations that currently exist in the research: there is a lack of a unified quantitative theoretical framework for the synergistic effects of multicomponent admixtures. The applicability of prediction models under multi-field coupling of temperature, humidity, and corrosive media is limited. And there is insufficient experimental data on the long-term shrinkage behavior of new low-carbon admixtures such as rice husk ash and calcined clay, which requires further dedicated research.

## 1. Introduction

HPC, with its superior mechanical strength, durability, and workability, has been widely used in high-rise buildings, long-span bridges, and special engineering structures [1,2,3,4,5,6,7,8]. However, to exhibit superior performance during service, HPC typically employs a low w/b (usually 0.22–0.35) and a high binder content [9], which results in significant early-age autogenous shrinkage, leading to cracking and compromising structural strength and durability. Autogenous shrinkage is defined as the macroscopic volume shrinkage of cementitious materials caused by self-drying due to hydration under sealed, isothermal conditions [10,11]. For HPC with a w/b less than 0.42, the internal free water is insufficient to meet the requirements for complete hydration of cement particles, leading to a rapid decrease in internal relative humidity and the generation of capillary negative pressure, which ultimately triggers autogenous shrinkage [12,13]. Cracks caused by autogenous shrinkage reduce the impermeability of HPC. These microcracks serve as pathways for the rapid penetration of chloride ions and sulfates, triggering a degradation process in which physical shrinkage cracking and chemical expansion damage are coupled, thereby significantly shortening the service life of concrete members [14,15]. Chloride ions destroy the passivation film on the surface of reinforcing bars and induce corrosion-induced expansion, while sulfates react with cement hydration products to form expansive compounds such as ettringite and gypsum, leading to secondary internal cracking and creating a continuously worsening cycle of deterioration. Conditions of high chloride ion corrosion are concentrated in marine industrial structures such as offshore wind turbine foundations, cross-sea bridges, port terminals, and desalination plants, which are subjected to long-term seawater immersion and alternating wet and dry salt fog cycles. Facilities such as brine storage tanks and salt mine tunnels within chlor-alkali and salt production plants are in prolonged contact with saturated chloride-containing waste liquids, and road and bridge surfaces in cold regions where de-icing salt is spread over extended periods also experience continuous accumulation of chloride ions [16]. High-sulfate corrosion environments are commonly found in wet-process metallurgy pickling workshops and tailings ponds that store high-sulfur mine wastewater, in coal chemical and phosphate fertilizer production facilities that generate large amounts of sulfur-containing byproducts and wastewater, in underground municipal pipeline networks and mine tunnels that are in prolonged contact with high-sulfur groundwater, as well as in various industrial plants constructed on saline–alkali soil foundations where pore water continuously accumulates sulfate. In all the above harsh industrial service environments, microcracks caused by early-age autogenous shrinkage of concrete significantly accelerate the chemical corrosion process [17]. The appropriate use of admixtures to suppress autogenous shrinkage and reduce the formation of microcracks is a key technical approach to enhancing the long-term durability of HPC in harsh industrial environments [18,19].

As an important component of HPC, SCMs play a key role in reducing clinker consumption, lowering carbon emissions, and enhancing overall performance [20,21,22,23,24,25,26,27,28]. Common SCMs include industrial by-products (fly ash, slag, silica fume) [29,30,31,32,33], natural minerals (metakaolin, calcined clay) [34,35,36,37,38], agricultural waste (rice husk ash) [39,40,41,42,43,44,45,46] and nanomaterials (nano-silica) [47,48,49,50], etc. These materials can regulate the hydration process and microstructural evolution of cementitious materials through physical effects (filling, dilution) and chemical effects (pozzolanic reaction, hydration synergy), thereby significantly influencing the autogenous shrinkage behavior of HPC. Studies have shown that incorporating supplementary cementitious materials (SCMs) is currently the most practical and viable core strategy for reducing the autogenous shrinkage of cementitious materials. However, the effectiveness of this approach is significantly influenced by the type, dosage, fineness, and water-to-binder (w/b) ratio of the SCMs [51,52]. This paper selects fly ash, slag, silica fume, limestone powder, and nano-silica as the primary subjects of analysis. These materials are widely used in engineering applications, and there is a substantial body of experimental data on their autogenous shrinkage. Furthermore, they cover low, medium, and high levels of reactivity, facilitating a comparative analysis of how reactivity affects autogenous shrinkage. Materials such as kaolinite, calcined clay, and rice husk ash also hold research value. Nevertheless, systematic experimental data on their autogenous shrinkage is relatively limited, and the performance of some of these materials is subject to significant fluctuations depending on the source of raw materials. Therefore, this paper does not focus on them in detail but only briefly discusses them in Section 4.6.

**Figure 1:**
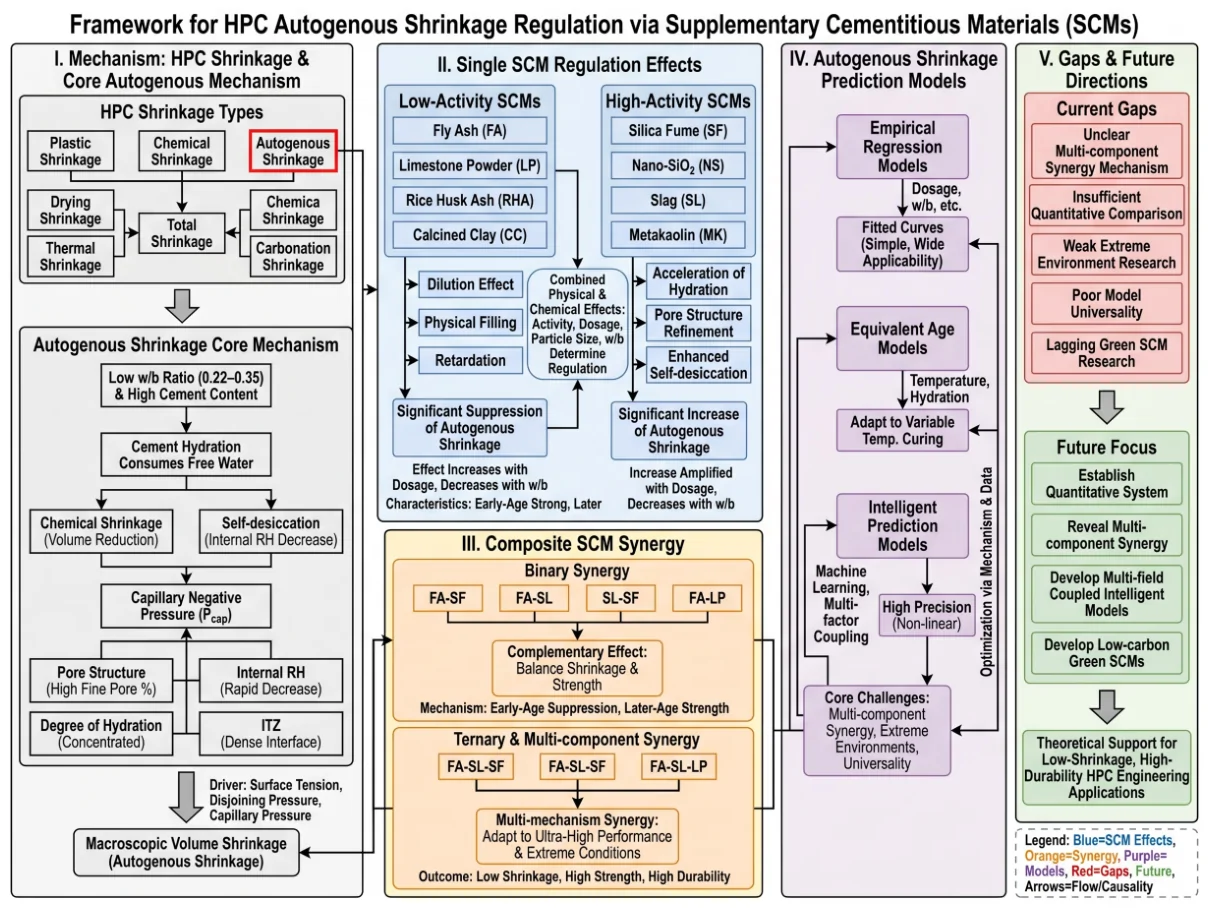
Schematic diagram of the system framework.

Currently, extensive research has been conducted on the effect of SCMs on the autogenous shrinkage of HPC. The inhibitory or enhancing effects of different types of admixtures on autogenous shrinkage have been widely confirmed, and composite admixture technology is gradually being applied to achieve synergistic optimization of shrinkage and mechanical properties. However, existing research still has significant shortcomings: (1) Quantitative comparisons and mechanistic analyses of different admixtures are not systematic enough. (2) Composite admixture formulations have primarily focused on binary systems, and the synergistic mechanisms of multi-component mixtures, optimal mix proportions, and universal design methods remain unclear. (3) Existing autogenous shrinkage prediction models largely rely on empirical fitting, with insufficient consideration of multi-factor coupling, resulting in limited prediction accuracy and scope of application. (4) The shrinkage regulation mechanisms of new green admixtures and shrinkage control technologies under extreme environmental conditions still need to be further refined. To address the aforementioned research gaps, this review classifies different admixtures according to their activity levels, systematically compares the differences in autogenous shrinkage control under single-admixture and multi-admixture conditions, and provides a cross-sectional analysis of various autogenous shrinkage prediction models that incorporate admixture parameters. It also identifies the strengths and limitations of each model and summarizes and compares conflicting experimental results found in some literature. The overall framework of this paper is shown in Figure 1, which visually illustrates the logical relationships and main research content of each chapter in this review.

To conduct this literature review, searches were performed in academic databases commonly used in domestic and international research (such as Scopus, ScienceDirect, and Google Scholar). In addition, the China National Knowledge Infrastructure (CNKI) and Wanfang databases were utilized. Search terms included combinations of the names of various admixtures with terms such as “high-performance concrete,” “types of shrinkage,” “effects on autogenous shrinkage,” and “autogenous shrinkage prediction models.” The search period was primarily limited to 2010–2026, with a focus on incorporating basic research and cutting-edge findings in this field from the past sixteen years. However, a small number of classic theories and landmark research results that laid the foundation for the field were retained and cited regardless of the time constraints. The literature was collected and organized using the reference management software Zotero (Version 9.0.6), and conference abstracts, popular science reports, and duplicate entries were first excluded. Second, priority was given to experimental studies, mechanistic analyses, and review articles published in SCI-indexed or core journals. Finally, in alignment with the research theme, high-quality literature focusing on the modification mechanisms of admixtures, the evolution patterns of autogenous shrinkage, and the construction of prediction models was selected to ensure the systematic, accurate, and cutting-edge nature of this review.

## 2. Types of Shrinkage in HPC

Concrete shrinkage refers to the phenomenon of volume reduction that occurs during the setting, hardening, and service life of concrete in the absence of external loads, such as that caused by water migration, chemical reactions, and temperature changes. It is an inherent non-load-induced deformation characteristic of cementitious materials. The mix design of HPC (characterized by a low w/b, high binder content, and a substantial proportion of mineral admixtures) results in shrinkage behavior that differs markedly from that of ordinary concrete. Specifically, HPC shrinkage is distinguished by its early initiation, greater magnitude, rapid progression, and increased cracking propensity. According to their origin mechanisms, occurrence timing, and driving factors, the shrinkage of HPC can be classified into six major categories: plastic shrinkage, chemical shrinkage (CS), autogenous shrinkage, drying shrinkage, temperature shrinkage, and carbonation shrinkage [12]. These different types of shrinkage overlap and interact over time, jointly contributing to the overall shrinkage deformation of HPC. Autogenous shrinkage represents the dominant factor governing early-stage volume changes in HPC [53], whereas drying shrinkage and thermal shrinkage critically influence long-term volume stability. SCMs have been shown to substantially regulate all forms of shrinkage.

### 2.1. Plastic Shrinkage

Plastic shrinkage occurs during the plastic stage between concrete placement and final setting, typically concentrated within 3 to 12 h after mixing. This is attributed to the surface water evaporation rate substantially exceeding the rate at which internal water can migrate and replenish the lost moisture. Due to its high paste content, high viscosity, and poor bleeding capacity, coupled with the water-adsorption effect of mineral admixture particles, HPC has a significantly higher risk of plastic shrinkage than ordinary concrete. Plastic cracks are more likely to form under high-temperature, high-wind, and low-humidity conditions [54].

The mechanism involves capillary water loss within the fresh paste, creating negative pressure that triggers overall volumetric settlement and shrinkage. Due to uneven support from aggregates, irregular cracks form on the surface when shrinkage stress exceeds tensile strength, severely compromising the concrete’s appearance and integrity. Studies have shown that the lower the water-to-binder ratio and the higher the total binder content in HPC, the more pronounced the plastic shrinkage. The addition of admixtures such as fly ash and limestone powder can improve the water retention of the paste through the ball-and-pin effect, thereby inhibiting the development of plastic shrinkage to a certain extent [55].

### 2.2. Chemical Shrinkage

**Figure 2:**
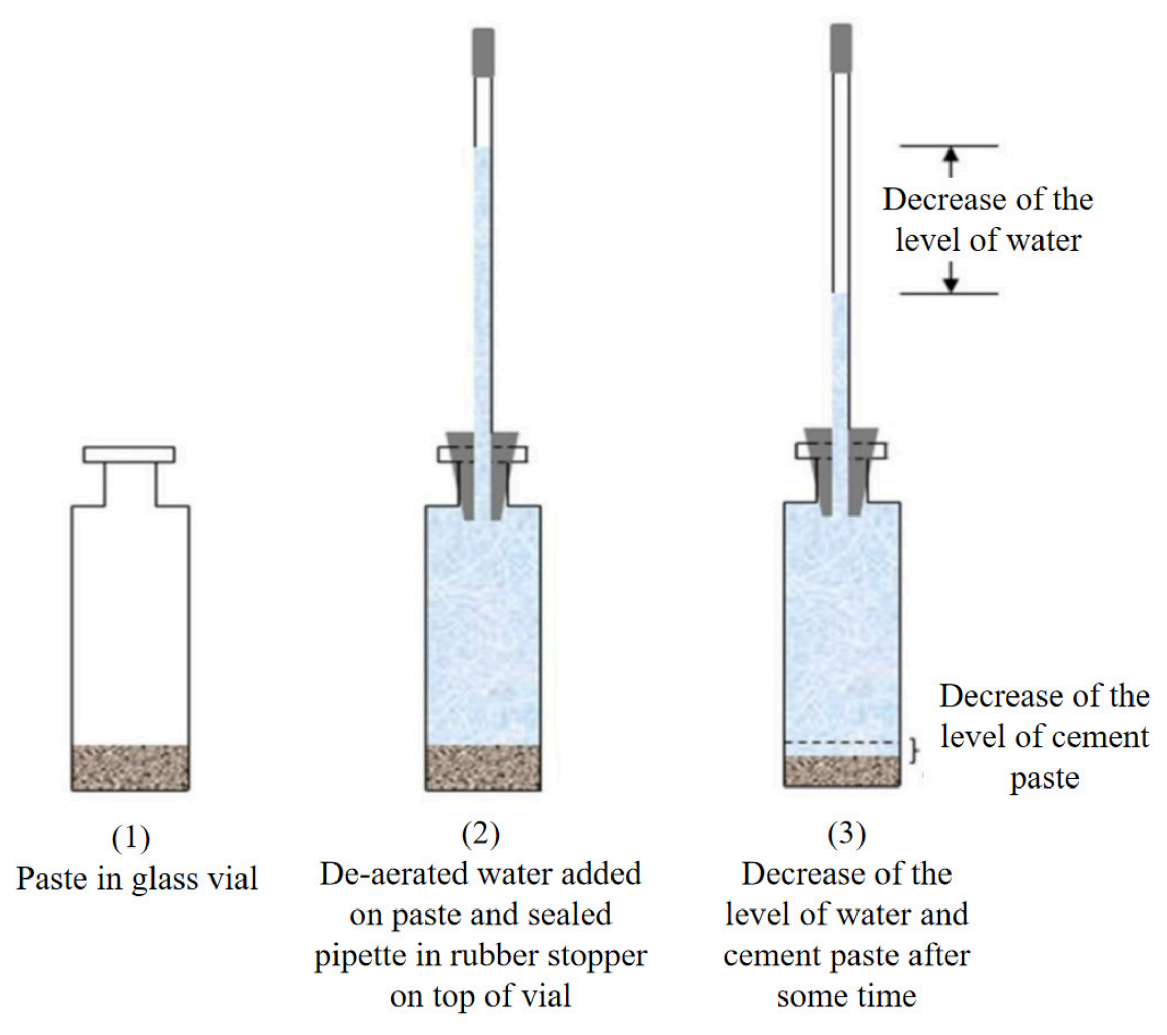
Schematic diagram of the chemical shrinkage testing method [59].

Chemical shrinkage (CS) is an inherent property of the hydration reaction of cementitious materials. It refers to the irreversible decrease in the absolute volume of the reaction system during the process in which cement and active admixtures react with water to form hydration products such as C-S-H gel, Ca(OH)_2_, and ettringite [56]. During cement hydration, the absolute volume of the reactants is greater than the total volume of the hydration products. This volume difference is known as CS. Kheir et al. [57] employed the ASTM C1608 [58] dilatometer method to conduct CS tests, as shown in Figure 2. Figure 2 provides a schematic illustration of the test setup, wherein a glass sample bottle (filled with cement paste and degassed water) is sealed by a rubber stopper fitted with a pipette, establishing a closed test system. The specimen is placed in a constant-temperature water bath for curing to prevent interference from water evaporation and temperature fluctuations. The decrease in hydration volume of the paste drives continuous water consumption, manifested visually as a persistent decline in the water level within the pipette, thus permitting the quantitative determination of CS [59]. Studies show that CS increases rapidly during the early stages of hydration and slows down in the later stages. For systems with a low w/b (≤0.2), high-performance plasticizer must be added and the mixture thoroughly vibrated while simultaneously controlling the paste thickness to <3 mm to ensure testing accuracy. CS exhibits an increasing trend as the w/b rises. At low w/b, the CS of CEM III (slag cement) is slightly higher than that of CEM I (Portland cement), while the opposite is true at high w/b. Low-reactivity fillers can reduce CS, and this inhibitory effect is further enhanced when multiple fillers are blended.

The CS of ordinary Portland cement is positively correlated with the degree of hydration. The more complete the hydration reaction, the greater the cumulative CS. For HPC systems, the high content of cementitious materials results in a total cumulative CS far higher than that of ordinary concrete. While the low w/b design leads to rapid accumulation of hydration products. The system lacks sufficient free water and hydration products to fill the volume void caused by CS, thereby forming initial internal voids. At the same time, HPC undergoes intense early-stage hydration with a rapid rate of CS, exhibiting a rapid increase in the first 7 days of hydration, which creates the necessary conditions for the subsequent occurrence of the self-drying effect and the formation of capillary negative pressure.

### 2.3. Drying Shrinkage

Drying shrinkage is a type of long-term shrinkage that occurs when concrete, after being removed from standard curing, loses moisture from its capillary and gel pores in an unsaturated environment over an extended period. This volume deformation can last for several years. The core driving forces are the internal humidity gradient and the difference between internal and ambient humidity. Moisture diffusion from the interior to the exterior leads to gel compaction and increased capillary negative pressure, ultimately resulting in macroscopic shrinkage. The difference in drying shrinkage between HPC and ordinary concrete essentially stems from the internal water phase distribution and pore structure determined by the water-to-cement (w/c) ratio [60]. After hardening, this type of concrete has a denser structure with finer and more tortuous moisture diffusion pathways. Consequently, its early-stage drying shrinkage rate is slower than that of ordinary concrete, but the total long-term drying shrinkage remains significant.

Due to its dense structure, fine pores, and low permeability, HPC exhibits slow water diffusion rates, resulting in an early-stage drying shrinkage rate lower than that of ordinary concrete. However, the long-term cumulative shrinkage remains significant. Compared to autogenic shrinkage, drying shrinkage is more significantly influenced by environmental humidity, wind speed, member dimensions, and exposed surface area. Drying shrinkage is more pronounced in thin-walled members and surface-layer concrete, and is one of the causes of late-stage cracking and deformation in HPC structures [61,62].

### 2.4. Autogenous Shrinkage

Autogenous shrinkage refers to the macroscopic volumetric shrinkage caused by self-drying in HPC under sealed, isothermal conditions with no internal–external water exchange, resulting from the continuous consumption of internal free water during hydration. Also known as autogenous shrinkage, it is the most representative form of shrinkage in low w/b HPC and a key factor leading to early cracking and reduced structural durability [12,13,14,15,16,17,18,19,20,21,22,23,24,25,26,27,28,29,30,31,32,33,34,35,36,37,38,39,40,41,42,43,44,45,46,47,48,49,50,51,52,53]. Unlike drying shrinkage, autogenous shrinkage originates from internal hydration water consumption rather than external evaporation, and is particularly significant when the w/b is <0.42. In HPC systems with typical w/b of 0.22–0.35, autogenous shrinkage can account for more than 50% of total early-stage shrinkage [63]. However, there remains some controversy in the existing literature regarding the effect of the w/b on autogenous shrinkage [13,64,65]. Most studies suggest that systems with low w/b (˂0.4) are prone to internal self-drying, which, in turn, leads to significant autogenous shrinkage. Chen et al. [66] conducted experiments under sealed curing conditions and found that within the w/b range of 0.25–0.35, the lower the w/b, the faster the relative humidity within the cementitious system decreased, and the higher the risk of early-age cracking in the test specimens. However, this empirical threshold shifts significantly with changes in the cementitious components. Li et al. [67] conducted tests on mortars blended with fly ash and nano-metakaolin. Their findings revealed that the sensitivity of autogenous shrinkage to changes in the w/b was significantly reduced after the addition of pozzolanic cementitious materials, even when the w/b was identical, different cementitious mixtures exhibited vastly different autogenous shrinkage deformations. Under ultra-low w/b conditions, non-monotonic behavior also occurs. The results of the pure mortar tests by Ge et al. [68] showed that when the w/b drops to around 0.18, the system suffers from a severe shortage of free water available for hydration, inhibiting the hydration process. Further reductions in the w/b do not result in any further increase in autogenous shrinkage. A synthesis of existing research findings indicates that there is no single critical w/b applicable to all HPC. The critical thresholds reported in the literature vary widely and are influenced by a combination of factors, including cement mineral composition, cement fineness, the type and dosage of SCMs, curing conditions, and the autogenous shrinkage testing method [13]. Therefore, when analyzing the influence of the w/b, it should not be treated as an isolated indicator but must be considered in conjunction with other material parameters.

**Figure 3:**
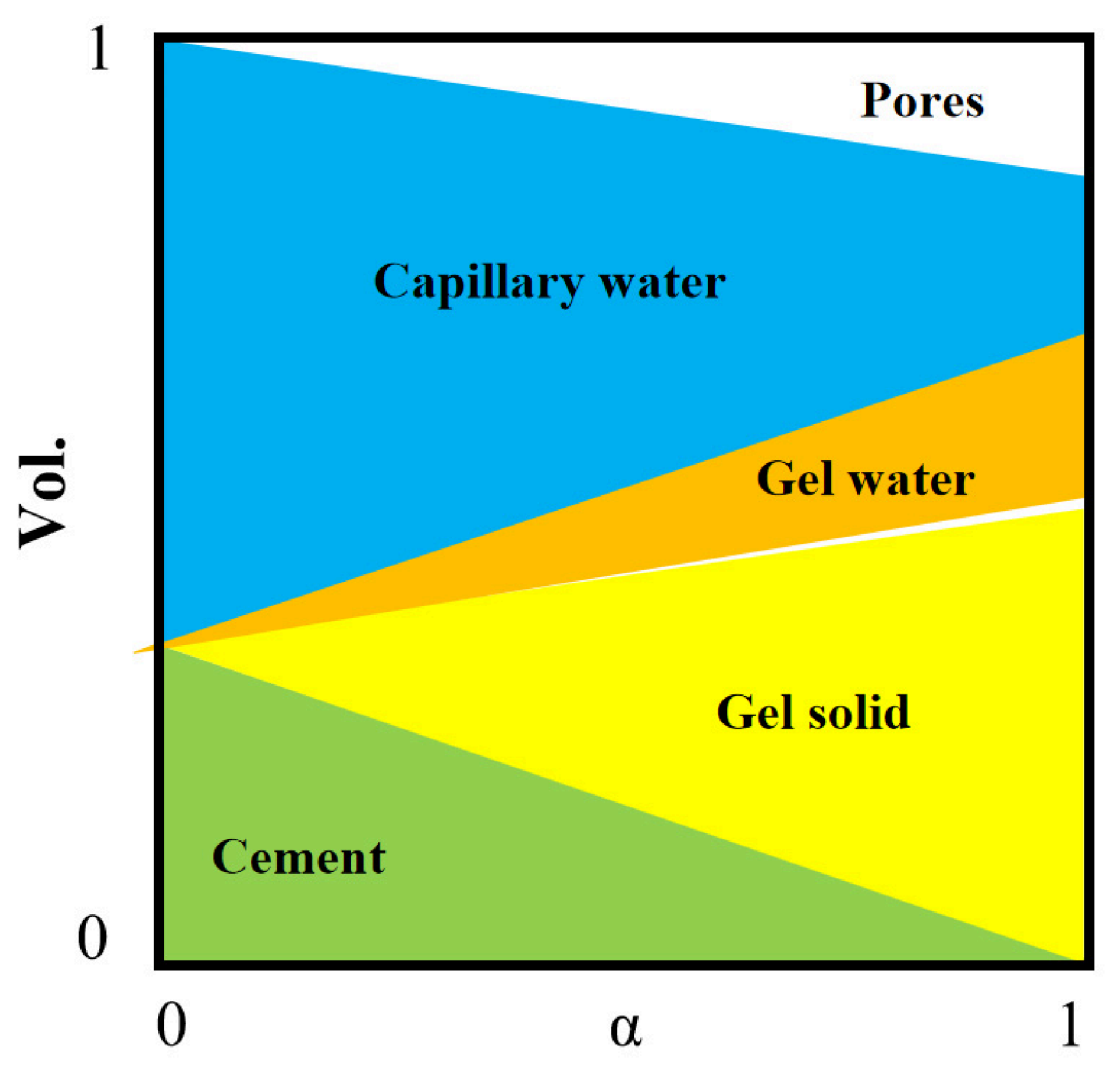
Volumetric phase distribution of cement paste as a function of the degree of hydration, α, at w/c = 0.6. The diagram applies to sealed hydration (=closed system), i.e., without exchange of water with the surroundings. Due to the high w/c ratio, full hydration (α = 1) of the cement can theoretically be obtained [69].

**Figure 4:**
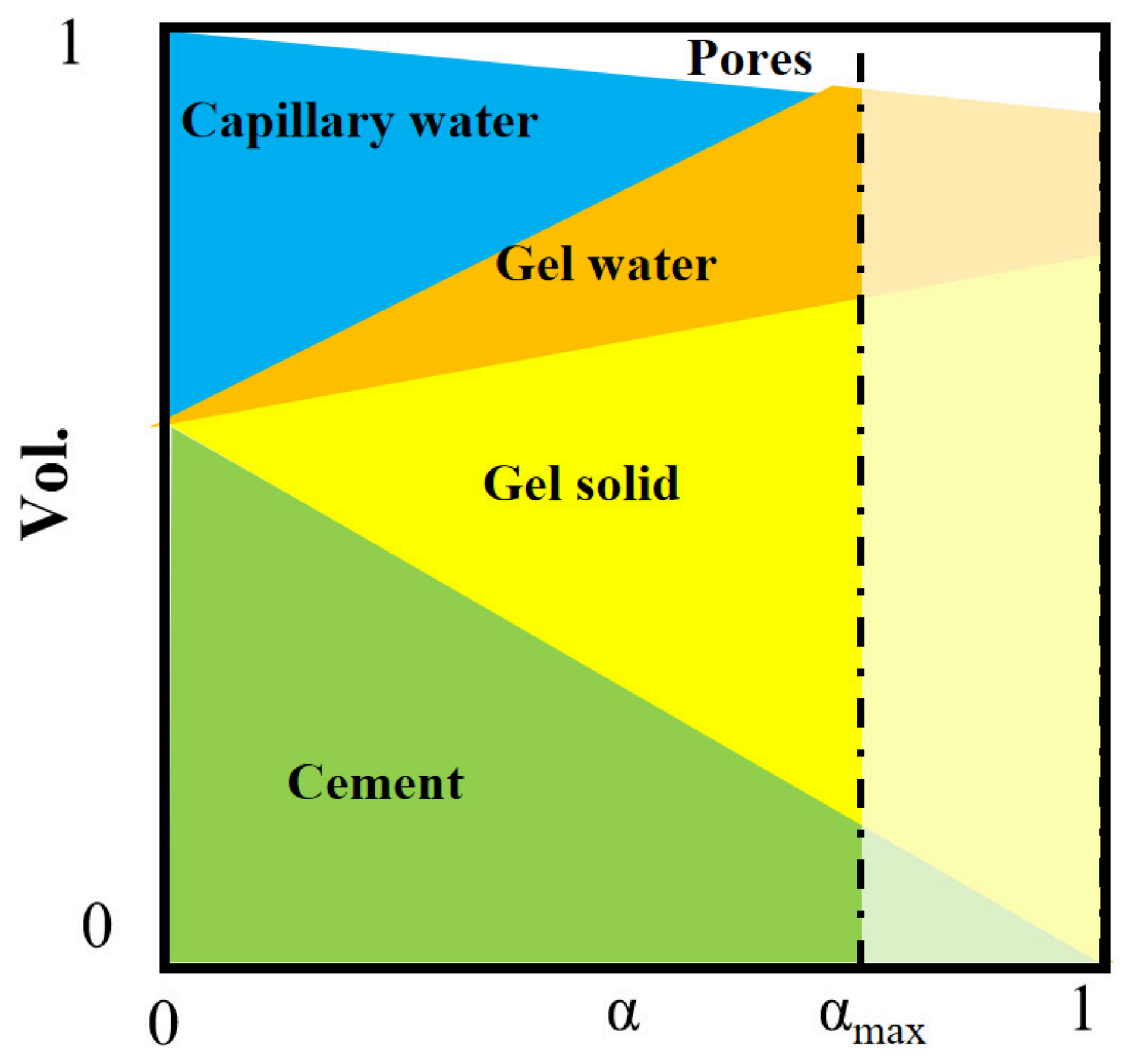
Volumetric phase distribution of cement paste as a function of the degree of hydration, α, at w/c = 0.3. The diagram applies to sealed hydration, i.e., a closed system. Due to the low w/c ratio, full hydration of the cement cannot be obtained [69].

Autogenous shrinkage results from the coupling of CS [13], the self-drying effect, and capillary pressure [12]. Cement hydration triggers CS, and this process reduces the total volume of the solid and liquid phases and consumes water within the pores, serving as the fundamental cause of self-drying. However, CS alone does not directly cause macroscopic shrinkage deformation. The self-drying effect reduces the saturation of the pore liquid phase, leading to an increase in capillary pressure, which, in turn, converts the shrinkage of the microscopic pore structure into macroscopic deformation of the material. It is worth noting that, under certain conditions, the self-drying effect’s reduction in IRH can also generate disjoining pressure. However, this disjoining pressure primarily acts on gel pores at the nanoscale and makes a limited overall contribution to autogenous shrinkage. This theory has received limited application in existing experimental and engineering research, and its operating conditions and applicable boundaries are discussed in detail in Section 3.2. In HPC systems with low w/b, capillary pressure plays a dominant role. HPC possesses a high proportion of fine capillaries and a dense structure, which results in higher capillary pressure. Its autogenous shrinkage can reach 2 to 4 times that of ordinary concrete, making it the primary factor inducing early cracking and reducing durability [13,53,54,55,56,57,59,60,61,62,63,64,65,66,67,68,69]. Based on Powers’ cement paste phase distribution model, this mechanism can be clearly elucidated [69]. As shown in Figure 3, in a sealed system with a high w/c ratio (w/c = 0.6), free water is abundant, and capillary water remains in excess throughout the hydration process. Cement can achieve complete hydration, and the internal relative humidity (IRH) is maintained at 100% for an extended period, with virtually no self-drying occurring. Drying shrinkage arises primarily from the evaporation of capillary water only when exposed to the environment. As shown in Figure 4, in a typical HPC system with a low w/c ratio (w/c = 0.3), there is a severe shortage of internal free water. Capillary water is rapidly depleted during the early stages of hydration, forcing the cement hydration process to cease, and significant self-drying occurs internally.

**Figure 5:**
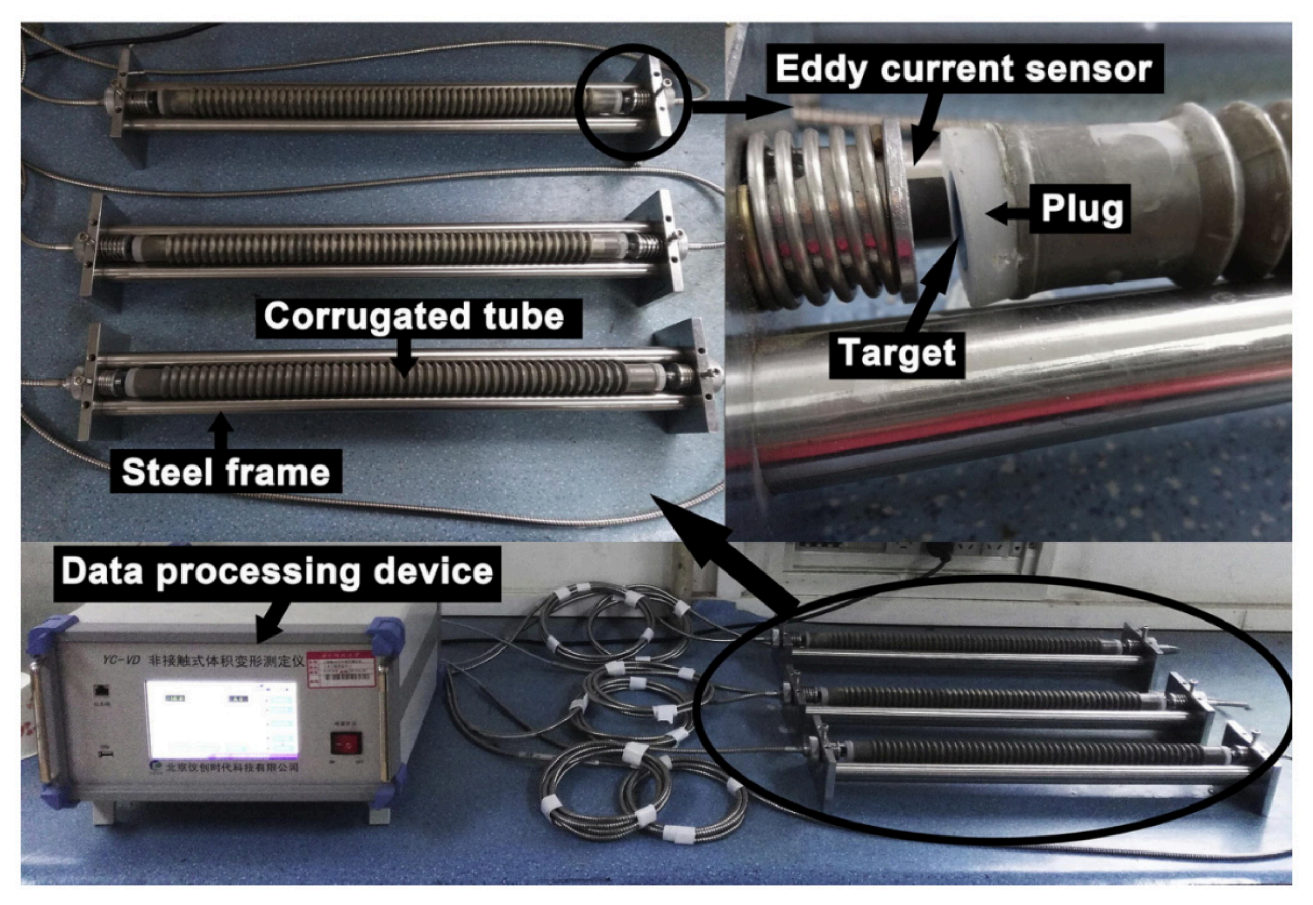
Experimental setup of non-contact corrugated tube method [75].

**Figure 6:**
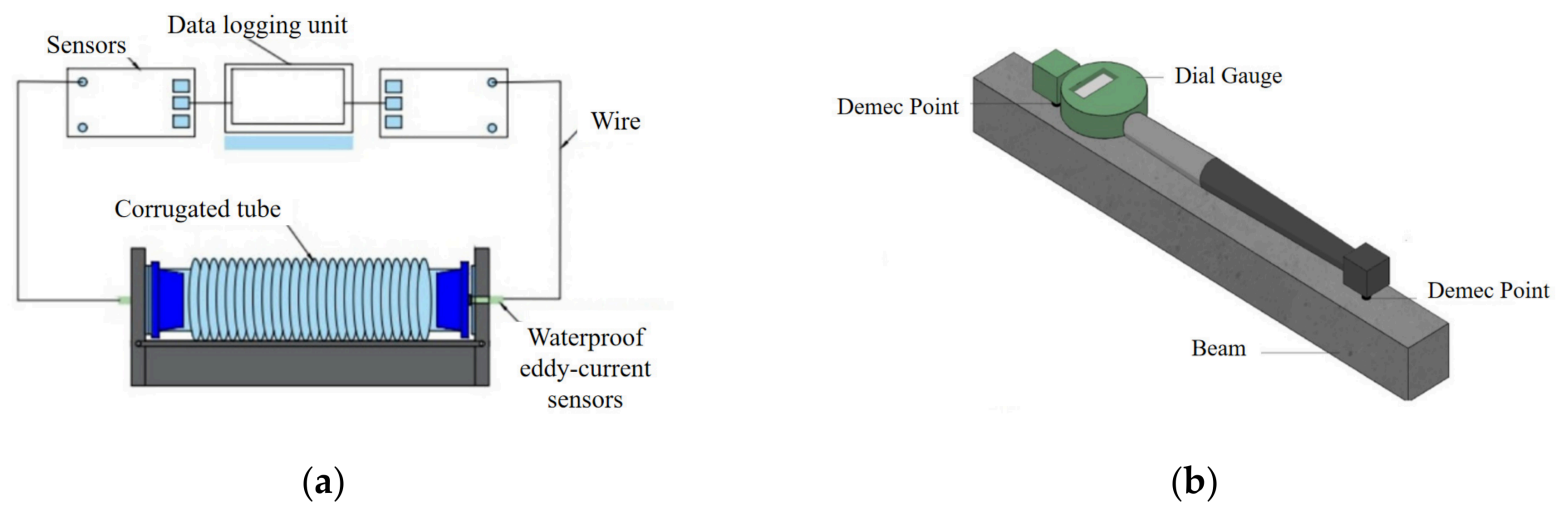
Autogenous Shrinkage measurements: (**a**) corrugated plastic tube (horizontal direction); (**b**) linear autogenous shrinkage measurement in hardened stage [13].

Currently, mainstream methods for testing autogenous shrinkage focus on sealed, constant-temperature, early-age continuous length measurement. They are primarily divided into three categories [70,71]: the volumetric method, the contact linear method, and the non-contact linear method. Although the volumetric method can be used for early-age testing, it is affected by the permeability of rubber bags, and is prone to puncture by aggregates. Non-contact linear methods, which utilize eddy current or laser sensors, allow for non-contact monitoring before initial setting, thereby effectively preventing relative slippage between the fresh paste and the sensors [72,73,74]. They offer advantages such as high accuracy and continuous, stable data, making them suitable for the precise characterization of early-age autogenous shrinkage in low water-to-cement ratio HPC. Figure 5 shows a non-contact bellows testing apparatus that uses an eddy current non-contact displacement sensor, combined with a polyethylene bellows specimen carrier and a frictionless steel support frame, to continuously monitor the early-age autogenous shrinkage deformation of cement-based materials in a temperature-controlled, sealed environment [75]. This apparatus utilizes the eddy current effect to detect changes in the displacement of a metal target, thereby avoiding contact friction between the sensor and the freshly mixed mortar and enabling high-precision testing of deformation during the ultra-early age. The ASTM C1698-09 [76] standard method uses a flexible bellows to eliminate constraints, enabling stable testing of the autogenous shrinkage of slurries and mortars. It is an internationally recognized standard testing method [77]. As shown in Figure 6, compared with the traditional concrete beam dial gauge method (Figure 6b), the linear autogenous shrinkage measurement device based on this standard primarily uses a flexible bellows as the specimen carrier. Its low-constraint characteristics release boundary restrictions, allowing cementitious materials to undergo free deformation during hydration while effectively reducing the interference of moisture loss on test results. A typical configuration uses a horizontal bellows paired with waterproof eddy-current sensors (Figure 6a), which enables continuous and stable acquisition of autogenous shrinkage strain and represents one of the most commonly used testing systems in research on the autogenous shrinkage behavior of HPC.

### 2.5. Temperature Shrinkage

Temperature shrinkage (cooling shrinkage) refers to the volumetric shrinkage that occurs during the cooling phase due to thermal expansion and contraction, following the exothermic heating of concrete caused by hydration [78], which is particularly pronounced in large-volume HPC structures. The high content of cementitious materials in HPC induces a rapid hydration exothermic rate and results in high peak temperatures. The internal temperature increases can reach 50–80 °C. During the cooling process, thermal deformation is constrained by the substrate and reinforcing bars, easily generating significant tensile stress. When the stress surpasses the tensile strength, thermal cracks develop. The study by Maruyama et al. [79] revealed that temperature history plays a key role in the development of autogenous shrinkage of cementitious materials with low w/b, based on monitoring of their early-stage shrinkage behavior. Early-stage low-temperature environments significantly accelerate the rate of shrinkage, while late-stage high temperatures further increase the final amount of shrinkage, resulting in a distinct two-stage temperature dependence of shrinkage. The addition of mineral admixtures such as fly ash and slag can reduce the peak hydration heat, delay the heat release rate, effectively mitigate the combined effect of thermal shrinkage, and improve crack resistance.

### 2.6. Carbonation Shrinkage

**Figure 7:**
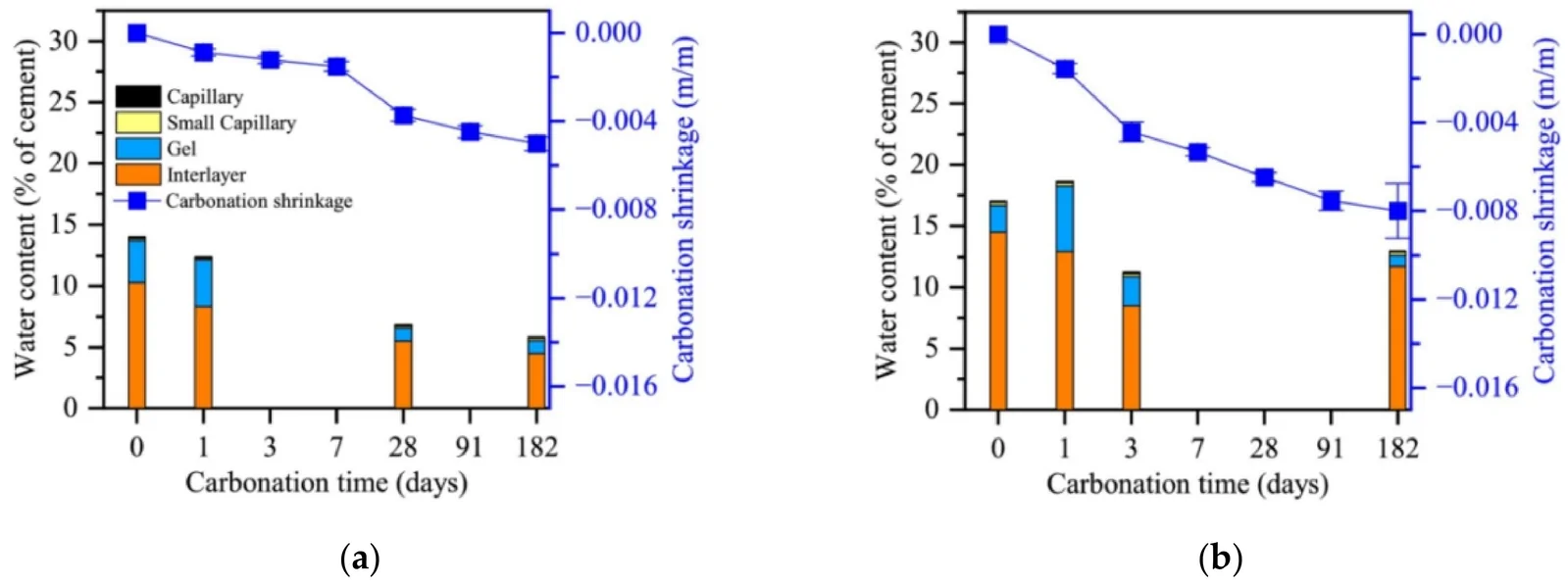

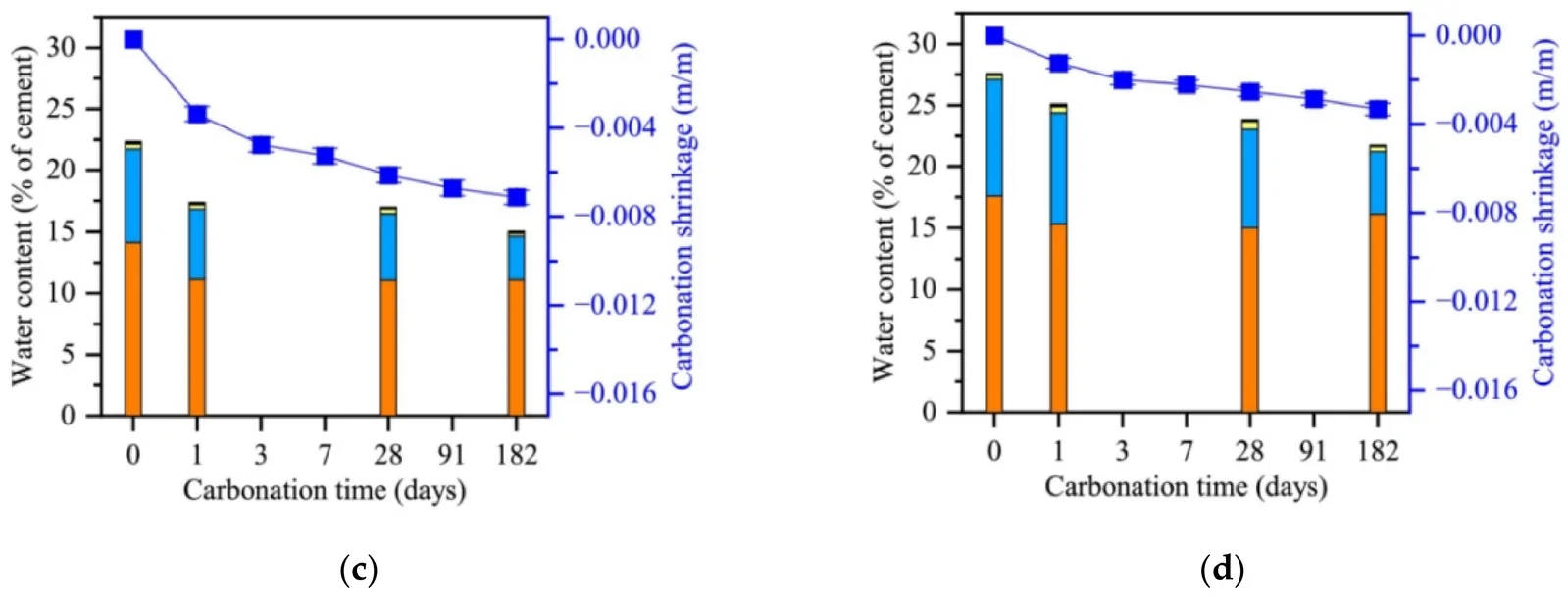
Variation in the water contents in interlayer space, gel pore, small capillary pore and large capillary pore, and carbonation shrinkage over carbonation time [81]: (**a**) 40%RH; (**b**) 52%RH; (**c**) 70%RH; (**d**) 85%RH.

Carbonation shrinkage is a phenomenon in which, after concrete has hardened, hydration products such as Ca(OH)_2_ and C-S-H gel react with CO_2_ in the air through carbonation, forming products such as CaCO_3_ and releasing water, thereby causing a reduction in volume [80]. As shown in Figure 7, during the carbonation process, interlayer water and gel water in the C-S-H structure continue to be lost. The migration of water, in synergy with the decalcification of hydration products, causes irreversible volumetric shrinkage in the slurry. Carbonation shrinkage strain increases continuously with carbonation age, with the fastest shrinkage rate and greatest deformation occurring under moderate humidity conditions of 52% and 70%. Under low-humidity conditions (40%), CO_2_ diffusion is impeded, while under high-humidity conditions (85%), the pores are filled with water, in both cases, the carbonation reaction is inhibited, resulting in significantly lower carbonation shrinkage [81]. The shrinkage deformation caused by the carbonation reaction persists throughout the entire service life, reduces concrete alkalinity, and accelerates reinforcement corrosion, making it a critical factor affecting long-term durability.

Due to its low water-to-binder ratio, high cementitious content, dense internal structure, and low permeability, HPC allows for slow external CO_2_ penetration, resulting in a carbonation shrinkage rate lower than that of ordinary concrete [82,83]. However, once carbonation occurs, the resulting shrinkage deformation is more concentrated, easily forming tensile stress zones on the surface of the member and significantly increasing the risk of surface cracking. Persson [84] systematically demonstrated that active mineral admixtures, including silica fume, can enhance the microstructure of the HPC matrix, decrease permeability, and retard the carbonation process, thus contributing positively to the suppression of carbonation shrinkage.

All the aforementioned types of shrinkage effects can induce microcracks within HPC to varying degrees, but the characteristics of the defects caused by different types of shrinkage differ significantly. For HPC with a low w/b, autogenous shrinkage generates a large number of uniformly distributed primary capillary microcracks within the matrix during the early hardening stage. These microscopic damages, buried deep within the material, do not all manifest on the specimen surface, whereas drying shrinkage primarily induces surface cracks, and the effects of carbonation shrinkage are largely confined to the shallow surface layer of the concrete. Furthermore, the initial internal damage caused by CS, plastic shrinkage, and temperature shrinkage is relatively limited. Therefore, the internal microdefects generated by autogenous shrinkage play a particularly critical role in mechanical degradation under dynamic loading conditions. Under dynamic loading, concrete exhibits significant strain rate sensitivity, and the material’s fracture behavior is highly dependent on the existing initial damage state of the matrix. Primary microcracks left by autogenous shrinkage may undergo only slow, subcritical propagation under static loads, with a relatively limited impact on macroscopic performance. However, under high-strain-rate dynamic loads such as impact, seismic events, and cyclic vibration, stress waves act on existing microdefects, causing microcracks to rapidly initiate, connect, and coalesce, forming through-cracks, which, in turn, lead to a significant decline in dynamic compressive strength, dynamic tensile strength, and impact toughness. Capillary pores within the matrix and microcracks induced by autogenous shrinkage become the preferred pathways for crack initiation and propagation under dynamic stress, significantly lowering the threshold at which HPC resists dynamic load failure [85]. While different supplementary cementitious materials alter the extent of various types of concrete shrinkage, they also improve the matrix’s resistance to dynamic loads; there is a clear trade-off between these two properties. For example, silica fume and nano-silica can refine the pore structure and densify the interface transition zone, effectively enhancing resistance to dynamic loads. However, they increase early-age autogenous shrinkage and make the concrete more prone to the formation of internal primary microcracks [86]. Limestone powder primarily exerts a particle-filling effect and does not significantly alter either the shrinkage behavior or the dynamic mechanical properties [87]. Fly ash and slag can suppress the autogenous and drying shrinkage of concrete, thereby reducing shrinkage-induced damage. However, when used as single admixtures, their effect on improving resistance to dynamic loads is limited. Li et al. [85] experimentally confirmed that primary microdefects within the matrix serve as preferential pathways for crack propagation under impact loads. Compared to the use of a single mineral admixture, a binary blend of fly ash and slag can optimize the microstructure of concrete, effectively suppress the through-cracking of cracks under impact loads, and significantly improve the specimens’ impact resistance and dynamic mechanical properties. Thus, the use of multi-component admixtures offers an opportunity to reconcile the conflict between the dynamic load-bearing capacity and shrinkage deformation in HPC.

## 3. Mechanisms of HPC Autogenous Shrinkage

At present, the prevailing explanations for the mechanism of autogenous shrinkage in HPC can be categorized into three main theories: surface tension theory, disjoining pressure theory and capillary pressure theory. These three theories reveal the microscopic driving forces of autogenous shrinkage at different scales. Among them, the capillary pressure theory is widely recognized as the core mechanism governing HPC autogenous shrinkage due to its solid mechanical and thermodynamic foundations and its high consistency with experimental phenomena [60].

### 3.1. Surface Tension

The surface tension theory is one of the earliest classical mechanisms used to explain volumetric deformation in cement-based materials [88]. This theory posits that when water molecules adsorb onto the surfaces of gel particles, the solid–gas interface is replaced by a solid–liquid interface, resulting in a significant reduction in interfacial energy and a decrease in particle surface tension. This generates expansion stresses that push the particles apart, manifesting macroscopically as volumetric expansion of the paste. Conversely, when the internal relative humidity declines and adsorbed water undergoes desorption, the solid–gas interface re-establishes, causing a marked increase in surface tension. A contraction stress emerges that acts to reduce the system’s specific surface area, thereby drawing gel particles into closer proximity and ultimately inducing macroscopic contraction of the slurry. However, this mechanism acts only on the few layers of adsorbed water near the surface of the gel particles, contributing very little to the total contraction, and is only relatively significant when the relative humidity is very low. Since HPC autogenous shrinkage typically occurs in the range where relative humidity exceeds 75%, surface tension is not the dominant mechanism. It merely provides the interfacial energy basis for the formation of a meniscus.

### 3.2. Disjoining Pressure

**Figure 8:**
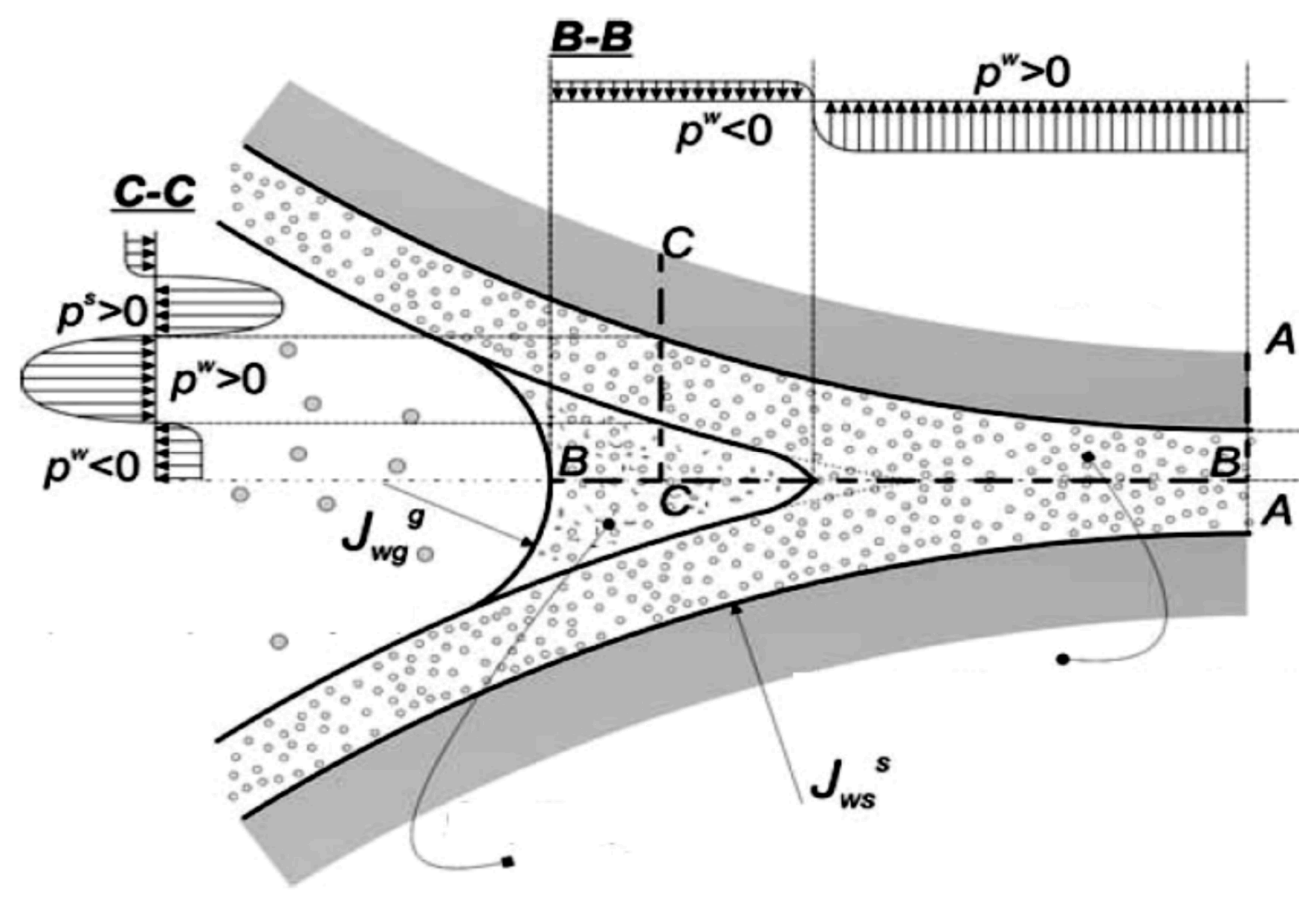
Schematic illustration of the mechanism of disjoining pressure within gel pores [89].

The disjoining pressure theory, based on intermolecular interactions at the solid–liquid interface, explains the microscopic volume deformation within gel pores and ultrafine capillaries. Its essence lies in the mechanical equilibrium of the adsorbed water film in confined gel pores [89], as shown in Figure 8. When the characteristic size of the gel pore (or the interparticle distance between C-S-H gel particles) is less than twice the thickness of the adsorbed water layer (typically <10 nm), the adsorbed water layers on the adjacent solid surfaces overlap. The repulsive force generated by water molecules under the solid–liquid interfacial force field is the disjoining pressure, which is composed of three components: van der Waals forces, double-layer repulsion, and hydration structural forces (short-range structural forces) [90]. The mechanism of this process is related to changes in IRH. Under high-humidity saturated conditions, the adsorbed water layer within the gel pores is intact, and the disjoining pressure in the confined adsorption zone (e.g., the central pore region with (p*^w^* > 0) in cross-section C-C) is significant, sufficient to offset the van der Waals attractive forces between particles, thereby maintaining the stable, separated structure of the C-S-H gel. As self-drying causes the IRH to decrease, the adsorbed water layer gradually thins, and the disjoining pressure decays exponentially. When the repulsive forces become smaller than the attractive forces, the mechanical equilibrium is disrupted, and the direction of solid–water interfacial water flow (*J_ws_^s^*)) drives the gel particles to move closer together, triggering microscopic shrinkage at the nanoscale.

In summary, the disjoining pressure only induces limited nanoscale micro-shrinkage in the early high-humidity stage of gel pores and ultrafine capillaries. It cannot dominate the total amount or rate of macroscopic shrinkage. Therefore, the disjoining pressure theory is not the primary mechanism of HPC autogenous shrinkage.

### 3.3. Capillary Pressure

**Figure 9:**
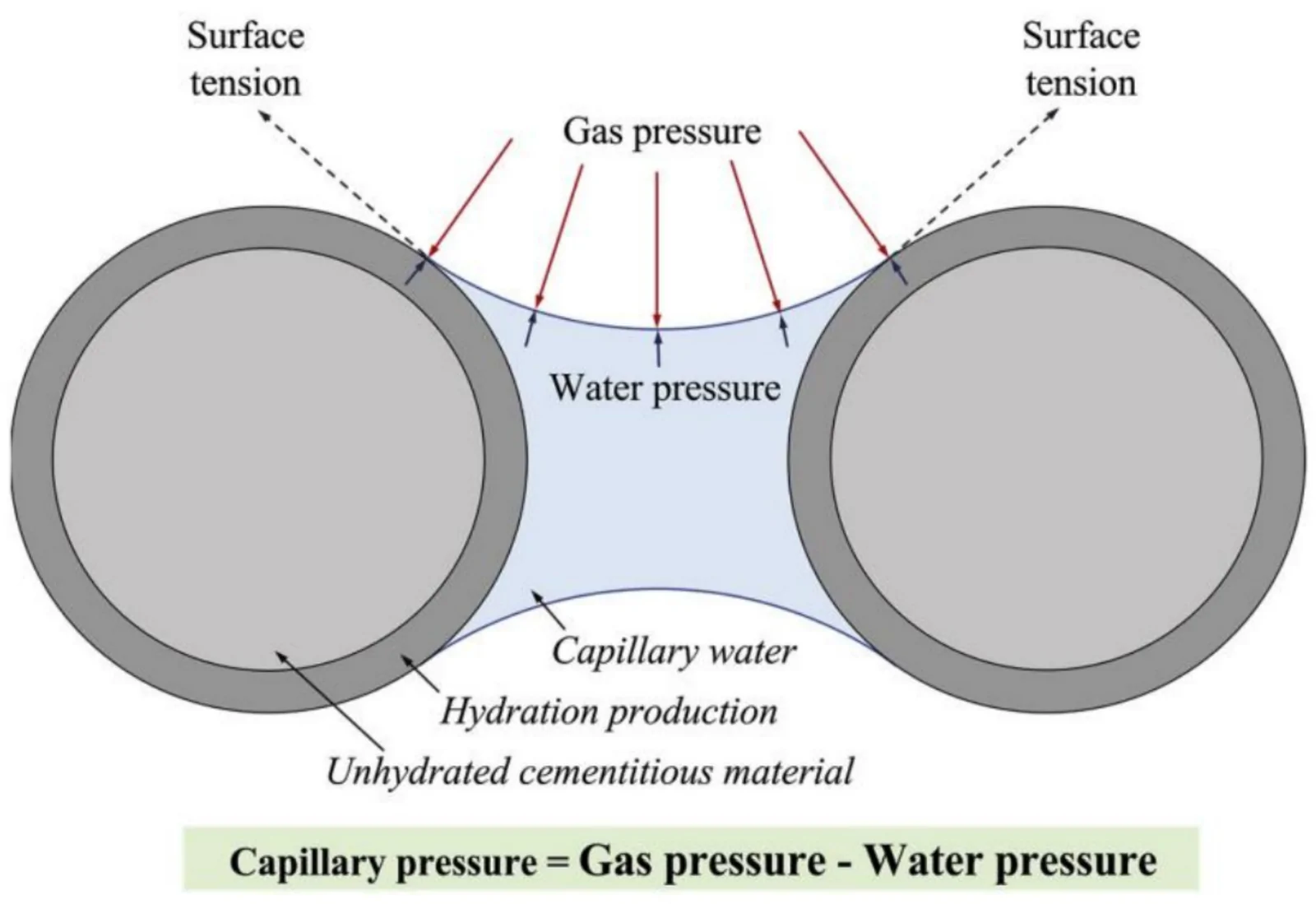
Schematic diagram of capillary negative pressure [91].

Capillary pressure serves as the direct driving force that converts the deformation of the HPC’s microscopic pore structure into macroscopic autogenous shrinkage. As shown in Figure 9, capillary negative pressure results from the combined action of surface tension and the pressure difference at the water-air interface within the capillaries [91]. It can be calculated using the Laplace equation (Equation (1)) and the Kelvin equation (Equation (2)):

(1) $$\begin{array}{c} p_{c}=p_{g}-p_{w}=(2\sigma cos\theta )/r \end{array}$$

(2) $$\begin{array}{c} \frac{M}{\rho }\cdot \frac{2\sigma }{r}\cdot \cos\theta =RT\ln\frac{P_{0}}{P_{r}} \end{array}$$

where: *p_c_*: capillary negative pressure; *p_g_*: gas phase pressure; *p_w_*: pore water pressure; *σ*: surface tension of the gas-liquid interface; *θ*: contact angle; *r*: radius of curvature of the meniscus; *ρ*: density of water; *M*: molar mass of water; *R*: ideal gas constant; *T*: absolute temperature; *P*_0_: saturated water vapor pressure; *P_r_*: actual water vapor pressure.

The capillary pressure method was proposed by Powers [92], who posited that the autogenous shrinkage of concrete is related to the formation of meniscus surfaces in capillary pores during the self-drying process. This method primarily employs pore structure, relative humidity, self-stress, degree of hydration, and interfacial structure to explain the autogenous shrinkage of cementitious materials [12].

#### 3.3.1. Pore Structure

**Figure 10:**
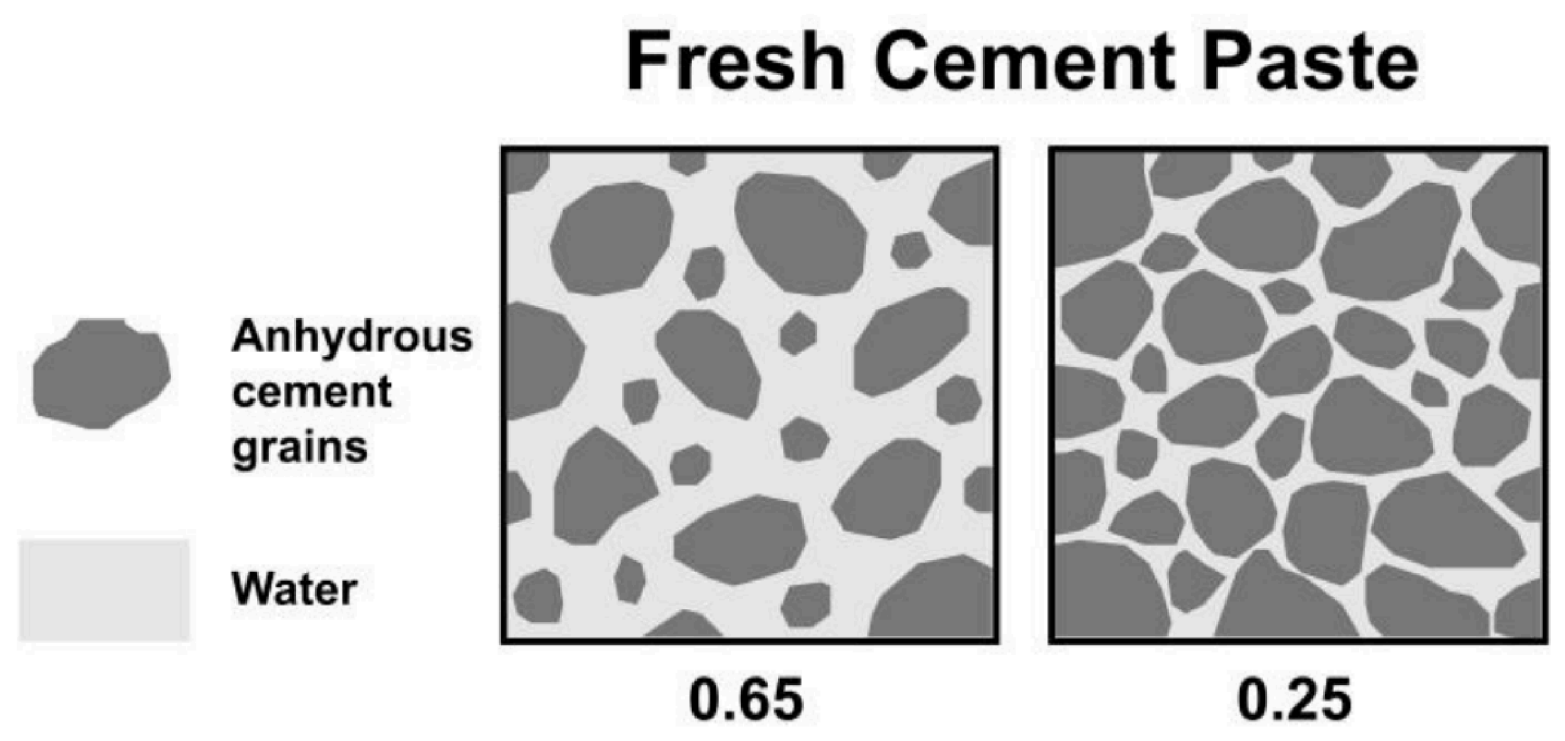
Schematic diagram of the microstructure of fresh cement paste at different water-to-binder ratios [82].

Pore structure serves as the material basis for the generation and development of capillary pressure, directly determining its magnitude and the efficiency of shrinkage transfer. HPC is designed with a low w/b, and its internal structure is dominated by fine capillaries with pore diameters smaller than 50 nm, as shown in Figure 10. In freshly mixed HPC with a w/b of 0.25, the spacing between anhydrate cement particles is much smaller than in ordinary concrete with a w/b of 0.65. The capillaries formed after hydration are finer and denser, providing the structural foundation for the generation of high capillary pressure [93]. Simultaneously, the continuous accumulation of hydration products significantly increases the stiffness of the pore walls. Microscopic shrinkage cannot be offset by pore deformation and must be released through overall matrix deformation, efficiently converting the compressive force of capillary pressure into macroscopic autogenous shrinkage.

**Figure 11:**
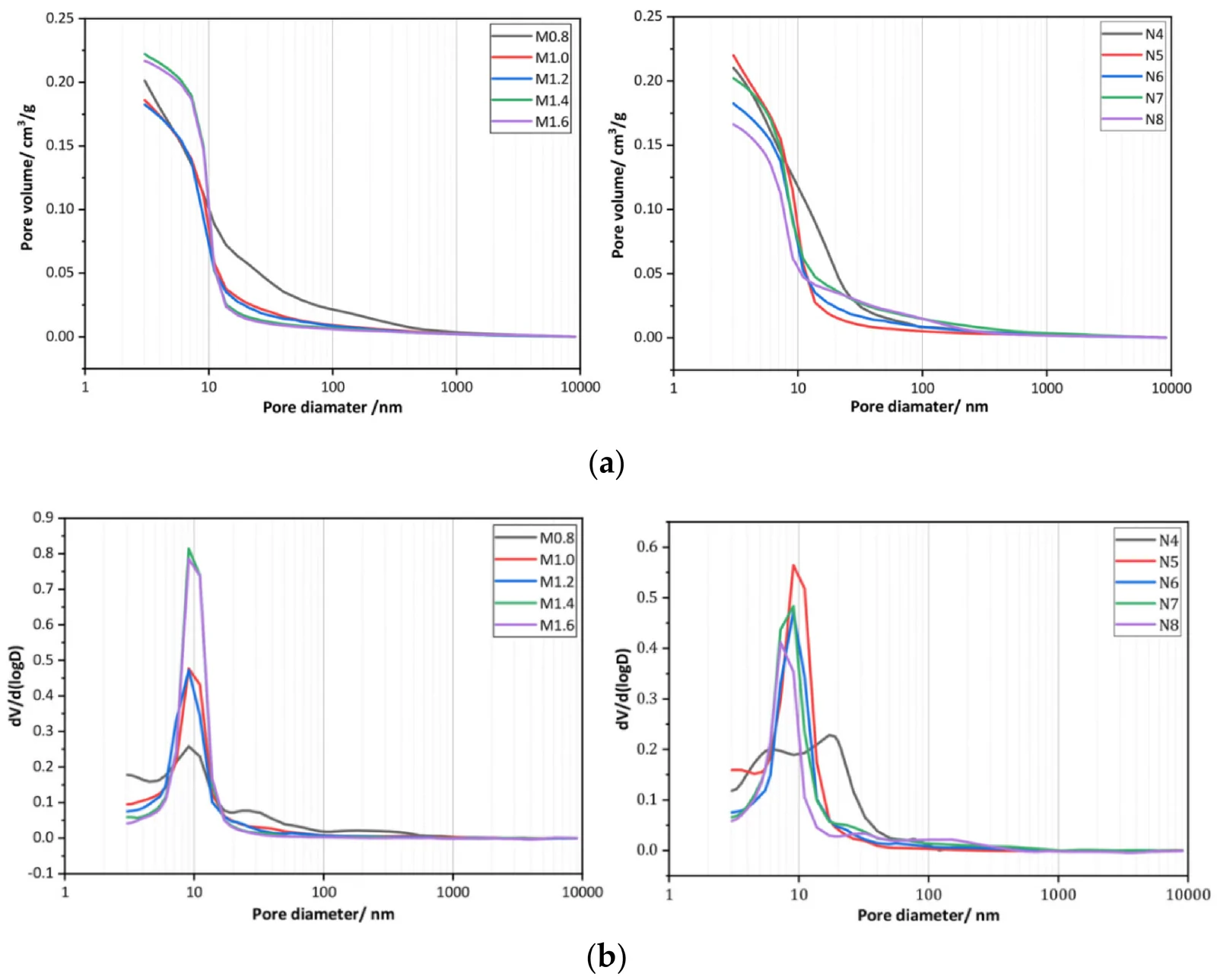
Pore structure of each mixture [95]: (**a**) Cumulative pore volume; (**b**) Pore-size distribution. (M is the sodium silicate modulus, which characterizes the hydration activity of the system, the higher the M value, the more complete the secondary hydration reaction).

SCMs can alter pore size distribution and porosity through physical filling and secondary hydration, and differences in their reactivity produce distinctly different regulatory effects on the pore structure [94]. As shown in the cumulative pore volume curve in Figure 11a, the pore volume in the high-activity systems is significantly higher in the small-nanopore range than in the low-activity systems, indicating a higher proportion of micropores. The differential pore size distribution curve in Figure 11b further shows that as the system activity increases, the peak of the pore size distribution shifts markedly toward smaller pore sizes, reflecting that high-activity admixtures cause excessive refinement of the pore size. According to capillary pressure theory, capillary pressure is inversely proportional to pore size. This microporous pore structure significantly increases capillary pressure, becoming a key factor in enhancing the shrinkage driving force. In contrast, low-activity admixtures can optimize the pore size distribution, reduce the proportion of micropores, effectively lower capillary pressure, and weaken the shrinkage driving force [95].

#### 3.3.2. Relative Humidity

Relative humidity is a key factor affecting the autogenous shrinkage of HPC [96]. In a sealed curing system, the hydration reaction of cementitious materials continuously consumes internal free water without external moisture replenishment, causing the IRH to decrease continuously as hydration progresses. This process is strongly correlated with the formation of capillary pressure. According to the Kelvin equation (Equation (2)), IRH is positively correlated with capillary pore radius. The lower the IRH, the smaller the effective pore diameter participating in capillary action, and the capillary pressure increases exponentially. Studies indicate that water diffusion and self-drying are the two primary causes of IRH decline in cement paste and concrete. Water diffusion is a physical process in which pore water migrates outward due to an external drying environment, while self-drying is a chemical process in which the continuous hydration of cement and SCMs consumes internal free water, leading to a spontaneous IRH decrease in pores. Due to its high w/b (>0.42) and abundant free water, changes in the IRH of ordinary concrete are primarily controlled by water diffusion. Whereas HPC, with its low w/b (<0.42) and highly active cementitious system, exhibits IRH changes jointly controlled by water diffusion and self-desiccation [97,98].

**Figure 12:**
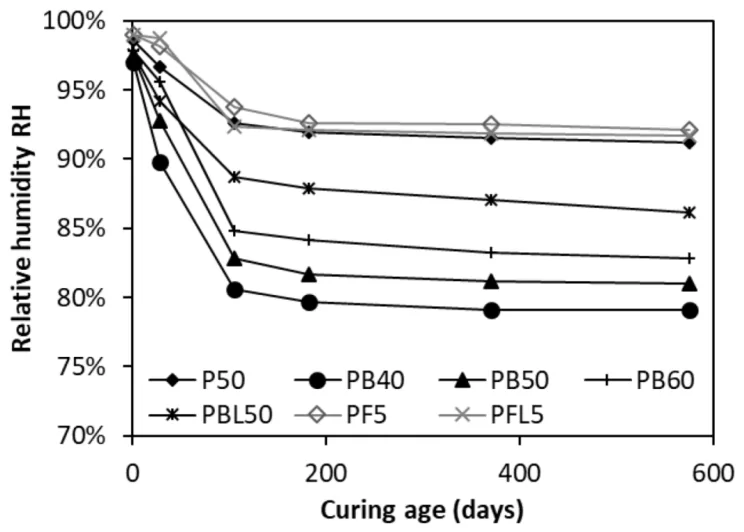
Effect of SCMs on relative humidity in blended pastes [102]. (P50 is the control group consisting of pure cement paste with a water-to-binder ratio of 0.5. PB40, PB50, and PB60 are composite systems containing 30% slag and having water-to-binder ratios of 0.4, 0.5, and 0.6, respectively. PBL50 is a system containing 30% slag blended with 5% limestone powder and a water-to-binder ratio of 0.5. PF5 is a system containing 30% fly ash and a water-to-binder ratio of 0.5. PFL5 is a system containing 30% fly ash blended with 5% limestone powder and a water-to-binder ratio of 0.5.).

SCMs exert a significant regulatory effect on the evolution of IRH in HPC (Figure 12). Studies have shown that the pozzolanic effects of silica fume and slag accelerate early hydration, leading to more intense consumption of capillary water and a significant increase in the humidity drop caused by self-drying [99]. Simultaneously, SCMs can optimize the pore structure, improve the density of the paste, and reduce the rate of water diffusion, thereby reducing the humidity drop caused by external drying [100]. The pore refinement caused by different types of SCMs interacts bidirectionally with the evolution of IRH. The pozzolanic reaction of highly reactive SCMs such as silica fume and blast furnace slag not only accelerate early-stage water consumption and exacerbates moisture loss due to self-drying, but also generates a large number of ultrafine gel pores that restructure the matrix pore structure, increasing the tortuosity of the pore water transport pathways. The rapid consumption of free water in the early stages causes a sharp drop in humidity. However, by the later stages of curing, the pathways for pore water migration outward are blocked by the dense network of fine channels, significantly slowing the rate of decline in IRH. In contrast, low-reactivity SCMs such as fly ash and limestone powder rely solely on particle filling to moderately reduce the proportion of large capillaries without excessively generating nanoscale micropores. At the same time, the dilution effect slows the overall rate of hydration water consumption, allowing the system to maintain a higher relative humidity throughout the curing stage, thereby significantly attenuating the increase in capillary pressure caused by self-drying [101]. It is evident, therefore, that differences in the reactivity of the SCMs determine the extent of pore refinement, and the refined pore structure, in turn, alters the conditions for pore water consumption and migration. These two factors interact and jointly determine the long-term evolution of relative humidity within HPC.

#### 3.3.3. Degree of Hydration

The degree of hydration determines the development process and intensity of capillary pressure and serves as the time-driving source of autogenous shrinkage. The higher the degree of hydration, the faster the consumption of free water, the more significant the decrease in IRH, the earlier the formation of the meniscus, and the faster the initiation of capillary pressure. At the same time, hydration products continuously fill the pores, continuously refining the capillary pore size, and further amplifying the capillary pressure effect. Although HPC with a low w/b has limited hydration, its early hydration rate is high, allowing it to rapidly generate high capillary pressure. Studies have shown that highly reactive admixtures (silica fume, nano-silica) enhance early hydration, accelerate self-drying, and increase autogenous shrinkage. Whereas low-reactivity admixtures (fly ash) delay hydration, reduce the rate of humidity decline, and thereby suppress autogenous shrinkage [103]. In addition to pozzolanic materials, the mineral composition of cement—along with the cement type—significantly influences the hydration process and autogenous shrinkage characteristics of HPC. Cements with high levels of tricalcium silicate (C_3_S) and tricalcium aluminate (C_3_A) exhibit faster early-age hydration rates, which accelerate the consumption of pore water and the self-drying process, thereby resulting in more pronounced autogenous shrinkage. In contrast, low-heat-of-hydration cements reduce the proportion of C_3_S and C_3_A minerals, thereby slowing early-stage hydration reactions and maintaining relative humidity within the matrix, which effectively mitigates early-stage autogenous shrinkage. Furthermore, cement fineness also plays a significant role. Cements with higher fineness have larger hydration reaction interfaces, promoting rapid early-stage hydration and further exacerbating autogenous shrinkage deformation in HPC with a low w/b.

For determining the degree of hydration, the isothermal calorimetry method (ASTM C1702-14 [104], EN 196-11 [105]) uses a microcalorimeter to continuously record the exothermic curve of hydration. By combining the cumulative heat released with standard reference values, the degree of hydration at different ages is calculated. This method is particularly suitable for studying the early stages of the hydration reaction and for comparing the effects of different SCMs. Since this method is typically limited to small-volume specimens and relatively short test durations, various testing methods capable of quantitatively determining the degree of hydration in cementitious systems have emerged in recent years, including thermogravimetry—Thermogravimetric Analysis (TG-DTG) relies on the weight loss from the dehydration of hydration products to quantitatively calculate the content of non-evaporable bound water, which is then used to estimate the overall degree of hydration. By identifying characteristic weight loss peaks of calcium hydroxide and C-S-H gel in the 40–400 °C range, it distinguishes the proportions of different hydration products and can accurately identify the slow hydration characteristics of low-activity systems such as fly ash and limestone powder [106]. X-ray diffraction combined with Rietveld refinement (XRD-Rietveld) can quantitatively determine the residual amount of unhydrated clinker minerals and directly calculate the clinker hydration ratio. This method can simultaneously distinguish the calcium hydroxide content consumed by the secondary hydration of mineral admixtures and is suitable for monitoring the long-term degree of hydration in multi-component composite cementitious systems [107]. Low-field nuclear magnetic resonance (LF-NMR), as a non-destructive testing method, uses pore water relaxation signals to distinguish the proportions of free water, adsorbed water, and chemically bound water. It enables continuous in situ monitoring of the hydration process of HPC throughout its entire aging period under sealed curing conditions, thereby addressing the limitation of signal attenuation in later stages observed with calorimetric methods [108]. Furthermore, the SEM point-counting method calculates the degree of hydration by statistically analyzing the area proportion of unhydrated particles in microscopic images; the error relative to thermogravimetric and calorimetric test results can be controlled within 10%, making it suitable as a cross-validation method among multiple techniques [109].

#### 3.3.4. Interfacial Structure

**Figure 13:**
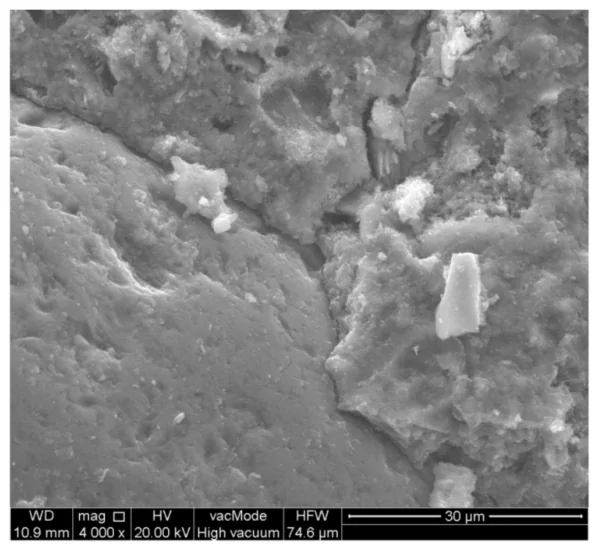
SEM micrograph of the aggregate-paste interfacial transition zone in high w/b concrete [110].

**Figure 14:**
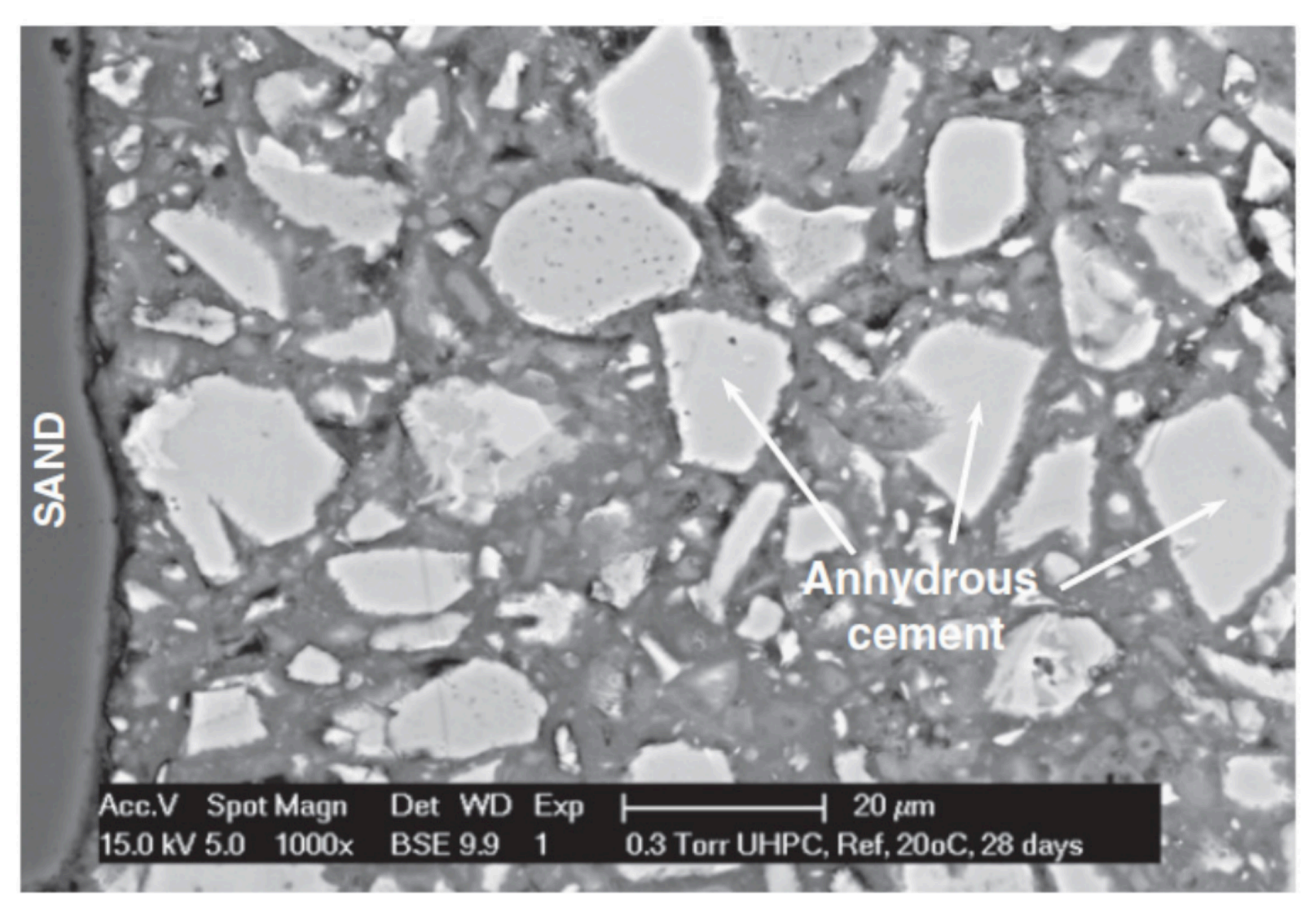
BSE micrograph of the aggregate-paste interfacial transition zone (ITZ) in low w/b concrete at 28 days [111].

The interface structure primarily refers to the aggregate-paste interface transition zone (ITZ), which is the critical region influencing the uniformity of shrinkage and the extent of stress concentration. The ITZ typically features large pores and a loose structure, exhibiting significant differences from the matrix pore structure. This leads to an uneven distribution of capillary pressure, resulting in deformation differences and stress concentration, making it a vulnerable site for autogenous shrinkage cracking. As shown in Figure 13, the microstructure of high w/b concrete is characterized by high porosity and heterogeneity in the matrix, with oriented Ca(OH)_2_ (CH) crystals present in the paste near the aggregate surfaces, and a distinct weak interface zone [110]. In contrast, Figure 14 shows that there is no distinct interface transition zone between the sand aggregate and the cement paste in HPC [111]. The matrix is dense and homogeneous, with anhydrous cement particles uniformly distributed in the hydration products. Even in air-entrained HPC, the cement paste remains dense, with a fine pore structure and low connectivity, resulting in a fundamental improvement in the interface bonding state. Since the weak interface zone is eliminated, cracking in HPC concrete begins with the propagation of mortar cracks rather than interface cracks [82].

SCMs, through microfiller filling and secondary hydration, can optimize the interfacial transition zone, reduce interfacial porosity, and enhance density. This results in more uniform shrinkage deformation, reduces stress concentration, delays the formation and propagation of microcracks, and improves the concrete’s resistance to autogenous shrinkage cracking [112].

## 4. Patterns of the Influence of SCMs on the Autogenous Shrinkage of HPC

Recent years have witnessed extensive experimental and mechanistic research by numerous scholars on the effect of SCMs on the autogenous shrinkage of HPC. Research indicates that SCMs regulate the hydration process, pore structure, and microstructure of HPC through physical effects (filling, dilution) and chemical effects (pozzolanic reaction, hydration synergy), thereby significantly altering its autogenous shrinkage behavior [113,114,115,116,117,118,119,120,121]. From a physical perspective, in HPC with a w/b of 0.25–0.30, small capillaries (5–50 nm) account for 71–80% of the total pore volume, whereas when the w/b increases to 0.4, this volume percentage drops to 36–47% [118,122]. It is generally believed that a higher proportion of small capillaries is the primary factor causing greater autogenous shrinkage in low w/b concrete. From a chemical perspective, HPC with a low w/b and high cementitious material content typically lacks sufficient water for complete cement hydration, whereas cementitious composites with a high w/b are closer to complete hydration. Studies have shown that the degree of hydration of a cement paste with a w/b of 0.30 is only 76–83% of that of a cement paste with a w/b of 0.50 [118,123]. In contrast to cementitious materials with a low w/b, a reduction in moisture increases the sensitivity of cement hydration to competition for water from the pozzolanic reaction. This sensitivity may lead to a greater influence of the pozzolanic reaction on shrinkage [124].

### 4.1. Fly Ash

#### 4.1.1. The Effect of Fly Ash on the Autogenous Shrinkage of HPC

As one of the most widely used industrial by-product admixtures, research indicates that fly ash, as a mineral admixture, primarily exerts a suppressing effect on the autogenous shrinkage of HPC. However, this suppressing effect is not constant and is related to factors such as the type of fly ash, dosage, particle size distribution, concrete w/b, and curing age. The quantitative effects of autogenous shrinkage suppression under different test conditions are shown in Table 1.

**Figure 15:**
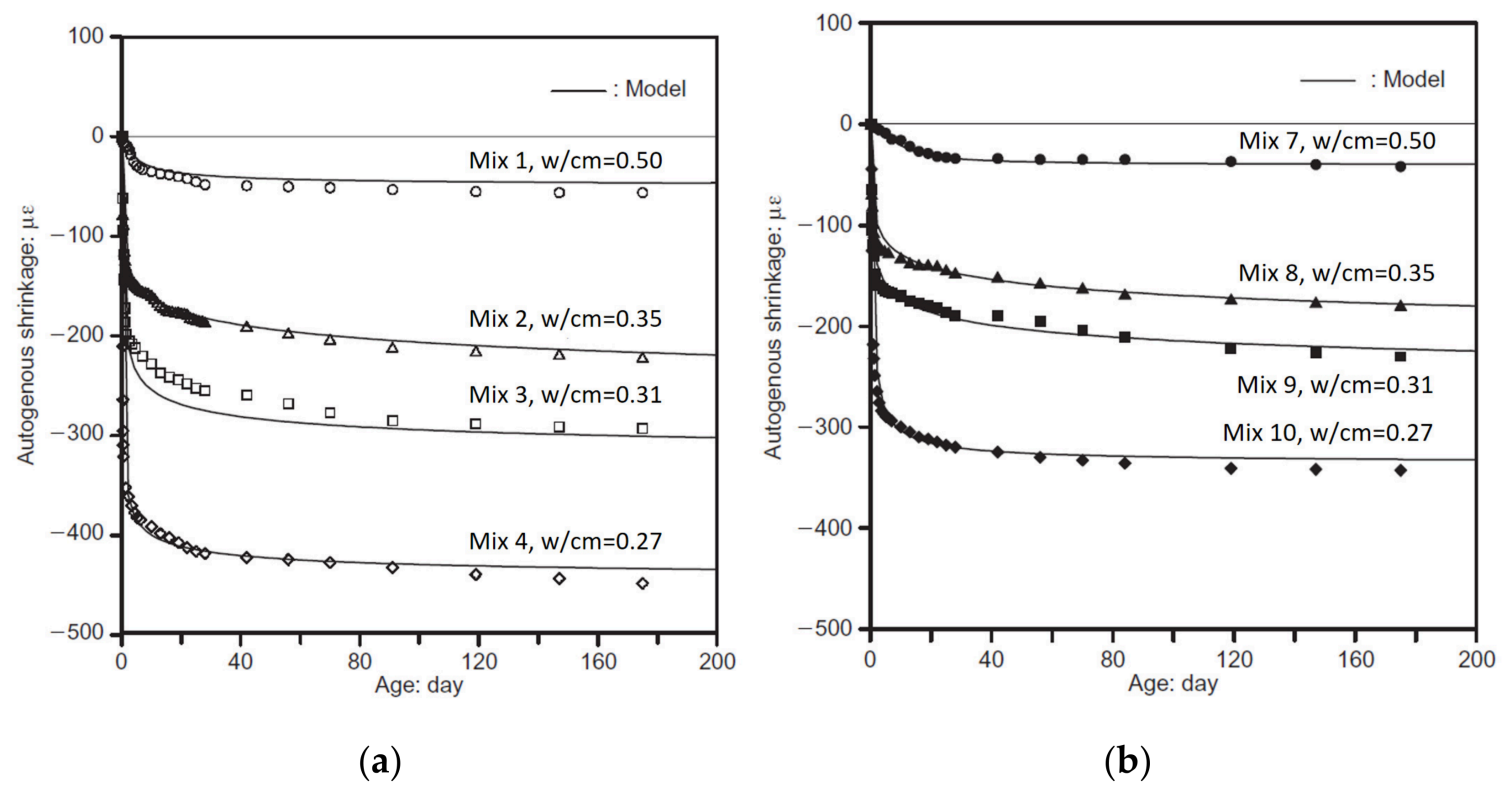
Effect of w/cm on autogenous shrinkage for (**a**) OPC and (**b**) FA20 concretes [127]. (The w/cm adopted in the figure corresponds to the w/b defined in this study.).

**Figure 16:**
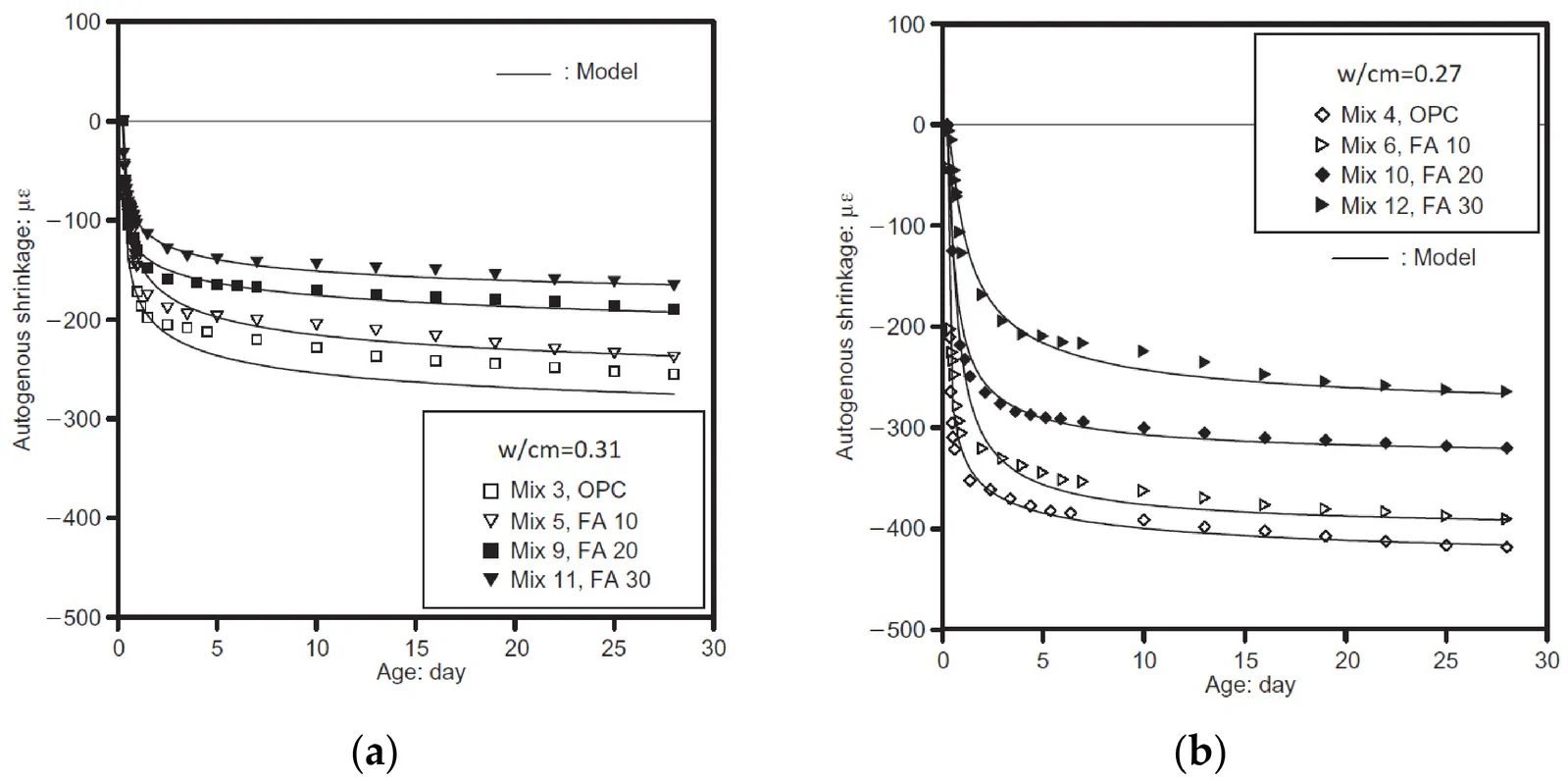
Effect of fly ash content on autogenous shrinkage of concrete with a low w/cm: (**a**) 0.31 and (**b**) 0.27 [127]. (The w/cm adopted in the figure corresponds to the w/b defined in this study.).

The dosage is the key factor in regulating the effect of fly ash on suppressing the autogenous shrinkage of HPC. Its inhibitory effect increases significantly with higher dosages. An et al. [125] conducted a study on Class I fly ash, in an HPC system with a w/b of 0.33, they replaced cement with fly ash at dosages of 10%, 20% and 30%, and tested the autogenous shrinkage at 7 days of age. The results showed that autogenous shrinkage decreased by 19.8%, 32.8% and 37.1%, respectively, with increasing dosage. Lee H K et al. [127] studied Class F fly ash and found that, at a w/b of 0.30 and a curing age of 3 days, autogenous shrinkage was reduced by 23.9% and 58.7% for fly ash dosages of 15% and 45%, respectively. Furthermore, the regulatory effect of fly ash is more pronounced in systems with low w/b [125,126,127]. For the same Type I fly ash at a 30% dosage and 7-day age, the reduction in autogenous shrinkage was 41.0% at a w/b of 0.26, which is significantly higher than the 32.8% reduction observed at a ratio of 0.33. The effect of fly ash on the autogenous shrinkage of HPC exhibits a “strong early, weak late” characteristic in terms of curing age. Jiang et al. [73] found that, at a w/b of 0.30 and a 30% dosage of fly ash, Type F fly ash reduced autogenous shrinkage by 49.5% at 7 days, but by only 23% at 60 days. The inhibitory effect of Class I fly ash at 28 days was also lower than that observed in the early stages, which was attributed to the later-stage pozzolanic reaction offsetting part of the physical inhibitory effect [126,133]. The type of fly ash also affects the regulatory effect, low-calcium fly ash (Class F) exhibits a better inhibitory effect on the autogenous shrinkage of HPC than high-calcium fly ash. Class I fly ash demonstrates a better inhibitory effect than Class II due to its finer particle size and spherical particle shape, while Class F has a broader range of applicability. Niken [134] investigated the low-dosage effect of Type F fly ash in a system with a w/b of 0.35, at a 10% dosage, the reduction in 7-day autogenous shrinkage reached 57.0%. As shown in Figure 15, in a system with a 20% fly ash, autogenous shrinkage increased significantly as the w/b decreased, but remained lower overall than that of pure cement concrete under the same conditions. Figure 16 further indicates that, at the same w/b, the autogenous shrinkage of HPC continuously decreases as the fly ash dosage increases. The shrinkage-inhibiting effect gradually strengthens within the 10–30% fly ash dosage range, demonstrating a clear dependence on the dosage.

In summary, the effectiveness of fly ash in suppressing the autogenous shrinkage of HPC increases significantly as the dosage increases and the w/b decreases but gradually weakens with age. Furthermore, Class I fly ash outperforms Class II fly ash, and Class F fly ash exhibits superior shrinkage-suppression stability. However, when discussing the regulatory effect of fly ash on the autogenous shrinkage of HPC, it is also necessary to consider the coupled synergistic effects of particle fineness, curing regimen, and dosage. Conventional fly ash has relatively low reactivity, it can effectively suppress autogenous shrinkage through the physical filling and dilution effects of its spherical particles. Within a reasonable dosage range of 10% to 45%, the reduction in autogenous shrinkage gradually increases as the fly ash dosage rises. However, when the dosage exceeds 45%, the persistent pozzolanic reaction during the mid-to-late stages continuously refines the matrix’s microporous structure, causing a slight rebound in the concrete’s long-term autogenous shrinkage. In contrast, ultrafine fly ash has a large specific surface area and intense pozzolanic reactivity, in sealed curing systems, it rapidly consumes internal free water and refines the capillary pore structure, which instead leads to increased early-stage autogenous shrinkage [135]. Differences in regulation resulting from curing conditions are particularly pronounced. In a purely sealed system, the internal self-drying effect is significant, allowing the shrinkage-reducing effect of fly ash to be fully realized. In contrast, internal curing media such as base ash and SAP can continuously supply moisture to the matrix, maintaining high relative humidity in the system over the long term and significantly attenuating the development of capillary pressure, thereby substantially offsetting the shrinkage-reducing contribution of fly ash [136,137]. If the system is blended with a magnesium-based expansive agent, the shrinkage-regulating effect of fly ash is further weakened [138]. Therefore, in engineering applications, the fly ash dosage should not exceed 45%. In addition to the rational selection of mineral admixture blends, a curing regimen appropriate for the actual service conditions should be adopted. When necessary, shrinkage-reducing agents and expansive components may be co-blended to employ a multi-pronged approach that effectively controls autogenous shrinkage while ensuring the development of early-age concrete strength.

#### 4.1.2. Mechanism Underlying the Effect of Fly Ash on Autogenous Shrinkage of HPC

**Figure 17:**
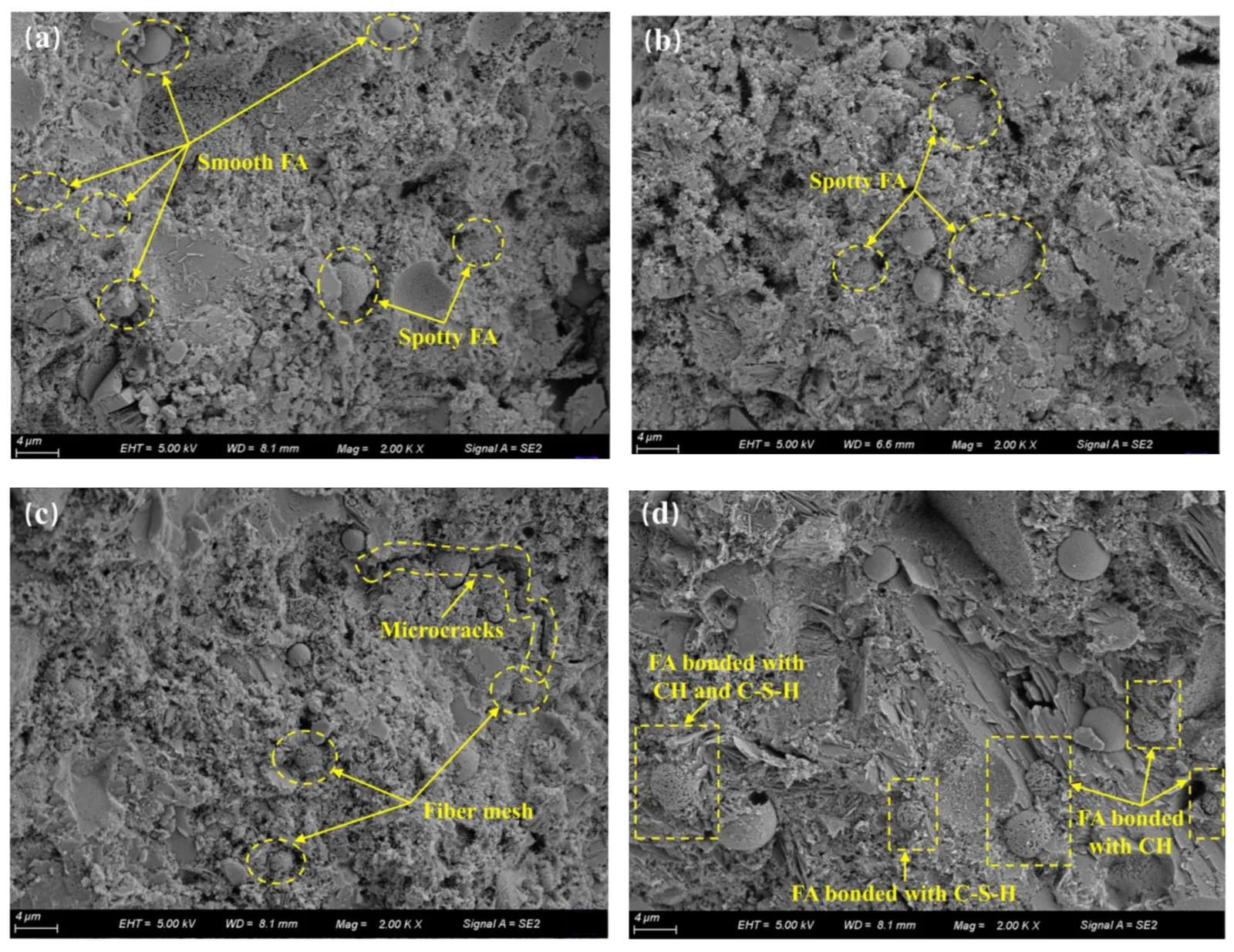

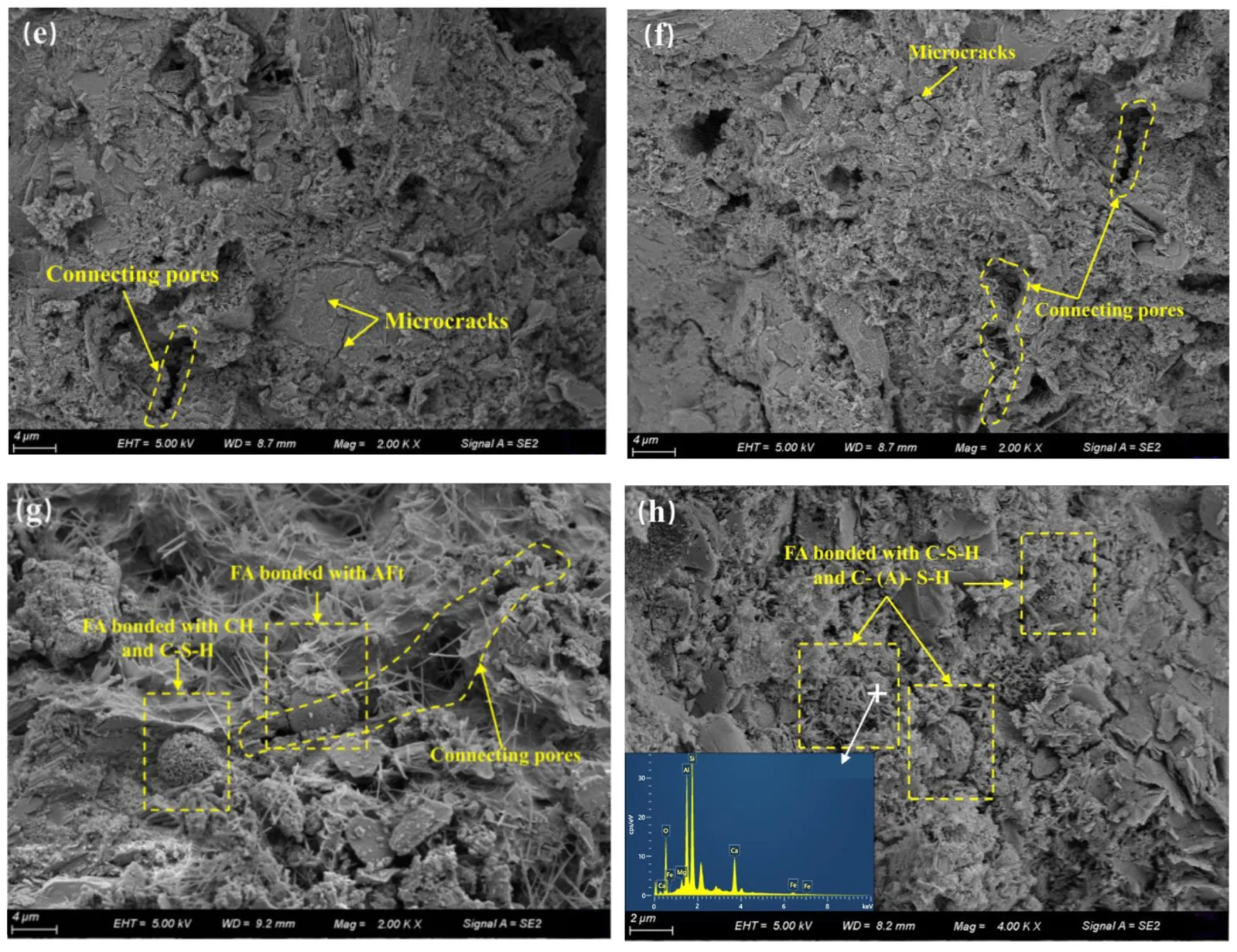
SEM image of concrete. Images (**a**–**d**) are F20 maintained under steam curing for 6 h, 12 h, are 28 days and 90 days after steam curing of F20. 24 h and 36 h, respectively. Images (**e**,**f**) are 24 h and 28 days after steam curing of F0. Images (**g**,**h**) are 28 days and 90 days after steam curing of F20 [142].

The regulatory effect of fly ash on the autogenous shrinkage of HPC stems from the synergistic interaction between its physical filler effect and chemical effect (pozzolanic reaction) [139,140,141]. This process can be visually confirmed through the evolution of the microstructure of concrete at different hydration ages (Figure 17). During the early hydration stage, unreacted fly ash particles exhibit a smooth spherical morphology (Figure 17a) and can uniformly fill the capillary pores in the cement paste. This effectively reduces the number of large and interconnected pores, optimizes the pore size distribution of the matrix, and lowers internal capillary pressure, thereby significantly suppressing early-stage autogenous shrinkage. As the hydration age increases, the surfaces of fly ash particles are gradually eroded in the alkaline environment, developing a speckled, rough morphology (Figure 17b) and progressively forming fibrous, network-like hydration products (Figure 17c). Concurrently, these products form tight bonding interfaces with hydration products such as Ca(OH)_2_ and C-S-H, and ettringite (AFt) generated during cement hydration (Figure 17d–h). This pozzolanic reaction process not only consumes Ca(OH)_2_ crystals—which are prone to causing interfacial defects—but also promotes the formation of more C-S-H gel with a lower calcium-to-silica ratio, while significantly enhancing the matrix density and the bonding performance of the interfacial transition zone. Comparing the microcracks and interconnected pore structures within the matrix without fly ash (Figure 17e,f), it is evident that fly ash, through the synergistic action of early-stage physical filling and late-stage continuous pozzolanic reaction, effectively improves the internal pore structure of the concrete, reduces stress concentration points caused by shrinkage, and achieves long-term regulation of HPC autogenous shrinkage.

### 4.2. Slag

#### 4.2.1. The Effect of Slag on Autogenous Shrinkage of HPC: Underlying Patterns and Mechanisms

In HPC systems with a low w/b, numerous experimental studies have confirmed that slag promotes autogenous shrinkage, and this shrinkage-promoting effect increases significantly as the dosage increases and the w/b decreases. Liu et al. [130], in their study of HPC with a low w/b (0.24), confirmed that increasing the slag dosage leads to a continuous increase in autogenous shrinkage. The core mechanism lies in the fact that the pozzolanic reaction of the slag refines the capillary pore structure, significantly increasing capillary negative pressure and thereby intensifying the self-drying effect. Jiang et al. [73] further discovered through experiments conducted at different curing temperatures that the autogenous shrinkage of slag concrete is more sensitive to temperature, an increase in temperature accelerates the hydration process, further amplifying the shrinkage effect of the slag. Research by Lim et al. [143] indicates that high-fineness slag, when used at high dosages, can increase 28-day autogenous shrinkage by 20–40%, and the rate of shrinkage development is significantly accelerated.

**Figure 18:**
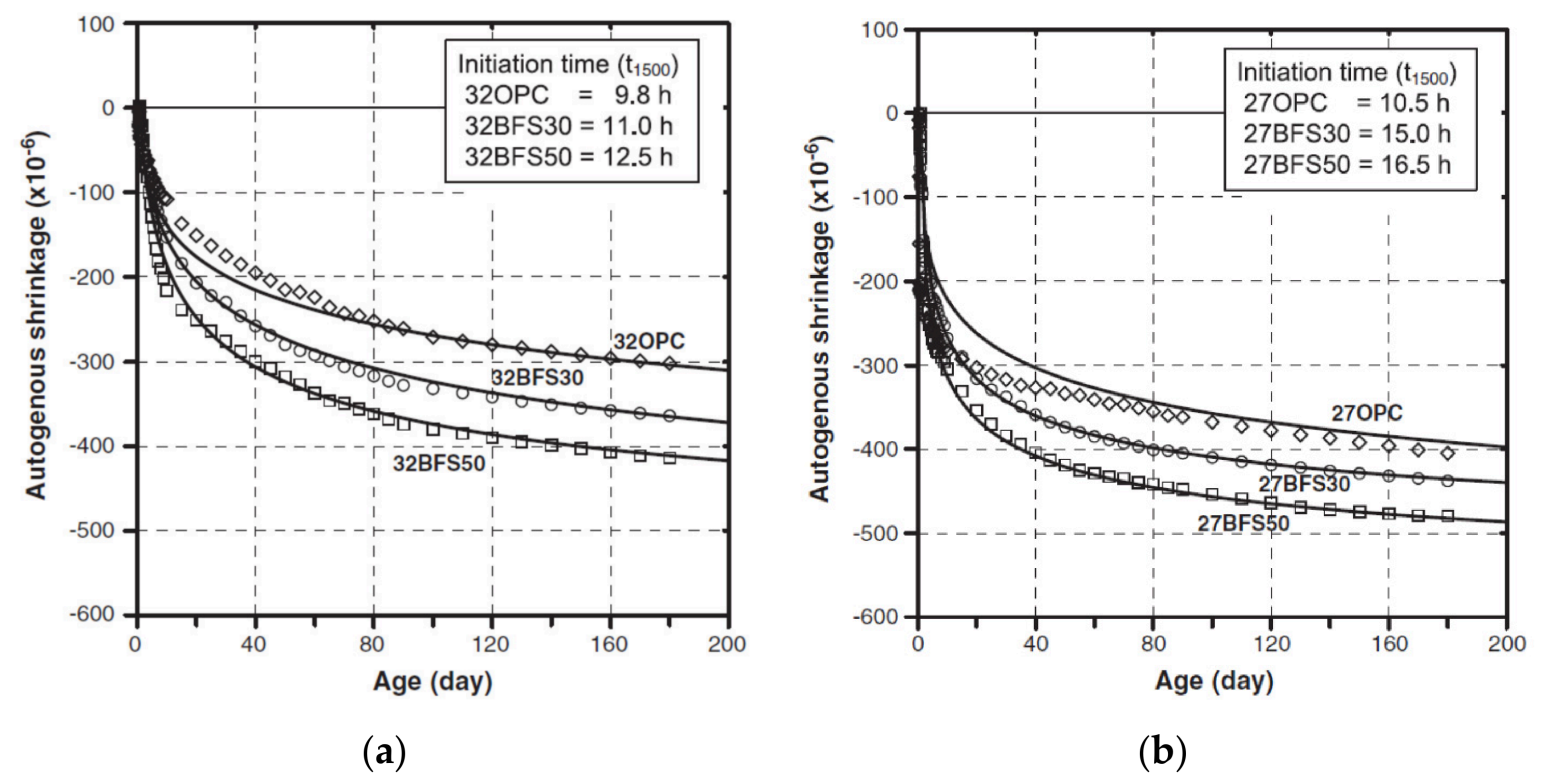
Effect of slag content on autogenous shrinkage of concrete with low w/cm ratio adopted from [127]: (**a**) w/b = 0.32; (**b**) w/b = 0.27.

Overall, existing studies generally agree that slag promotes the autogenous shrinkage of HPC at low w/b, with the extent of shrinkage being influenced by the dosage, w/b, fineness, and curing conditions. Although slag exhibits a certain diluting effect in the early stages, the ongoing pozzolanic reaction in the mid-to-late stages continuously consumes water and refines the pore structure, ultimately leading to an increase in total autogenous shrinkage. As shown in Figure 18, under various combinations of w/b and slag dosages, the autogenous shrinkage of the slag groups consistently exceeds that of the pure cement groups. Moreover, the lower the w/b and the higher the dosage, the more pronounced the increase in autogenous shrinkage.

#### 4.2.2. Analysis of Hydration Products and Microstructure

**Figure 19:**
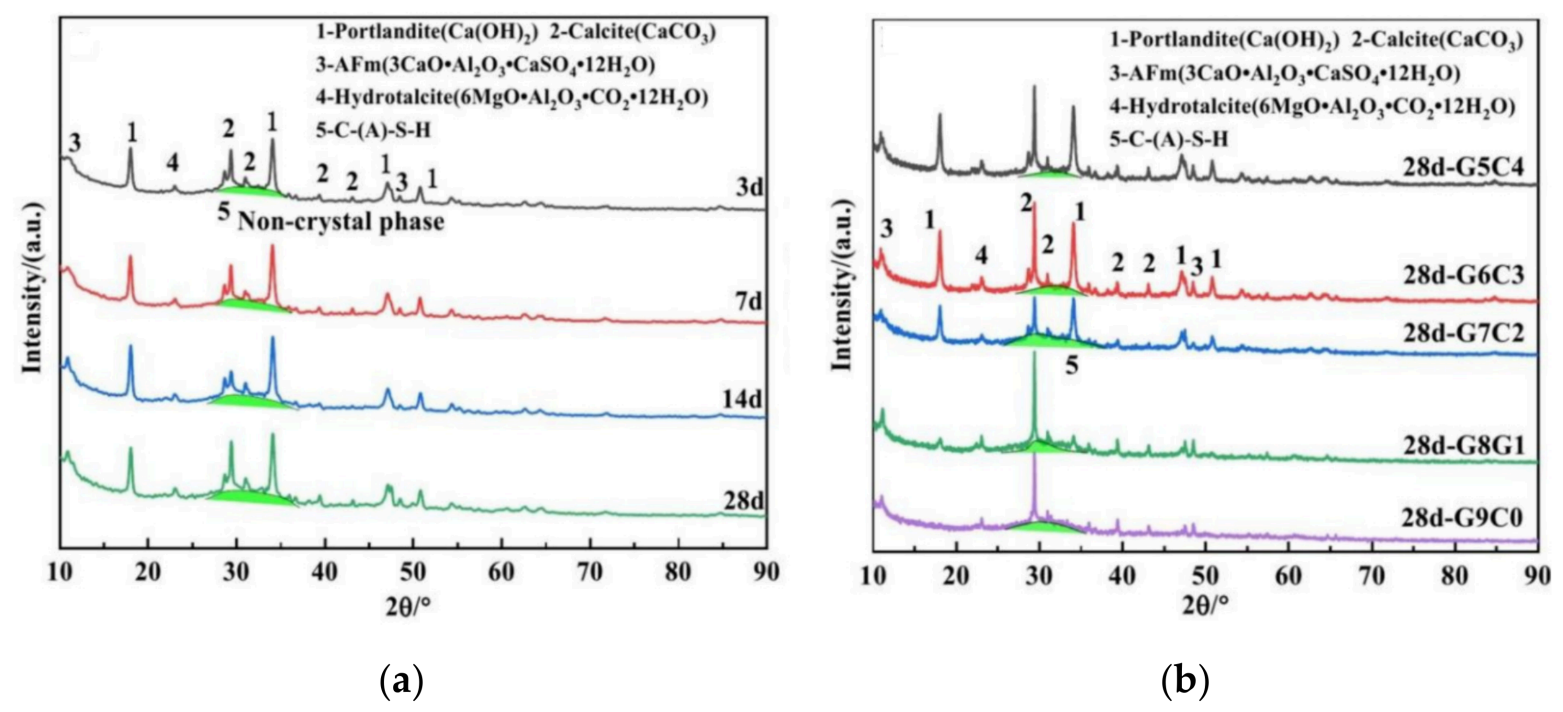
XRD patterns of GCCM samples [144]. (**a**) Different ages of G7C2; (**b**) Different n(CaO)/n(SiO_2_  +  Al_2_O_3_).

Figure 19 shows the XRD diffraction patterns of slag-cement composite cementitious material (GCCM) specimens. Figure 19a illustrates the phase evolution of a slurry with the same mix ratio at 3, 7, 14, and 28 days of age, while Figure 19b compares the hydration products at 28 days of age for different slag-cement mix ratios. The figure shows that early-stage (3-day) hydration in the slag-cement system is dominated by clinker hydration, resulting in distinct crystal peaks for calcium hydroxide (CH) and alunite. At this stage, the slag reaction is relatively weak, and the characteristic peaks of the C-(A)-S-H gel are not prominent. As the curing period extends (7–14 days), the slag undergoes pozzolanic reaction in the alkaline environment, resulting in a significant decrease in the intensity of the CH peak and an increase in the diffuse C-(A)-S-H gel peak. By 28 days, the system is dominated by C-(A)-S-H gel with a low calcium-to-silica dosage, exhibiting a denser structure. The higher the slag dosage, the more pronounced the consumption of CH and the greater the amount of gel formed [144].

**Figure 20:**
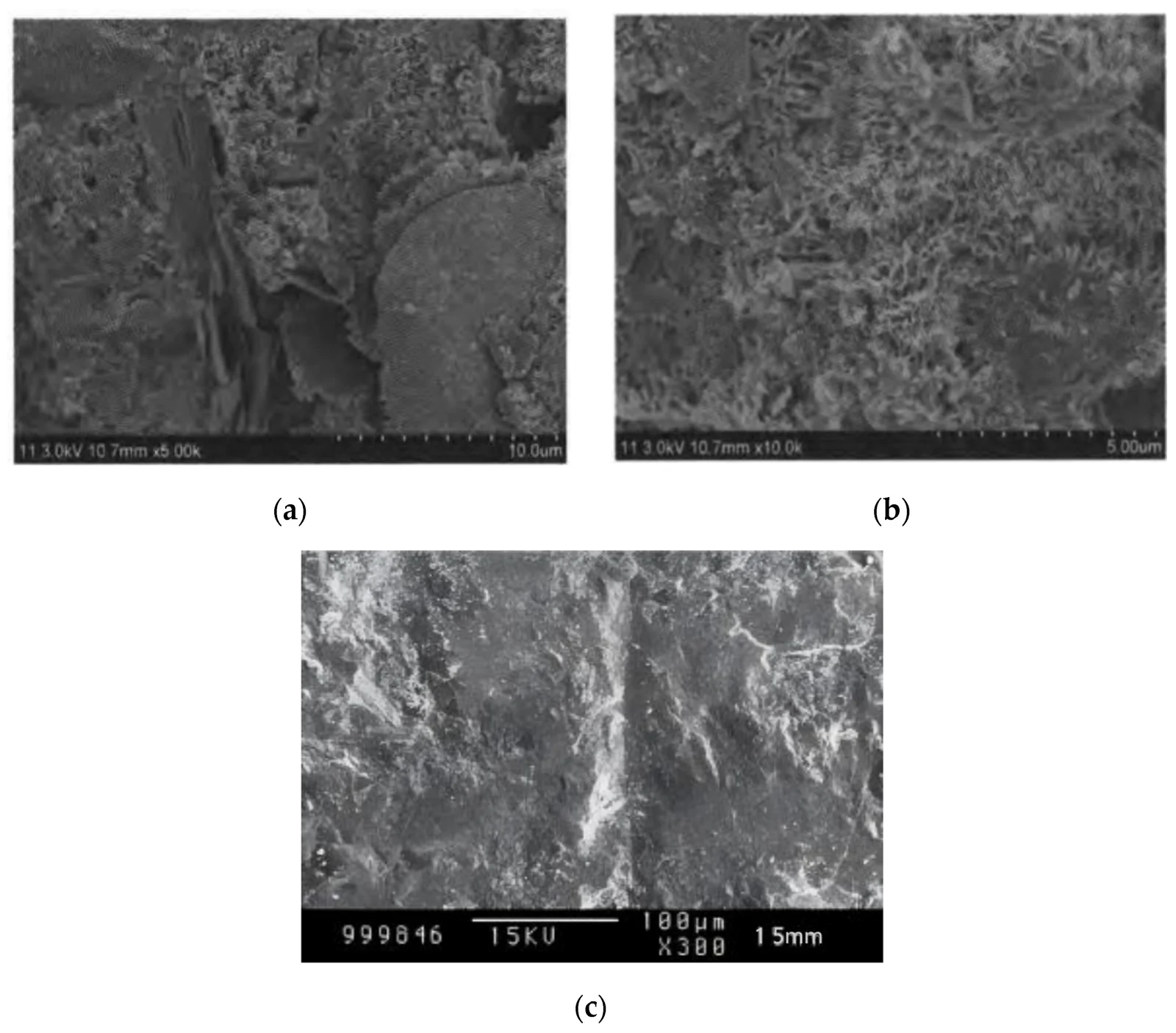
Microscopic morphology of hardened paste of slag-cementitious materials [145]: (**a**) 3 d; (**b**) 7 d; (**c**) 28 d.

Zhang et al. [145] conducted SEM analysis on a mortar with a typical engineering slag dosage of 50%, as shown in Figure 20. Figure 20a shows the microstructure after 3 days of curing. It can be observed that the slag-cement composite paste has a loose structure, with slag particles dispersed in the matrix in the form of smooth flakes. The particle edges are clearly distinguishable, and the interface transition zone is distinct. No obvious hydration products are observed covering the particles, indicating that the degree of slag reaction is low at this stage and that the slag primarily serves a physical filling function. Figure 20b shows the morphology after 7 days of curing. A large amount of C-S-H gel is visible on the surfaces of the slag particles, gradually enveloping them; the interface between the slag and the matrix has become blurred, and the overall structural density has significantly increased. Figure 20c shows the morphology after 28 days of curing. At this stage, the slag particles and hydration products have become tightly intertwined, large pores have significantly decreased, and the weak interface zones have been effectively reinforced. The mechanism is as follows: as the curing age increases, the alkalinity of the pore solution rises, activating the glass phase of the slag. Ca^2+^ and Mg^2+^ are leached out, causing the glass network to disintegrate. Active silica and alumina are released and react with CH, continuously generating C-S-H and ettringite, leading to the continuous densification of the matrix. This finding is also broadly consistent with the experimental and modeling results available in the literature [146,147].

Overall, slag modifies the hydration process, phase composition, and interfacial structure of the paste through the synergistic effects of physical dilution, alkali-activated pozzolanic reactions, and micro-aggregate filling. Furthermore, the effect of slag on regulating the autogenous shrinkage of HPC exhibits distinct phased characteristics and is significantly age-dependent. During the early stages of hydration, slag primarily exerts a physical dilution effect by reducing the proportion of cement clinker, thereby slowing the rates of exothermic hydration and water consumption in the system. This can, to a certain extent, alleviate early-age self-drying and inhibit the development of early-age autogenous shrinkage. However, the degree of hydration and activation of the slag itself is constrained by the alkalinity of the pore solution. If the slag has high reactivity or its dosage is excessive, the early hydration process accelerates, which also intensifies the consumption of water within the pores and, conversely, increases early autogenous shrinkage. As the concrete enters the long-term aging stage, slag continues to undergo pozzolanic reactions, continuously consuming calcium hydroxide and generating low-calcium silicoaluminate hydration products. This process continuously refines the pore structure of the matrix, the ongoing refinement of the pores increases capillary pressure, gradually offsetting the shrinkage-reducing benefits provided by the early-stage dilution effect, and ultimately poses a risk of inducing an increase in long-term autogenous shrinkage.

### 4.3. Silica Fume

**Figure 21:**
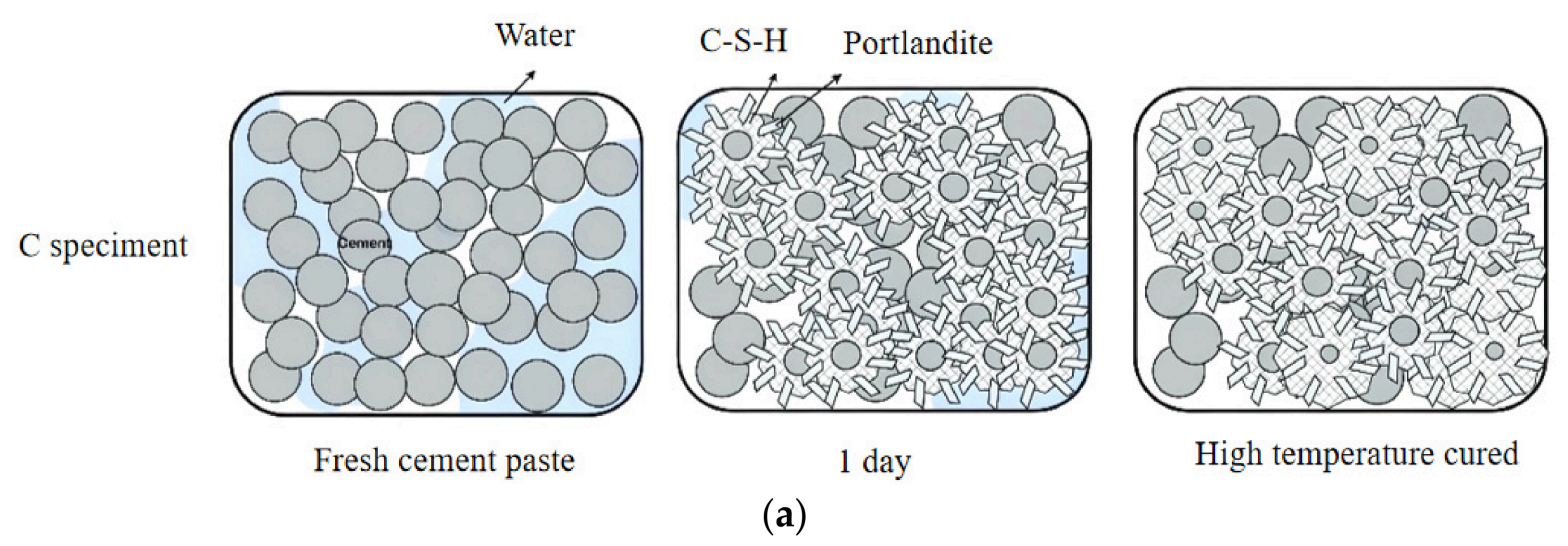

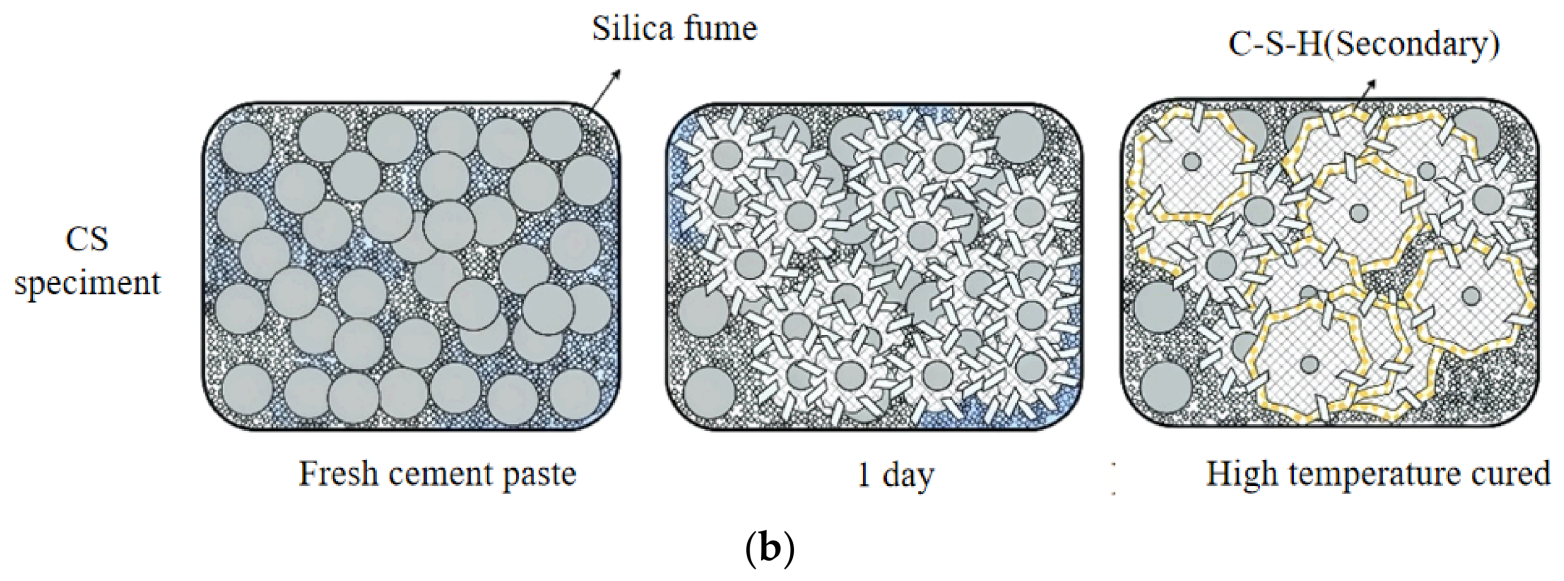
Schematic illustration of the microstructural evolution of different cement pastes during hydration and high-temperature curing: (**a**) Pure cement paste; (**b**) Cement paste with silica fume.

Silica fume is a typical admixture for preparing HPC and ultra-high-strength concrete, and is virtually indispensable in high-strength concrete of C80, C100, and higher grades. Silica fume possesses extremely high pozzolanic activity and a strong micro-aggregate filling effect. Figure 21 illustrates the microstructural evolution of cementitious materials with and without silica fume.

Compared to ordinary cement paste (Group C), silica fume-blended paste (Group CS) reduces initial porosity through ultrafine particle filling during the fresh stage. In the early hydration phase, it accelerates the formation of C-S-H gel and consumes calcium hydroxide. Following high-temperature curing, it further forms secondary C-S-H gel, achieving a highly dense paste structure. While this microstructural densification significantly enhances mechanical properties, it also alters the internal capillary pore structure and the development patterns of relative humidity, thereby exerting a critical regulatory effect on the autogenous shrinkage behavior of HPC. Extensive research indicates that the regulation of HPC autogenous shrinkage by silica fume is primarily an enhancing effect, and this enhancement exhibits significant dependence on the dosage and sensitivity to the w/b.

**Figure 22:**
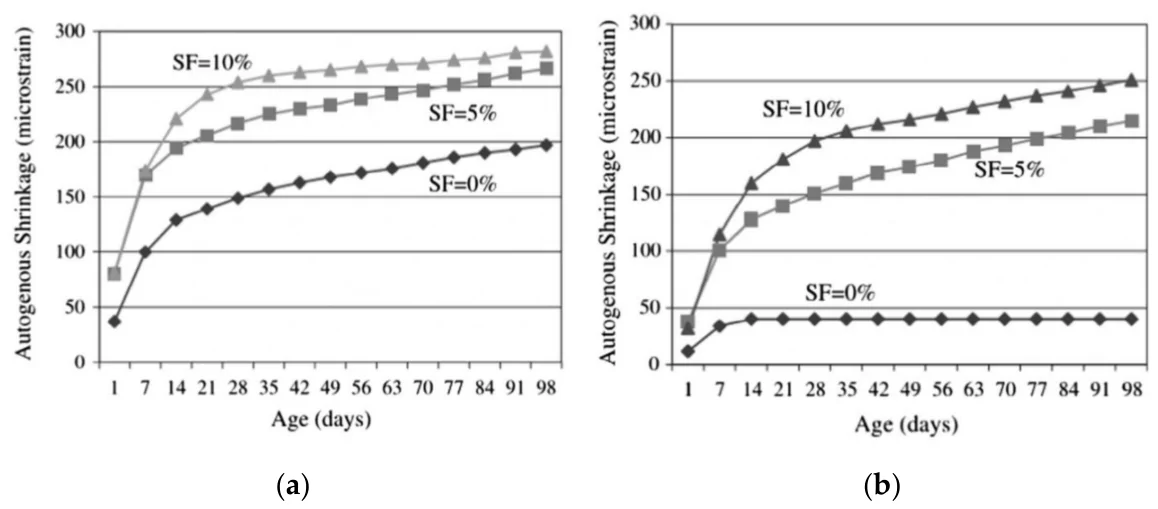
Effect of silica fume content on autogenous shrinkage of concrete with low w/b from [151]: (**a**) w/b = 0.26; (**b**) w/b = 0.35.

The higher the silica fume dosage, the more significant the increase in autogenous shrinkage. Shen et al. [148] investigated the dose effect of silica fume in a system with a w/b of 0.33, at 5%, 10% and 15% dosage, the 3-day autogenous shrinkage increased by 12.5%, 24. 8% and 36.2%, respectively, while the 7-day autogenous shrinkage increased by 18.3%, 31.6% and 45.7%, respectively. The shrinkage effect is more pronounced in systems with low w/b, at a w/b of 0.20, the 10% silica fume dosage resulted in 23.8% increase in 7-day autogenous shrinkage [149], which is significantly higher than the increase observed in a system with a w/b of 0.40. Moreover, the effect of autogenous shrinkage is concentrated in the early stages. Akcay et al. [150] tested the early-age effects in a system with a w/b of 0.35, and found that 2-day autogenous shrinkage increased by 19.6% with a 10% silica fume dosage. Figure 22 illustrates the effect of 5% and 10% silica fume dosage on autogenous shrinkage at two different w/b. It can be observed that the autogenous shrinkage increases with the increase in silica fume dosage, and this effect is more pronounced when a lower w/b is used [151].

**Figure 23:**
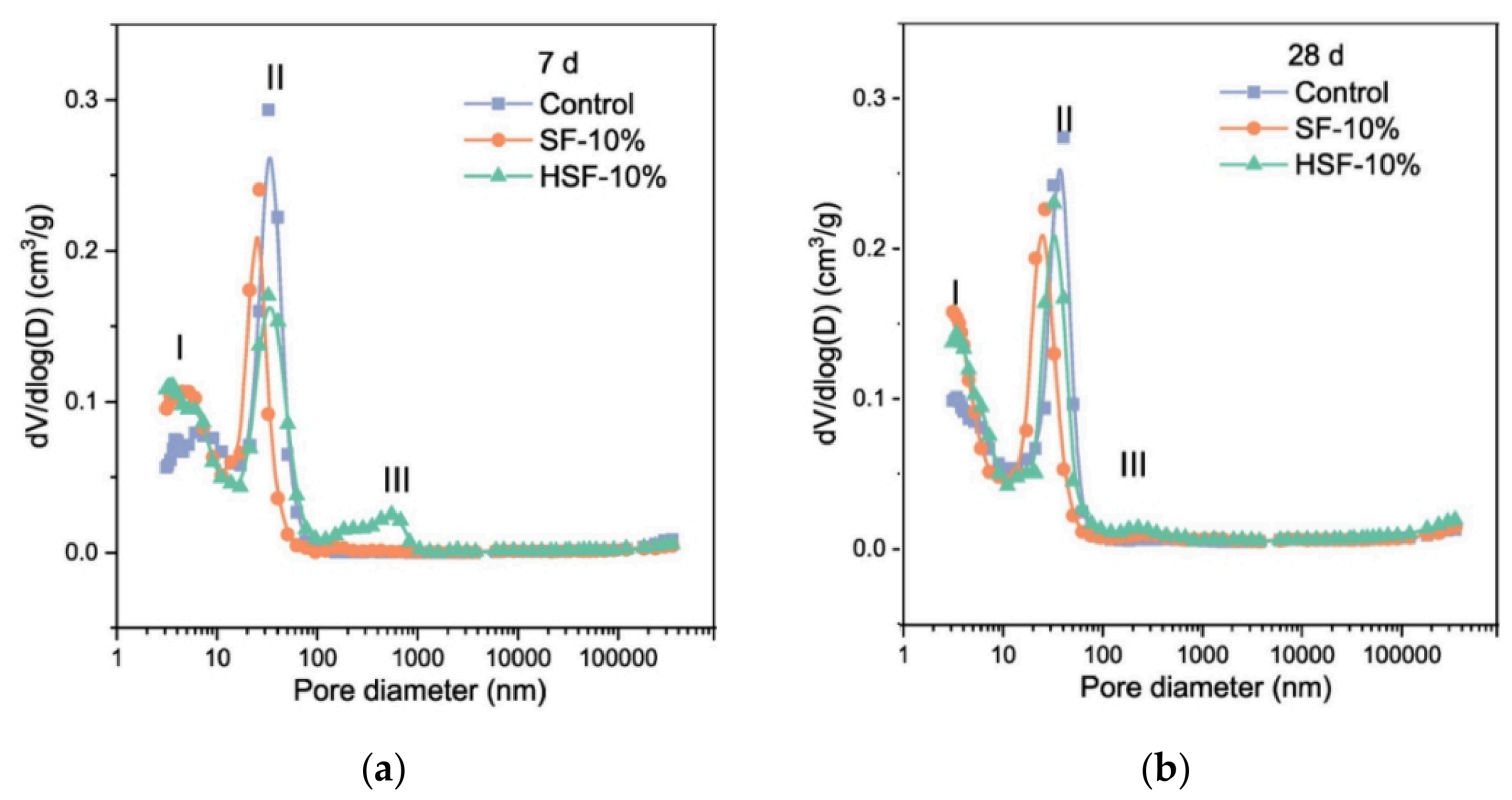
Micropore size distribution curves of cement pastes blended with ordinary silica fume (SF-10%) and hydrophobic-modified silica fume (HSF-10%) [93]: (**a**) 7 d, (**b**) 28 d.

**Figure 24:**
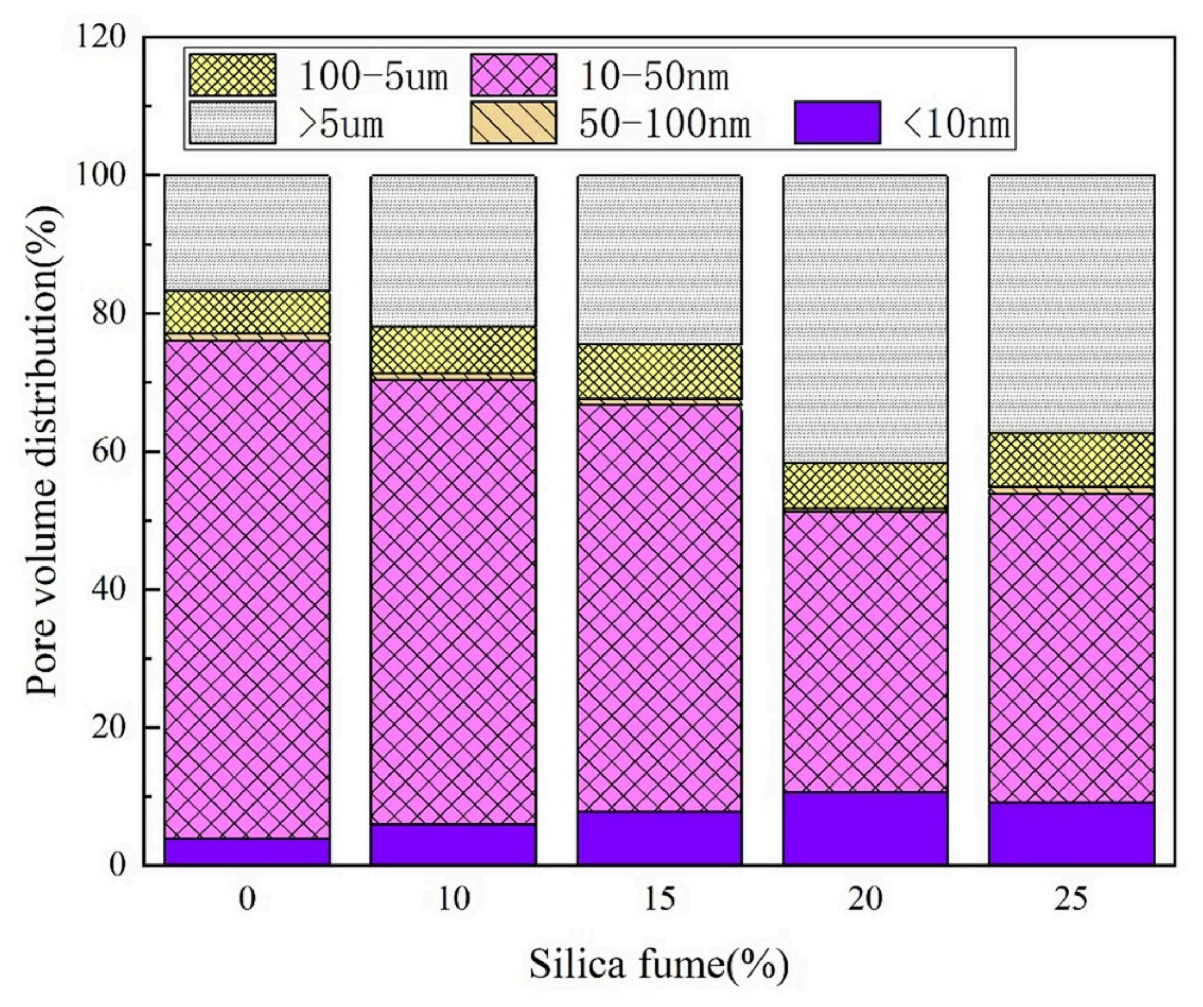
Pore volume distribution in different pore size ranges of concrete with varying silica fume contents [152].

The promoting effect of silica fume on the autogenous shrinkage of HPC is the result of the coupling of its physical and chemical effects. From a temporal perspective, these two effects exhibit a sequential dominance pattern: First, highly reactive pozzolanic reactions accelerate the consumption of free water, intensifying the self-drying effect in systems with low w/b and causing a rapid decrease in IRH. Second, the combined filling of fine pores by microfine aggregates and hydration products refines the capillary pore structure. The reduced pore size leads to an exponential increase in capillary negative pressure, and the dense pore walls facilitate the conversion of micro-shrinkage into macro-deformation. Third, the overall hydration process is accelerated, increasing both the cumulative amount and rate of CS, thereby intensifying the volume deficit. Consequently, the synergistic interaction of these two mechanisms—acting in succession during the early and late stages—collectively results in the total shrinkage of the silica fume-blended system typically being higher than that of the control group. Zhang et al. [93] compared the effects of ordinary silica fume and hydrophobic-modified silica fume (HSF) on the pore structure of cement paste. As shown in Figure 23, the characteristic peaks I, II, and III within the curve represent gel pores, interstitial pores between hydration products, and capillary pores, respectively. The results indicate that ordinary silica fume exhibits high early-stage pozzolanic reactivity, which refines the interstitial pores among hydration products and reduces the total pore volume. In contrast, HSF inhibits the pozzolanic reaction and delays cement hydration. Combined with the water-repellent effect of the silane hydrophobic groups, this ultimately leads to coarser pore sizes and a significant increase in total pore volume, with the degree of pore structure degradation even exceeding that of the blank control specimen. The pore size distribution results at different silica fume dosages (Figure 24) further revealed the quantitative characteristics of this process: as the silica fume dosage increased from 0% to 20%, the proportion of gel pores (<10 nm) and transition pores (10–50 nm) in concrete increased continuously, while the proportion of macropores (>5 μm) decreased significantly, showing a positive correlation between pore refinement and silica fume dosage, which provides direct support for the mechanism that silica fume promotes autogenous shrinkage by refining the pore structure and increasing capillary negative pressure [152]. However, it should be noted that this pattern may be influenced by the cementitious system. For example, in low-heat Portland cement (LHP) systems, due to their inherently lower heat of hydration and the fact that the shrinkage driving force caused by the self-drying effect differs from that of ordinary Portland cement (OPC), studies have shown that at a silica fume dosage of 8%, a favorable balance between increased hydration rate and optimized pore structure can be achieved in such systems, resulting in relatively low shrinkage [153].

Based on existing research, it is recommended that the silica fume dosage in engineering applications be controlled between 5% and 10%. It is typically blended with shrinkage-inhibiting admixtures such as fly ash and conventional slag, or combined with internal curing and early-stage moisture-retention curing techniques, to offset its early-age shrinkage effect and achieve synergistic optimization of mechanical and shrinkage properties [154,155,156,157].

### 4.4. Limestone Powder

As a commonly used mineral admixture, limestone powder significantly affects the autogenous shrinkage of HPC and ultra-high-performance concrete (UHPC). This effect is primarily determined by a combination of physical filling effects, nucleation effects, dilution effects, and weak chemical reactions with aluminates. Li et al. [158] found that, under conditions where consistency in workability is maintained, limestone powder can reduce the water content of the mixture through a “mineral plasticizing effect” and optimize particle packing density. When the limestone powder dosage was 20% and 40%, the total free shrinkage of the concrete was similar to that of the control group. However, when the dosage was increased to 60% and 80%, the cement was significantly diluted, resulting in reduced hydration products, a weakened self-drying effect, and a marked decrease in autogenous shrinkage. Research by Kang et al. [159] on ultra-high-performance fiber-reinforced concrete similarly confirmed that when 25–50% limestone powder is used to replace cement, the filling effect of the limestone powder works in synergy with the diluting effect of the increased effective water-to-binder ratio, effectively mitigating autogenous shrinkage. Based on various standards, the optimal limestone powder dosage is generally considered to be between 15% and 35% [160,161].

**Figure 25:**
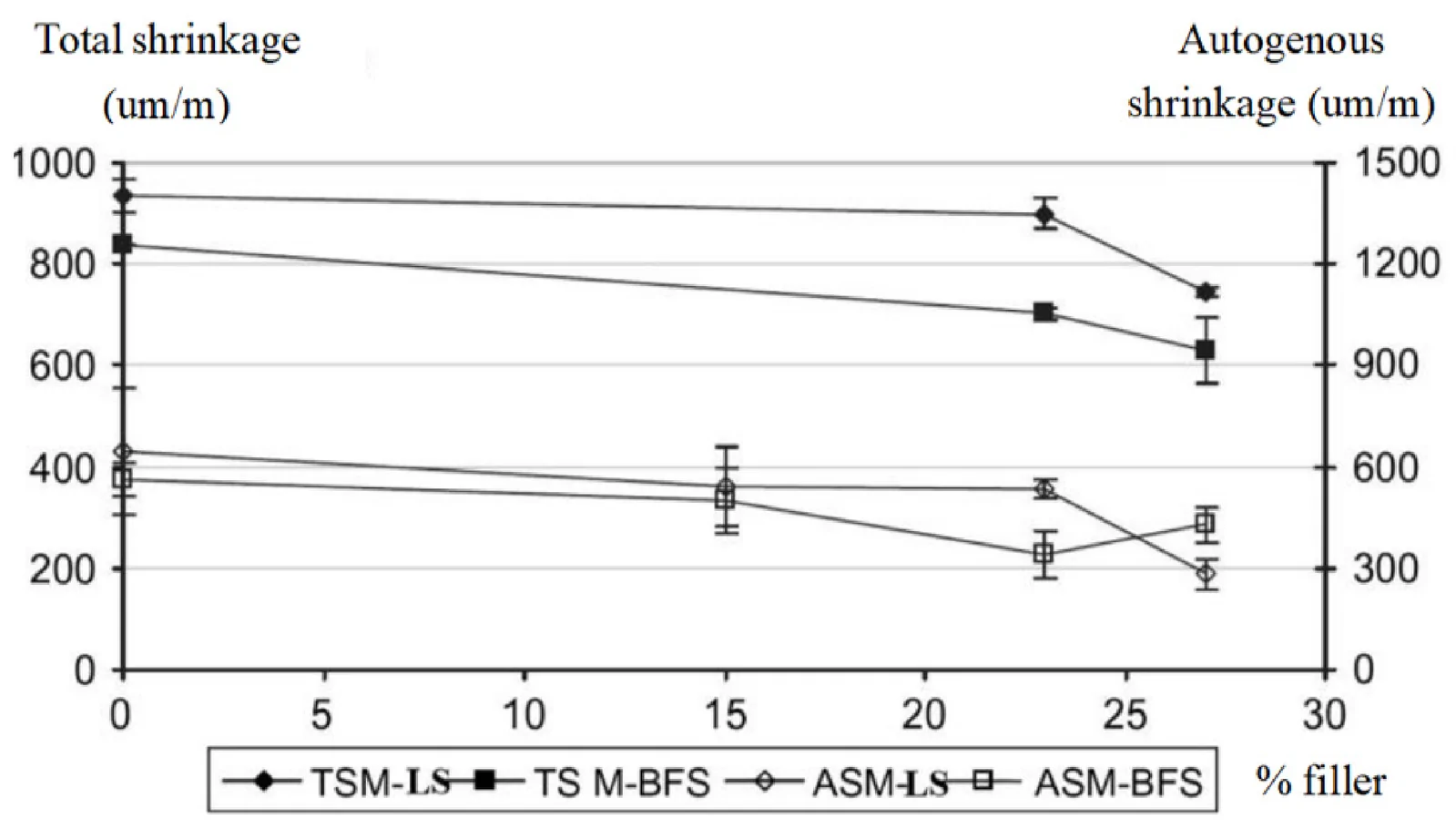
Total and autogenous shrinkages after 28 days for concrete [165].

**Figure 26:**
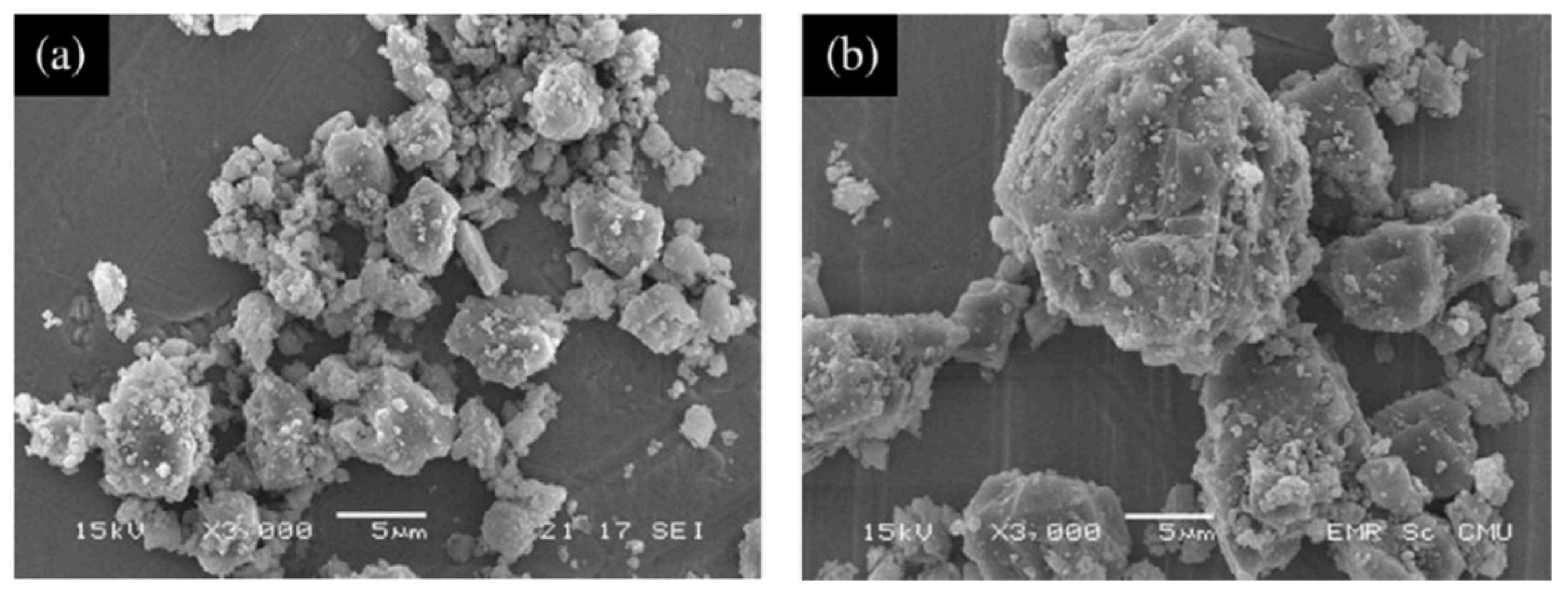
SEM images of LS with particle size of (**a**) 5 μm and (**b**) 20 μm [168].

Figure 25 clearly illustrates the relationship between limestone powder dosage and concrete shrinkage behavior. The effect of limestone powder on shrinkage exhibits a distinct dosage effect. At low dosages, limestone powder acts as a nucleating agent, accelerating the early hydration of cement and slightly increasing early-age autogenous shrinkage [162]. When the dosage exceeds 10%, the dilution and filling effects become dominant: cement consumption decreases, the rate of decline in IRH slows, capillary pressure decreases, and autogenous shrinkage consequently decreases. The finer the limestone powder, the more pronounced the effect of pore structure refinement and the more stable the shrinkage-reducing effect [163]. This competitive transition between the nucleation effect at low dosage levels and the dilution/filling effect at high dosages has been systematically summarized by Wang et al. [164], who clearly pointed out that the mechanism of action of limestone powder depends primarily on particle size and dosage. The study by Jia et al. [165] on 3D-printed UHPC corroborates these findings, demonstrating that a limestone powder dosage of 10% results in the densest pore structure and minimal autogenous shrinkage. when the dosage exceeds 20%, internal defects increase and dimensional stability deteriorates. Additionally, finer limestone powder particles provide better filling and shrinkage-reduction effects, but excessively fine particles can impair forming and stacking performance. Although fine limestone powder can enhance nucleation and filling effects, excessively fine particles (especially at high dosages) are prone to agglomeration, which reduces their effectiveness in refining pore structure and controlling shrinkage. The surface of ultrafine limestone is covered with submicron-sized particles, providing a large number of active hydration sites, whereas the surface of coarse limestone has few surface attachments and thus offers weak hydration promotion capabilities [166]. From an engineering perspective, using limestone alone makes it difficult to balance accelerated early-age hydration with long-term dimensional stability. Relevant studies indicate that blending limestone with calcined clay can offset the increase in early-age shrinkage caused by ultrafine powders, thereby achieving a synergistic balance between strength and shrinkage [167]. As shown in Figure 26, the microstructure of limestone powder consists primarily of irregular, block-like particles. The particle size distribution of limestone powder with a particle size of 5 μm is uniform, and a large amount of fine powder (<1 μm) adheres to the surfaces of larger particles; this fine powder helps enhance both the nucleation and filling effects. In contrast, 20-μm limestone powder particles are coarse and have distinct edges, with relatively little fine powder adhering to their surfaces; consequently, their ability to refine pores and promote early hydration is significantly weaker. This particle size distribution allows it to better perform the three functions of nucleation, filling, and dilution in cement-based systems. Fine particles serve as heterogeneous nucleation sites for hydration products, accelerating the early hydration process [168]. While particles of varying sizes optimize the bulk density of the system and refine the pore structure, ultimately exhibiting distinctly different effects on autogenous shrinkage control at different dosages.

### 4.5. Metakaolin

**Figure 27:**
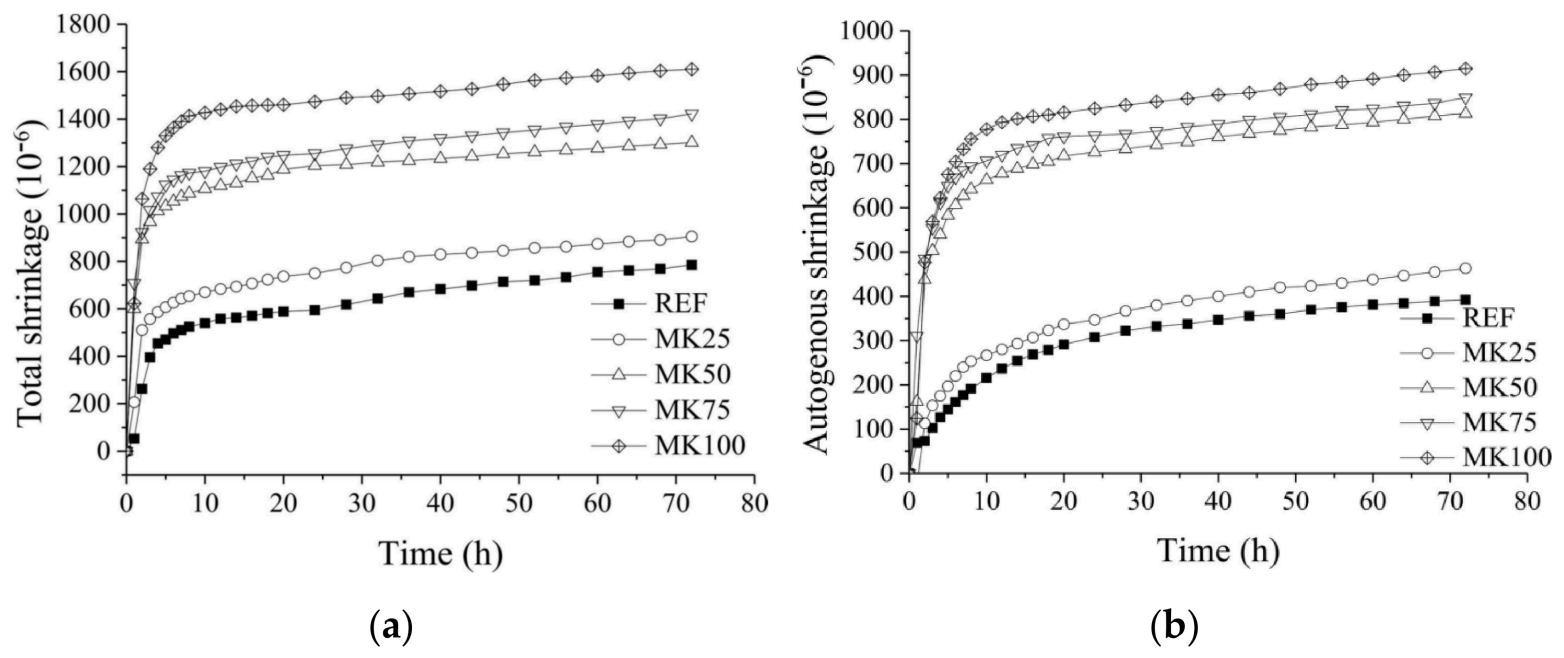
Shrinkage evolution of HPC pastes with different MK contents [169]: (**a**) Total shrinkage; (**b**) Autogenous shrinkage.

Modified kaolin (MK), a highly reactive mineral admixture, retains the lamellar structure of native kaolin to a certain extent. This material demands a higher water content to maintain a given slump, and its regulatory effect on HPC autogenous shrinkage depends primarily on the w/b and its own dosage. As shown in Figure 27, MK significantly increases the early-age (within 24 h) autogenous shrinkage of cement paste, with the increment becoming more pronounced as the MK content rises. During the period of 24 h to 72 h, the autogenous shrinkage of MK-blended groups remains higher than that of the reference group [169]. The evolution law of total shrinkage is consistent with that of autogenous shrinkage, the addition of MK markedly elevates the early-age total shrinkage, and a higher MK dosage corresponds to a greater increase in shrinkage. Gleize et al. [170] found through paste tests that the long-term autogenous shrinkage of MK decreases significantly with increasing dosage, and this reduction is primarily due to its pozzolanic effect rather than simple dilution. In systems with low w/b, MK initially accelerates hydration due to heterogeneous nucleation, causing a slight increase in autogenous shrinkages, but shrinkage decreases markedly in the later stages, which is distinctly different from the characteristic of silica fume, which continuously increases autogenous shrinkages. A comparative study by Weng et al. [171] indicated that, in contrast to fly ash, MK significantly increases the autogenous shrinkages of HPC. This is attributed to MK’s high specific surface area, rapid hydration water consumption, and refined pore structure, which intensify the degree of internal self-drying. In contrast, Salimi et al. [172] conducted experiments using low-reactivity MK and found that its effect on autogenous shrinkage is highly dependent on the w/b: at low w/b, increasing the dosage increases shrinkage, whereas at high w/b, it reduces autogenous shrinkage.

In summary, the extent to which MK affects the autogenous shrinkage of HPC depends primarily on the dosage, w/b, and MK activity. In HPC with a low w/b, MK significantly increases medium- to long-term autogenous shrinkage. Therefore, it must be used in conjunction with internal curing or composite admixtures to control the risk of cracking.

### 4.6. Other Mineral Admixtures

In addition to the five typical SCMs mentioned above, novel SCMs such as nano-silica (NS), rice husk ash (RHA), and calcined clay (CC) have also been applied in HPC systems. The effect of these admixtures on regulating HPC autogenous shrinkage exhibits significant variability depending on factors such as their quality (specific surface area, fineness, and reactivity), dosage, and w/b.

#### 4.6.1. Nano-Silica

NS is a highly reactive nano-scale admixture. Similar to silica fume discussed earlier, it primarily exerts a significant positive effect on the autogenous shrinkage of HPC. This effect is strongly dependent on dosage and is characterized by early-age behavior, making it one of the most effective types of SCMs in terms of autogenous shrinkage. Leveraging the high pozzolanic activity and filling effect of its nanoscale particles, NS significantly accelerates cement hydration and C-S-H gel formation, rapidly consumes free water, and intensifies the self-drying effect. Simultaneously, it drastically refines the capillary pore structure, leading to an exponential increase in capillary negative pressure. This ultimately results in a substantial increase in autogenous shrinkage. Zhang et al. [173] investigated the role of NS in a system with a w/b of 0.20, finding that at 2% and 4% dosages, 3-day autogenous shrinkage increased by 23.5% and 38.7%, respectively, while 7-day autogenous shrinkage increased by 31.2% and 49.5%. Due to its high pozzolanic activity, excellent filling effect, and the unique properties of its nanoparticles, NS can effectively improve the density, strength, and durability of concrete.

However, the large-scale application of nano-silica in engineering practice still faces several key challenges. First, dispersion and agglomeration issues represent the primary technical bottlenecks [174,175,176]. While the initial particle size of nanoscale silica ranges from 1 to 100 nm, the particle size distribution of its agglomerates typically reaches 100 to 400 μm. Agglomeration of ultrafine particles not only severely hinders the full realization of their nucleation, filling, and pozzolanic effects, but the C-S-H gel formed by the reaction of agglomerates with the hydration product calcium hydroxide may also result in localized defects due to uneven distribution, which can even have a negative impact on strength development. Studies have shown that the agglomeration effect of nano-silica becomes more pronounced as the dosage increases. When incorporated at 1%, the compressive strength of UHPC under standard curing conditions may even be slightly lower than that of the blank group, which is attributed to inadequate compaction caused by reduced workability [177]. Therefore, in practical applications, technical measures such as ultrasonic dispersion or surface modification are typically required to improve dispersion. Second, production costs and large-scale production capacity also pose significant constraints. Table 2 compares the characteristics of different nano-silica preparation processes. Although the Fumed method can produce high-purity products, its high energy consumption and substantial equipment investment limit its large-scale engineering applications. The precipitation method uses sodium silicate as the primary raw material, with a reaction temperature of only about 70 °C, the process is simple and raw material costs are low, accounting for over 90% of the domestic nano-silica market. However, its inherent drawbacks are significant: the synthesis process tends to form micron-sized particle agglomerates that are difficult to completely disperse when added to conventional concrete mixing systems. This not only undermines the advantage of pore refinement but also introduces microscopic defects into the matrix. Additionally, particle size control is imprecise, resulting in a clear upper limit on the performance of the final product [178]. The sol-gel method can precisely control particle size within the 20–200 nm range and enable surface functionalization, resulting in high-purity products. However, organic precursors such as ethyl orthosilicate are expensive, and the reaction system is highly sensitive to temperature and pH, making industrial-scale production extremely difficult. At present, it is used only for the preparation of small laboratory samples. In recent years, green biosynthetic routes using agricultural and forestry solid waste, such as rice husk ash, as raw materials have attracted widespread attention. This process facilitates the resource utilization of solid waste, it is low-carbon and environmentally friendly, with widely available raw materials. Under optimized conditions, it can produce nano-silica powder with a purity of ≥99%. However, due to constraints imposed by raw material impurities and the acid-base precipitation system, the adjustable particle size range is narrow. Under optimal conditions, the particle size can reach 276 nm. A complete, standardized mass production process has not yet matured, and there is significant batch-to-batch variation in particle size and purity. the project has a long return on investment cycle, and large-scale production processes still require further development and refinement [179]. These limitations dictate that the dosage of nano-silica in practical engineering applications should generally be kept at a low level and is often required to be blended with auxiliary cementitious materials such as fly ash and slag to balance its combined effects on hydration, shrinkage, and cost.

#### 4.6.2. Rice Husk Ash

RHA is a typical admixture derived from the resource utilization of agricultural waste. It is a biomass mineral admixture composed of nanoscale SiO_2_ particles and possesses high pozzolanic activity. Its primary effect on HPC is to inhibit autogenous shrinkage, and its regulatory effect is closely related to calcination temperature, fineness (D50), dosage, and w/b. It is a highly effective shrinkage-inhibiting admixture that combines pozzolanic activity with physical filling properties. RHA calcined at 700 °C exhibits optimal reactivity. When used at a dosage of 20% with a w/b of 0.30, it reduces 7-day autogenous shrinkage by 24.5% [180]. The shrinkage-inhibiting effect of RHA is more pronounced in systems with low w/b. In a study by Ye et al. [181], the dosage of 25% at a w/b of 0.22 reduced 28-day autogenous shrinkage by 36.8%. The shrinkage-inhibiting effect is maximized in high w/b systems, at a w/b of 0.35, the dosage of 30% RHA reduced 28-day autogenous shrinkage by as much as 62.4% [182], making it one of the most effective types of SCMs for shrinkage control. In engineering applications, RHA of different fineness can be selected based on the w/b: fine-grained ash for low w/b systems and coarse-grained ash for high w/b systems, with the dosage controlled between 10% and 30%.

#### 4.6.3. Calcined Clay

**Figure 28:**
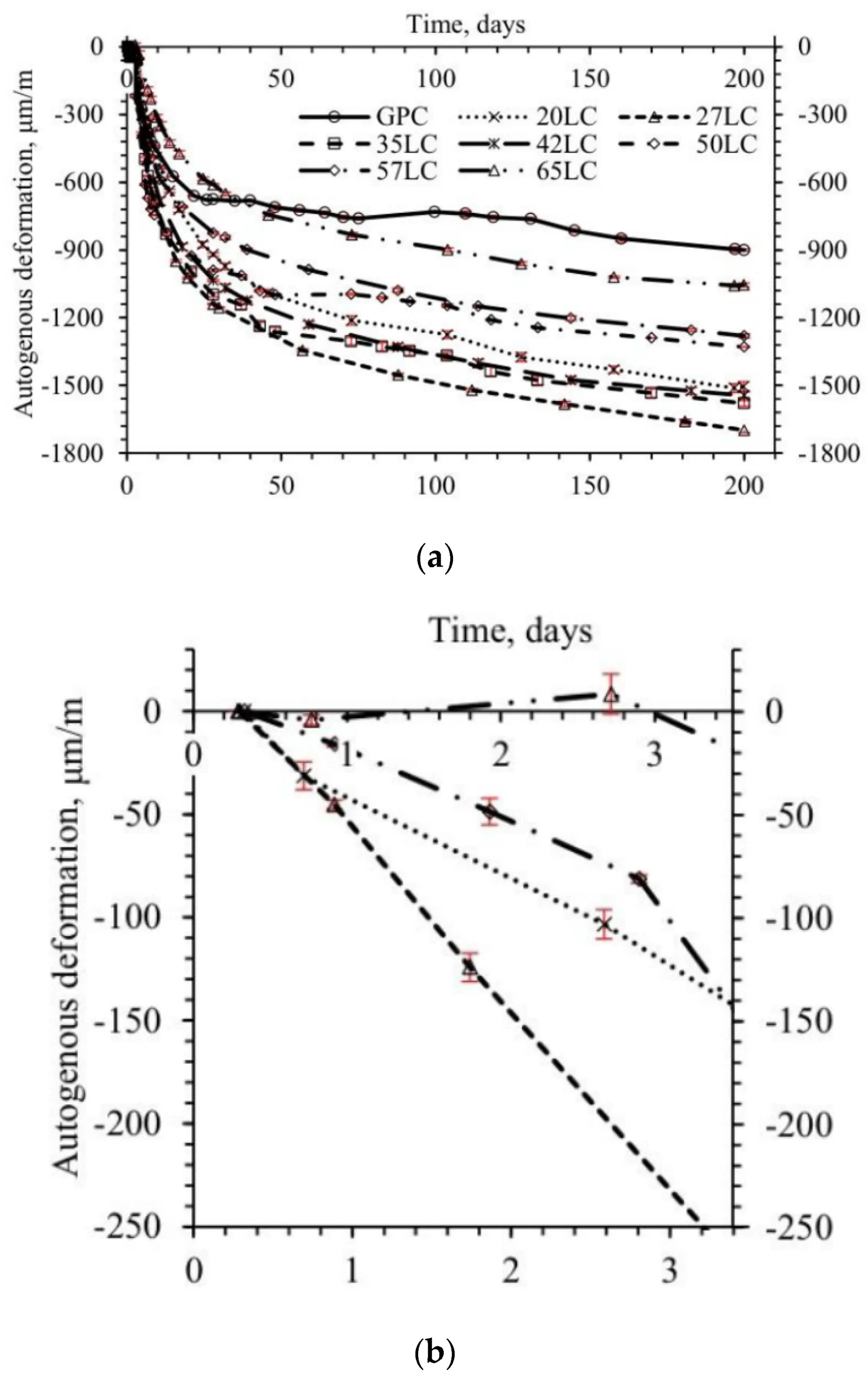
Autogenous shrinkage of paste samples with error bars (**a**) up to 200 days and (**b**) up to 3.5 days (20LC, 27LC, 57LC and 65LC) [183].

CC, primarily consisting of limestone-composite CC and highly reactive CC, exhibits a significant inhibitory effect on the autogenous shrinkage of HPC. It is an important type of green, low-carbon admixture, and its shrinkage-suppressing effect stems from the synergistic action of physical filling and pozzolanic reactions. As shown in Figure 28, the CC-limestone composite admixture can significantly modulate the autogenous shrinkage behavior of HPC. As the dosage increases, the system’s autogenous shrinkage generally follows a pattern of first increasing and then decreasing. Within a low dosage range, autogenous shrinkage increases slightly [183]. However, as the dosage is further increased, the physical dilution effect becomes dominant, significantly suppressing autogenous shrinkage. Studies have shown that for CC-limestone composites at a w/b of 0.30, the dosage of admixture increased from 10% to 20%, resulting in a rise in the 7-day autogenous shrinkage reduction from 15.6% to 28.3% [184]. For highly active calcined clay at a w/b of 0.28, the dosage of admixture was increased from 15% to 25%, resulting in a rise in the 28-day autogenous shrinkage reduction from 21.7% to 35.9% [185]. Calcined clay exhibits a mild pozzolanic reaction rate. The resulting low-calcium-to-silica C-S-H gel effectively densifies the matrix and optimizes the capillary pore structure, while the inert diluting effect of limestone delays early hydration. The synergistic effect of these two mechanisms achieves the dual objectives of autogenous shrinkage suppression and durability enhancement. This makes it an excellent alternative to fly ash and, suitable for promotion and application in green HPC systems, with a recommended dosage of 10–25%.

The above studies indicate that different pozzolanic admixtures have significantly different effects on controlling the autogenous shrinkage of HPC [186]. The above studies indicate that different volcanic ash-based pozzolanic materials exhibit significant differences in their ability to control the autogenous shrinkage of HPC. Table 3 provides a comprehensive comparison of the resource availability, price, effects on autogenous shrinkage, and engineering applicability of the eight SCMs discussed in this paper. Fly ash and rice husk ash are widely available, low-cost materials with broad engineering applicability; they can effectively suppress HPC autogenous shrinkage and reduce the risk of early-age cracking. Blast furnace slag, a solid waste product of the steel industry, has a stable supply and is cost-effective; however, it slightly increases the early-age autogenous shrinkage of concrete, with the degree of shrinkage increasing as the dosage and fineness increase. Kaolinite has limited reserves and relatively high costs, it promotes autogenous shrinkage in the early stages, while its late-age shrinkage behavior is determined by both its own reactivity and the dosage. Silica fume and nano-silica have high fineness and excellent pozzolanic activity, significantly exacerbating the early-age autogenous shrinkage of HPC. Among these, silica fume has limited production and relatively high costs, and is mostly used in the preparation of ultra-high-strength concrete. Nano-silica has a complex production process and extremely high costs, and is mostly used in special applications. Limestone powder and calcined clay are moderately priced, both possess unique bidirectional regulatory properties. At low dosages, they slightly increase autogenous shrinkage, while at high dosages, they can suppress shrinkage through a combination of dilution effects and synergistic interactions, making them preferred materials for green, low-carbon concrete. In addition, when selecting SCMs for actual engineering projects, mechanical properties, durability, and environmental benefits must be comprehensively considered. While some low-cost solid waste-based admixtures can improve volumetric stability and facilitate the resource utilization of solid waste, they often suffer from issues such as slow early-age strength development and reduced carbonation resistance at high dosages. Highly reactive admixtures can optimize the mechanical properties of the matrix but are accompanied by limitations such as increased autogenous shrinkage and prohibitively high costs that hinder large-scale application. When selecting materials to meet low-carbon goals, it is equally important to balance the long-term durability performance of structural members and achieve a trade-off among multiple objectives [187,188].

## 5. Influence of Compound SCMs on Autogenous Shrinkage of HPC

The use of single SCMs to control the autogenous shrinkage of HPC has significant limitations. For example, the use of highly reactive admixtures alone tends to increase early-age autogenous shrinkage, while the use of low-reactivity admixtures alone may result in delayed early-age strength development. In contrast, composite admixture systems achieve multi-objective optimizations suppressing autogenous shrinkage while enhancing mechanical properties and durability—through the complementary properties and synergistic effects of different admixtures. This approach represents the mainstream technical strategy for controlling HPC autogenous shrinkage in engineering applications [189,190]. Essentially, this approach utilizes the diluting and retarding effects of low-reactivity admixtures (such as fly ash and limestone powder) to suppress early-age self-drying and the development of capillary negative pressure. Simultaneously, it leverages the pozzolanic reactions and filling effects of high-reactivity admixtures (such as silica fume, ultrafine slag, and metakaolin) to optimize the microporous structure and enhance matrix density. The synergistic action of these two types of admixtures offsets the negative effects of using a single admixture, thereby achieving balanced control over autogenous shrinkage and mechanical properties. Current mainstream composite admixture systems can be classified into binary and multi-component systems. Binary admixtures, characterized by simple mixing ratios and clear effects, form the foundation of engineering applications, while multi-component admixtures (primarily ternary systems) are more suitable for specialized engineering projects with stringent requirements for shrinkage and comprehensive performance, such as ultra-high-performance concrete (UHPC) and marine HPC [190].

### 5.1. The Effect of Binary Blending Systems on the Autogenous Shrinkage of HPC

Binary blending is the most established engineering technique for controlling the autogenous shrinkage of HPC using SCMs. Its core principle lies in leveraging the complementary properties and synergistic effects of shrinkage-inhibiting, low-reactivity admixtures and strength-enhancing, high-reactivity admixtures, to address the inherent shortcomings of single-admixture systems. This approach not only offsets the early-age expansion and shrinkage issues caused by the sole use of highly reactive admixtures such as silica fume and NS but also compensates for the delayed early-age strength associated with the sole use of low-reactivity admixtures such as fly ash and limestone powder. Ultimately, it achieves a multi-objective balance of autogenous shrinkage suppression, enhanced mechanical properties, and optimized durability [191,192,193,194,195]. The core of this regulation follows the principle of “early shrinkage suppression and late strength enhancement”: by utilizing the dilution, retarding, and ball-bearing effects of fly ash and low-surface-area limestone powder, the early hydration rate of cement is delayed, free water consumption is reduced, and the early development of self-drying and capillary negative pressure is suppressed. By leveraging the high pozzolanic activity and microfiller effects of silica fume, ultrafine slag, and metakaolin, the microstructural porosity is optimized and matrix density is enhanced, ensuring the long-term strength and durability of HPC. Furthermore, the overall regulatory effect is significantly influenced by the admixture combination, proportioning ratios, total admixture dosage, and w/b. Research indicates [73,196,197], the binary blending of fly ash and silica fume can effectively suppress the significant increase in HPC autogenous shrinkage caused by the sole addition of silica fume, while also effectively compensating for the delayed early-age mechanical strength development associated with the sole addition of fly ash. Within a reasonably optimized dosage range, this can effectively reduce HPC autogenous shrinkage by 20–40%. Lei et al. [198] investigated self-compacting UHPC with a hybrid blend of fly ash and silica fume. Under the optimal mix ratio (silica fume:fly ash = 0.15:0.39), the composite system reduced matrix porosity by 28.26%, improved the interfacial transition zone microstructure between aggregates and paste, and decreased 28-day autogenous shrinkage by approximately 35% compared to the silica-fume-only reference group. The dual-blend of fly ash and slag balances the contraction-increasing effect of slag with the contraction-reducing effect of fly ash, achieving synergistic optimization of shrinkage and mechanical properties. As demonstrated by Wang et al. [199], in a system containing 20% Grade I fly ash + 20% slag (380 m^2^/kg) (total dosage 40%) with a w/b of 0.35, the 7-day, 28-day, and 90-day autogenous shrinkage were reduced by 32.6%, 25.8%, and 18. 7%, respectively, compared to the control group. Zhao et al. [131] blended 740 m^2^/kg of ultrafine slag with Grade I fly ash in equal proportions (25% each), at a w/b of 0.30, the reduction in autogenous shrinkage at 7 days and 28 days reached 35.2% and 24.6%, respectively. The total dosage of admixture for this system should be controlled between 30% and 50%, with a recommended fly ash to slag ratio of 1:1 to 2:1. This formulation is suitable for engineering applications requiring low shrinkage, low hydration heat, and high durability. The slag-silica fume blended system is suitable for UHPC with an ultra-low w/b (≤0.25). Slag possesses pozzolanic activity, which increases autogenous shrinkage in systems with low w/b. However, its relatively slow hydration rate can delay the rapid early-stage hydration of cement through a dilution effect. Silica fume, on the other hand, significantly enhances early strength due to its extremely high reactivity and filling effect, but it also exacerbates autogenous shrinkage during the early stages. The blended use of the two materials creates a complementary regulatory effect: the slow hydration of slag mitigates the excessive consumption of free water in the system and stabilizes the abrupt rise in capillary pressure, thereby offsetting the severe early-age expansion and shrinkage effects caused by the sole use of silica fume. While silica fume continuously stimulates the pozzolanic reaction in the later stages, generating large amounts of C-S-H gel to densify the pore structure, ensuring that the UHPC achieves an ultra-high strength of ≥120 MPa. Following co-blending, the system’s autogenous shrinkage displays a two-stage pattern: low-amplitude growth during the early stage, followed by stable development in the later stage. Li et al. [149] investigated a UHPC system containing 40% slag (380 m^2^/kg) and 6% silica fume, with a w/b of 0.20. They found that the 7-day and 28-day autogenous shrinkage of this system was reduced by 8.5% and 6.2%, respectively, compared to a system containing only 6% silica fume. They also tested a dual-blend system containing 30% slag (420 m^2^/kg) and 8% silica fume. At a w/b of 0.25, the 7-day and 28-day autogenous shrinkage in this system increased only slightly by 4.8% and 2.1%, respectively, compared to the control group. The silica fume dosage in this system must be strictly controlled between 5% and 8%. The slag dosage is 30–40%, making it suitable for ultra-low w/b applications such as UHPC bridge decks and precast components. The dual blending of fly ash and limestone powder is a typical binary system for green, low-carbon HPC. Limestone powder, with its low specific surface area, acts as an inert admixture that reduces clinker consumption and refines the pore structure through physical filling effects. Together with fly ash, it creates a dual regulatory mechanism of “physical shrinkage suppression and chemical retarding.” Since there is no competition for hydration between the two, their regulatory effects exhibit an additive nature. Xiong et al. [200] studied a system comprising 15% ordinary limestone powder and 15% Class I fly ash, at a w/b of 0.34, the 90-day autogenous shrinkage was reduced by 14. 8% compared to the reference group. However, this system is selective regarding the combination of admixtures. Canda et al. [188] studied a composite system of 20% limestone powder and 10% CC, at a w/b of 0.32, the 7-day and 28-day autogenous shrinkage increased by 8.7% and 6.5%, respectively, compared to the control group. Therefore, in engineering applications, low-reactivity limestone powder with a specific surface area ≤ 400 m^2^/kg should be selected, with the total dosage controlled between 20% and 35%, making it suitable for green concrete projects such as municipal roads and residential buildings. The composite admixture of fly ash and RHA constitutes a highly effective shrinkage-suppressing binary system, RHA calcined at 700 °C exhibits optimal reactivity, where the dilution effect of coarse-grained RHA significantly delays early-stage hydration, while the filling effect of fine-grained RHA refines the pore structure. This synergizes with the retarding and shrinkage-suppressing effects of fly ash. Furthermore, the pozzolanic reaction rate of RHA is mild, avoiding hydration competition, and in the later stages, it jointly forms a dense C-S-H gel matrix. Cheng et al. [181] investigated a composite system of 30% RHA calcined at 700 °C and 20% fly ash, at a w/b of 0.33, the 7-day and 28-day autogenous shrinkage values were reduced by 62.4% and 48.0%, respectively, compared to the control group. In engineering applications, coarse-grained RHA (D50 ≥ 20 μm) calcined at 700 °C should be prioritized, with the total dosage controlled between 30% and 50%. This is suitable for HPC projects with low w/b (0.25–0.33) and green building material projects utilizing agricultural waste. Table 4 shows the effect of different binary admixture systems on the autogenous shrinkage of HPC.

Overall, the autogenous shrinkage control performance of a binary blended admixture system is significantly influenced by the compatibility of the admixtures’ reactivity and their mixing ratios. an excessively high proportion of low-activity shrinkage-inhibiting admixtures tends to result in delayed strength development, while an excessively high proportion of high-activity strength-enhancing admixtures tends to cause early-age expansion and shrinkage. In engineering applications, the optimal admixture combination and proportioning parameters must be determined by considering the concrete strength class, w/b, and service environment to achieve synergistic optimization of autogenous shrinkage, mechanical properties, and durability.

### 5.2. Effect of Multi-Component Blending Systems on the Autogenous Shrinkage of HPC

In a ternary composite admixture system, Zhao et al. [131] found that the 7-day autogenous shrinkage of HPC with a composite admixture of 20% fly ash, 20% slag, and 10% limestone powder was reduced by approximately 38.2% compared to concrete with a dual admixture of 20% fly ash and 20% slag. This is primarily because the inert diluting effect of limestone powder reduces the early-stage hydration rate of the paste, slows the rate of free water consumption, and delays the decline in the pore IRH. At the same time, the physical filling effect of limestone powder optimizes the pore structure after the co-blending of fly ash and slag, reducing the intensity of capillary pressure and achieving a synergistic effect between the hydration rate and pore structure optimization. Sakthivel et al. [201] demonstrated that concrete made from a ternary composite cementitious system—a blend of fly ash, slag, and silica fume—exhibits significantly lower long-term shrinkage compared to pure cement concrete, with the shrinkage reduction effect of the silica fume-slag blend being superior to that of the fly ash-slag blend. This is primarily because the high pozzolanic activity and ultrafine filling effect of silica fume can significantly refine the pore structure of the paste, while slag supplements hydration products and enhances the density of the system. The synergistic action of these two components reduces the rate of decline in IRH and capillary negative pressure. Simultaneously, the ternary admixture system delays the early hydration process, reduces the consumption of free water, and further suppresses shrinkage deformation caused by self-drying, thereby achieving the dual effects of hydration control and pore structure optimization. Through the combined effects of shrinkage suppression by low-activity admixtures, strength enhancement by high-activity admixtures, and synergistic effects of functional admixtures, ternary admixture systems overcome the limitations of binary systems in balancing shrinkage control and mechanical properties. They are particularly suitable for engineering applications with stringent requirements for shrinkage behavior, mechanical strength, and durability, such as ultra-high-performance concrete (UHPC), mass concrete, and marine concrete [197,202]. The following recommendations are provided for different engineering applications:

(1) High-strength/ultra-high-strength HPC (C80–C100): The “fly ash-slag-silica fume” ternary admixture system is the preferred choice, with a ratio of fly ash:slag:silica fume = 4:3:1–5:3:1 and a total dosage of 40–50%, to achieve both low shrinkage and high strength.
(2) Green, low-carbon HPC: Prioritize the use of the “fly ash-slag-limestone powder” ternary blended system, with a ratio of fly ash:slag:limestone powder = 3:3:2–4:3:2 and a total dosage of 45–65%, to reduce cement consumption and carbon emissions.
(3) Ultra-low w/b UHPC (w/b ≤ 0.20): The “fly ash-slag-silica fume-expansion agent” four-component blending system is recommended, with a ratio of fly ash:slag:silica fume:expansion agent = 5:4:1:1 and a total dosage of 60–70%, to achieve significant reduction in autogenous shrinkage and ultra-high strength.
(4) Mass-produced/offshore HPC: Prioritize the “fly ash-slag-limestone powder” or “slag-limestone powder-MK” ternary admixture system, with a total dosage of 50–60%, to balance low shrinkage, low hydration heat, and high durability.

## 6. HPC Autogenous Shrinkage Prediction Model

Autogenous shrinkage prediction models are core tools for precisely controlling the shrinkage behavior of HPC and for early warning of cracking risks in engineering structures. With the widespread application of SCMs in HPC, their complex regulatory effects on the hydration process, pore structure, and water transport have made traditional prediction models based on pure cement systems insufficient to meet engineering accuracy requirements. In recent years, the academic community has developed various prediction methods—including empirical regression models, mechanism-based models, and numerical simulation models—focused on the influence mechanisms of SCMs. These methods have progressively enabled the quantitative characterization of autogenous shrinkage behavior in systems with single, multiple, and even specialized admixtures. This section systematically reviews the construction principles, applicable scenarios, and limitations of mainstream prediction models, focusing on model innovations that account for the coupling effects of SCMs, and supplements these with the latest research findings and supporting literature.

### 6.1. Empirical Regression Models

These models rely on extensive experimental data to establish, through statistical analysis, a multiple regression relationship between autogenous shrinkage and key factors such as mineral admixture type, dosage, and w/b. They offer the advantages of a simple form and convenient computation. A typical early representative is the Tazawa & Miyazawa model. Its core expression, shown in Equation (3), introduces a cementitious material composition parameter (α) and a hydration degree correlation coefficient (β), thereby achieving, for the first time, a preliminary quantification of the influence of admixtures such as slag and fly ash:

(3) $$\begin{array}{c} \varepsilon_{\mathrm{as}}(t)=\alpha \cdot \ln(1+\beta t) \end{array}$$

where: *ε_as_*(*t*): autogenous shrinkage strain at age *t*; α: coefficient related to the composition of cementitious materials (including the type and content of SCMs); *β*: coefficient related to hydration rate; *t*: curing age (d).

**Figure 29:**
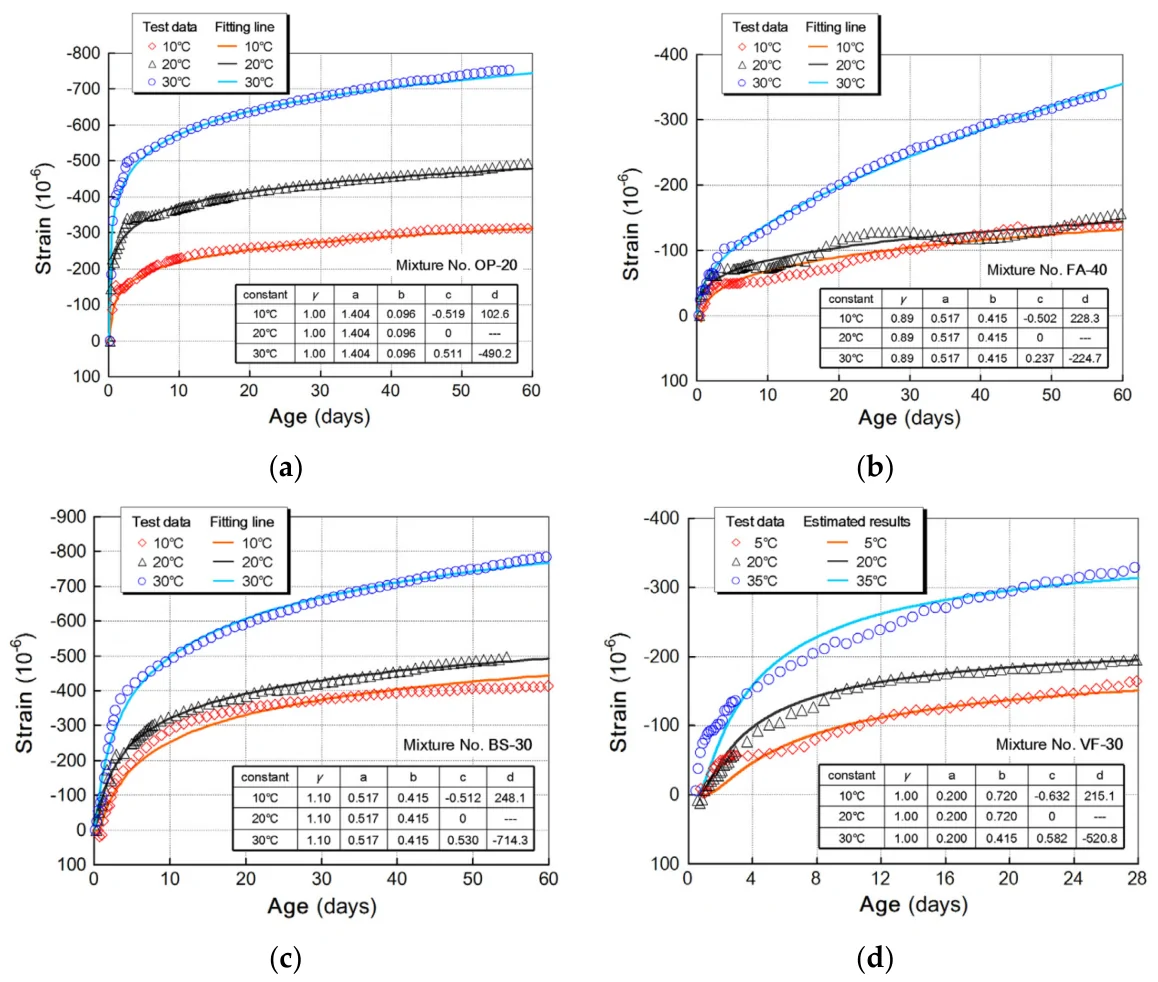
Estimation of autogenous shrinkage by a modification on a previous model: (**a**) OP-20, (**b**) FA-40 and (**c**) BS-30 mixture; (**d**) the VF-30 mixture is used to verify the present model [73].

Subsequent researchers conducted multidimensional optimizations to address the limitations of this model: Jiang et al. [73] used nine sets of HPC test data to correct the autogenous shrinkage prediction model for the effects of temperature and admixtures, and the fitted curve showed a high degree of agreement with the measured data (Figure 29), verifying the model’s applicability for water-to-cement ratios of 0.20–0.40, various SCMs systems, and temperature conditions ranging from 10–30 °C, providing experimental support and a modeling basis for subsequent multifactorial coupled prediction of HPC autogenous shrinkage.

Miao et al. [203], addressing the characteristics of composite additions of limestone powder and slag in coarse aggregate ultra-high-performance concrete (CA-UHPC), made targeted modifications to the GL2000 empirical model [204] to establish autogenous shrinkage prediction equations (Equations (4)–(7)) better suited for UHPC containing SCMs and coarse aggregates. This model retains the core framework of GL 2000, introduces a coefficient reflecting the influence of mineral admixture dosage, and divides the time function β(t) into two segments based on 28 days:

(4) $$\begin{array}{c} \varepsilon_{\exp}=\varepsilon_{shu}\beta (RH)\beta (t) \end{array}$$

(5) $$\begin{array}{c} \varepsilon_{shu}=1000K{\left(\frac{\alpha }{f_{\mathrm{cm}28}}\right)}^{0.5}\cdot 10^{-6} \end{array}$$

(6) $$\begin{array}{c} \beta (RH)=\left(1-1.18RH^{4}\right) \end{array}$$

(7) $$\begin{array}{c} \beta (t)=\left\{\begin{array}{c} 1-e^{\beta (t-t_{c})}\;0<t<28\\ 1.4\times {\left[\frac{t-t_{c}}{t-t_{c}+(0.15V/S{)}^{2}}\right]}^{0.5}t>28 \end{array}\right. \end{array}$$

where: *ε*_exp_: experimental autogenous shrinkage of CA-UHPC; t: age of concrete; RH: humidity; tc: age of concrete at start of drying shrinkage; K: coefficient of cement type; V/S: volume:surface ratio; f_cm_: concrete mean compressive strength at 28 d; *ε*_shu_: autogenous shrinkage coefficient; α = 55; β = 0.15.

**Figure 30:**
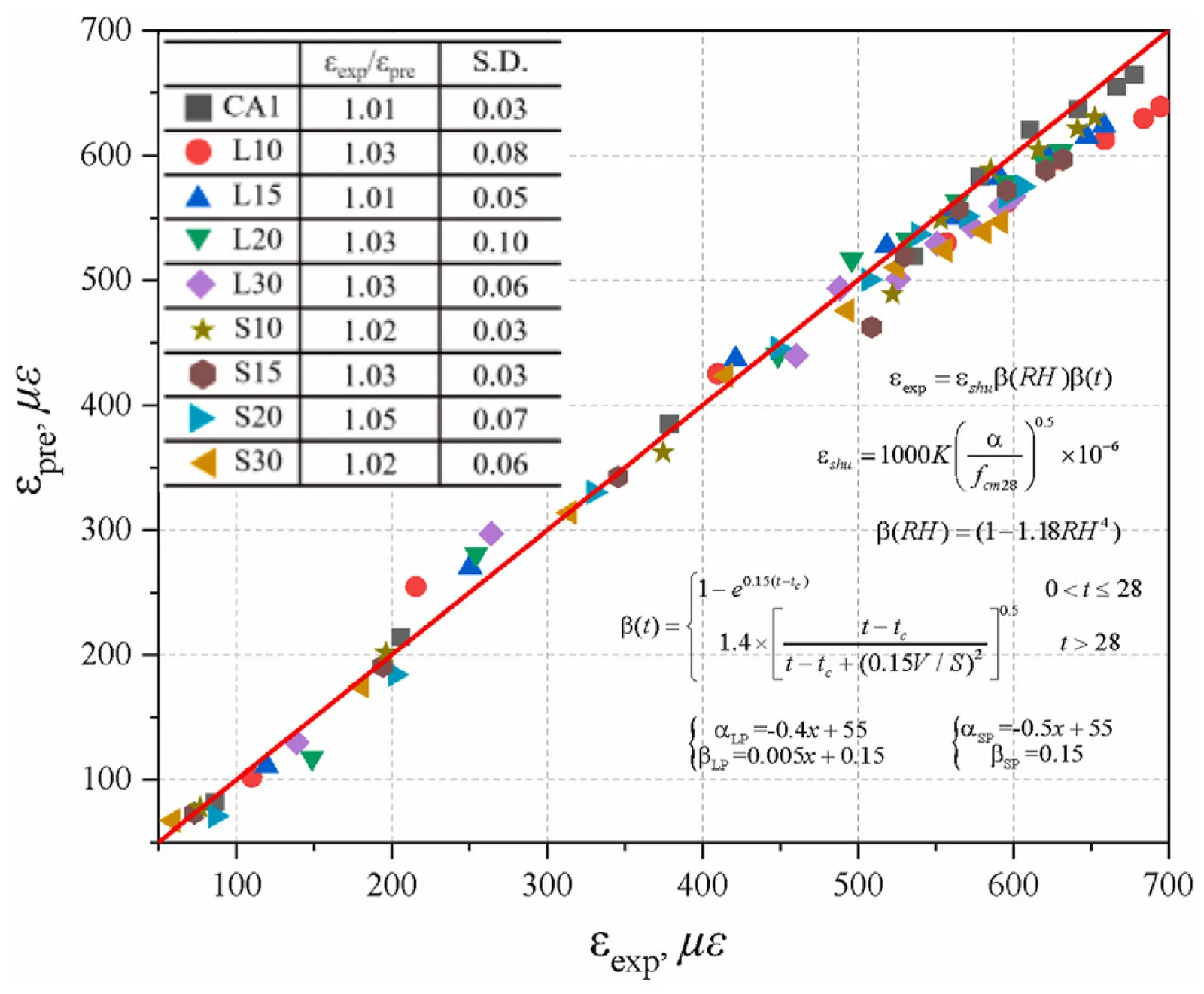
Comparison between the autogenous shrinkage strain values of CA-UHPC and the calculated values of modified mode [203].

At the same time, the model provides a linear quantitative relationship between the dosage of limestone powder and slag and the model parameters α and β, which directly reflects the inhibitory effect of SCMs on the development of autogenous shrinkage. Validation results show (Figure 30) that the mean measured-to-predicted ratio of this modified model is 1.03, with a standard deviation of only 0.06. This significantly improves the predictive accuracy of traditional empirical models in systems with high-dose SCMs and UHPC containing coarse aggregates, addressing the shortcomings of conventional models that do not account for the coupling effects between coarse aggregate constraints and SCMs.

### 6.2. Equivalent Age Models

This class of models is based on the Arrhenius temperature effect principle. By converting the influence of SCMs on the hydration process into an equivalent age, it modifies the traditional time function and is particularly suitable for complex conditions such as steam curing and variable-temperature curing. A typical example is the modified autogenous shrinkage model for steam-cured UHPC, based on the Tazawa & Miyazawa model [205]. Its core concept is to integrate the coupled effects of temperature and SCMs through equivalent time (teq), as shown in Equation (8):

(8) $$\begin{array}{c} t_{\mathrm{eq}}=\int_{0}^{t}\exp\left(-\frac{\mathrm{Ea}}{R}\left(\frac{1}{T(t)}-\frac{1}{T_{0}}\right)\right)\cdot dt \end{array}$$

where: *Ea*: Activation energy of hydration reaction (J/mol), the value for mineral admixture systems is 40 kJ/mol; *R*: Gas constant (8. 314 J/(mol·K)); *T*(*t*): Instantaneous curing temperature (K); *T*_0_: Reference temperature (293 K, i.e., 20 °C).

**Figure 31:**
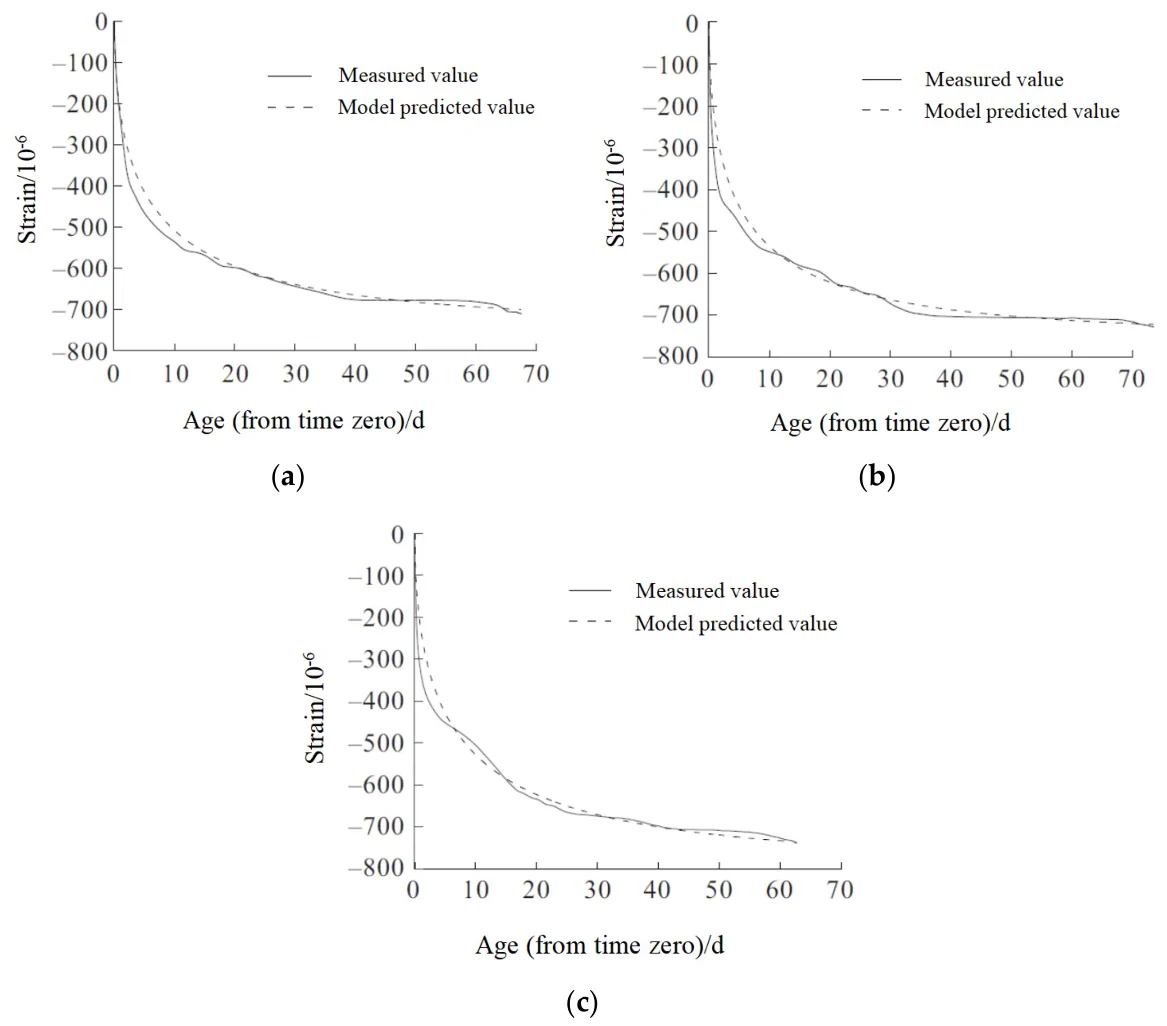
Measured value and predicted value of each specimen [205]: (**a**) F1 batch test piece; (**b**) F2 batch test piece; (**c**) F3 batch test piece.

To verify the accuracy of the established UHPC autogenous shrinkage prediction model, the researchers compared the measured autogenous shrinkage strains of three batches of specimens (F1, F2, and F3) with the model-predicted values. The results are shown in Figure 31. The figure uses time zero as the starting point and the equivalent curing age at 20 °C as the *x*-axis to compare the measured values with the model-calculated values. The results indicate that the model predictions for all three batches of specimens agree well with the measured values, with coefficients of determination (R^2^) all exceeding 0.98. This demonstrates that the model can accurately describe the autogenous shrinkage behavior of UHPC under steam curing conditions. Under equivalent 20 °C sealed curing conditions, the autogenous shrinkage of this UHPC is essentially complete after approximately 40 days.

### 6.3. Intelligent Prediction Model

**Figure 32:**
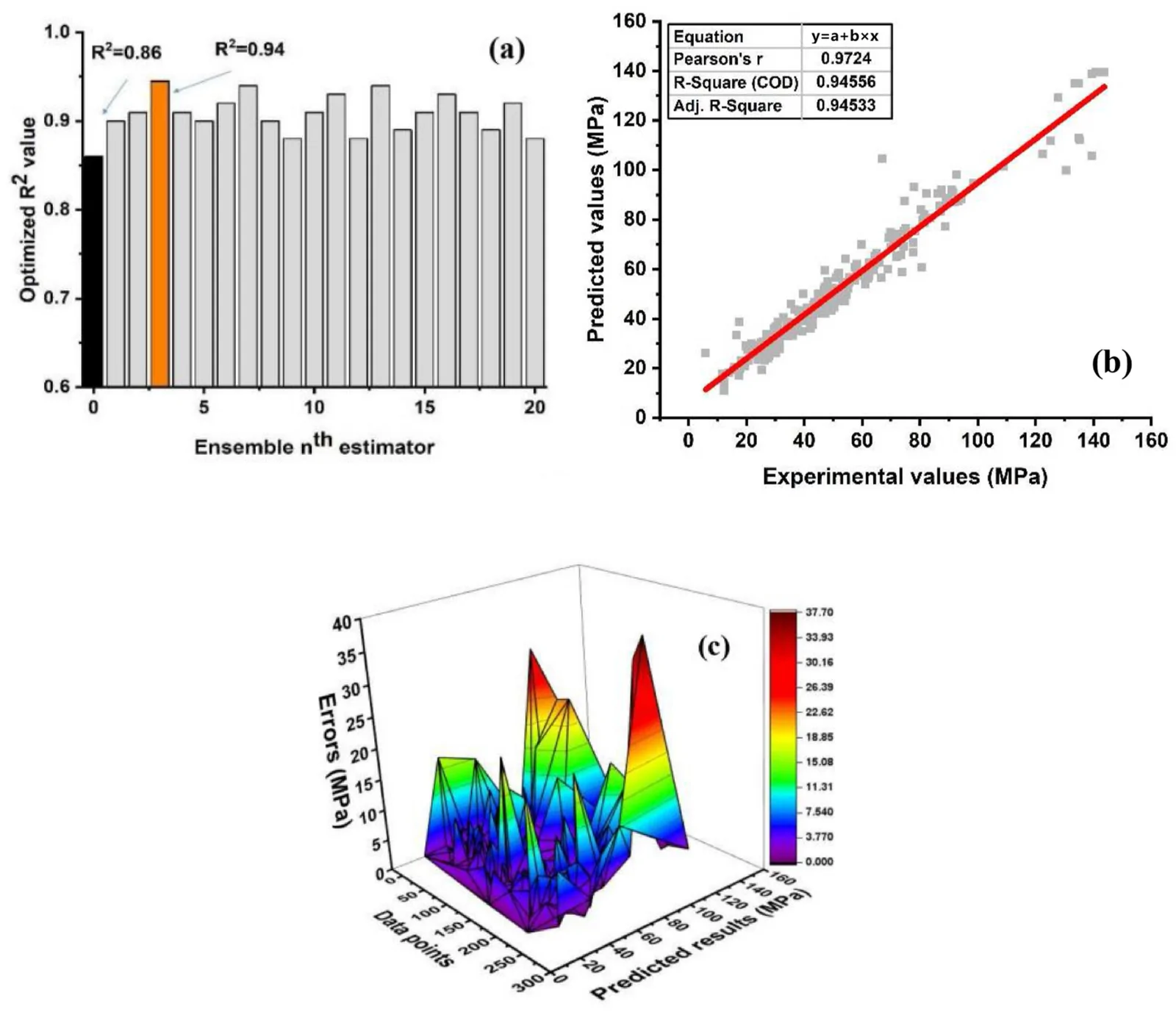
Results of RF model with nth Estimator: (**a**) results obtained from 20 sub-models; (**b**) regression graph of best performing model; (**c**) error distribution of the best estimator [179].

Traditional empirical and theoretical models are often based on predefined functional forms for fitting, making it difficult to efficiently handle nonlinear mapping relationships under multi-factor coupling. In recent years, data-driven methods, particularly machine learning, have been widely adopted in the field of concrete performance prediction due to their robust nonlinear fitting and generalization capabilities. Amin et al. [179] systematically developed both standalone and ensemble machine learning prediction models for HPC using RHA as a mineral admixture, aiming to accurately predict concrete compressive strength. In this study, using water, cement, coarse and fine aggregates, RHA, curing age, and the dosage of high-efficiency water-reducing agent as input parameters, basic predictive models such as decision trees (DT), random forest regression (RFR), and support vector regression (SVR) were established. Bagging and Boosting ensemble strategies were then introduced to optimize these base models. Through hyperparameter tuning and 10-fold cross-validation, the stability and predictive accuracy of the models were effectively improved. Among them, the Bagging ensemble model based on decision trees and random forests performed best, with a coefficient of determination (R^2^) greater than 0.80, while the Boosting ensemble model achieved an R^2^ greater than 0.90, significantly outperforming single machine learning models. To visually illustrate the model optimization process and prediction results, the accuracy of the Random Forest model’s submodels, the optimal fit, and the error distribution are presented collectively in Figure 32.

This type of intelligent predictive model does not require predefined mechanical or shrinkage development functions. It can adaptively fit the coupled effects of raw material parameters, mix proportions, and age on concrete performance, demonstrating excellent applicability in concrete systems with high-dosage agricultural solid waste admixtures. In addition to the studies mentioned above, machine learning has already established a relatively mature technical framework in the broader field of concrete performance prediction. In terms of programming environments, Python has become the undisputed mainstream development platform in this field, owing to its rich scientific computing libraries (such as scikit-learn, XGBoost, and LightGBM) and active open-source community. Researchers can leverage lightweight frameworks such as Streamlit or Flask to package trained models into engineering-ready interactive prediction tools, thereby bridging the gap between laboratory models and engineering applications. At the model architecture level, current research shows a trend toward evolving from single algorithms to ensemble learning and hybrid models. Gradient-boosting tree algorithms, represented by XGBoost, LightGBM, and CatBoost, perform exceptionally well in multi-class prediction tasks, with prediction accuracy and robustness generally superior to those of traditional backpropagation (BP) neural networks and support vector machines (SVMs). For example, in the prediction of early shrinkage in ultra-high-performance concrete (UHPC), the Bayesian-optimized XGBoost model (BO-XGBoost) demonstrated higher accuracy and stability compared to traditional BP neural networks and random forests [206]. In composite systems incorporating superabsorbent polymers (SAP) and SCMs, the coefficient of determination (R^2^) of the Gene Expression Programming (GEP) model can reach over 0.96 [207]. Furthermore, Random Forest and Support Vector Machines (SVM), as classical algorithms, are still widely used as benchmark models. In terms of deep feature extraction and time-series modeling, deep learning methods such as Long Short-Term Memory (LSTM) networks have also been explored to capture microstructural image sequences or temporal dependencies during the hydration process [208]. To balance accuracy and interpretability, the latest research trend involves constructing hybrid models that integrate physical mechanisms with data-driven approaches, embedding key parameters—such as hydration heat and internal relative humidity predicted by machine learning—into traditional constitutive models to achieve physically consistent autogenous shrinkage predictions [209]. However, it is worth noting that most of the aforementioned models are based on small laboratory datasets, more than 85% of published models have not yet been fully validated in real industrial settings, and their generalizability and engineering reliability still require further testing.

### 6.4. Limitations of Existing Models and Directions for Improvement

Shrinkage prediction models can accurately forecast the shrinkage deformation of concrete at specific ages and even in its final state. Not only do they significantly shorten the testing cycle and reduce testing costs, but the resulting predictions also provide a basis for structural equivalent analysis of components such as beams [210]. This is of great significance for research on the volumetric stability of concrete. This section analyzes and compares the six models reviewed earlier, as shown in Table 5.

As can be seen from the table, traditional empirical and equivalent-age models feature concise formulas and are convenient for engineering calculations. However, their parameters are primarily determined through experimental testing, lack uniform standard values, and do not fully account for the coupled effects of environmental factors such as curing temperature and humidity. Intelligent prediction models represent a modeling approach with greater development potential today, but they involve high modeling and computational complexity, and there is still a relative lack of field test data for validation at this stage. Furthermore, most existing autogenous shrinkage prediction models are based on pure cement or single-component SCMs, making it difficult to adapt them to the more complex multi-component blends encountered in engineering practice. First, the interactions between different SCMs in multi-component systems—such as the diluting effect of low-activity components inhibiting the accelerated early hydration of high-activity components, or the pozzolanic reaction of high-activity components altering the packing efficiency of low-activity component particles—exhibit nonlinear and non-additive characteristics that cannot be described by a single activity coefficient or dosage correction factor in existing models. Second, key parameters in mechanistic models—such as the chemical shrinkage coefficient, water diffusion coefficient, and pore structure evolution parameters—are often calibrated based on experimental data from single-component systems. When the system shifts to a multi-component blend, both the hydration kinetics and pore structure evolution pathways undergo significant changes, rendering the original parameter set no longer applicable.

Future model development should focus on three key directions: (1) A unified predictive framework that integrates multiple coupling factors, incorporating key parameters such as SCMs type, particle characteristics, composite admixture proportions, w/b, and curing conditions, should be established. This approach focuses on addressing the model’s limited applicability in multi-component composite admixture systems, thereby effectively enhancing the model’s generalizability. (2) Machine learning should be integrated with mechanistic derivation methods to optimize model parameters through big data training, thereby enhancing theoretical depth while ensuring computational efficiency. (3) Specialized models suitable for extreme environments (such as high temperatures, high humidity, and saline–alkali soils) should be developed to meet the shrinkage prediction requirements for HPC in specialized engineering projects.

## 7. Conclusions and Outlook

### 7.1. Conclusions

This paper systematically analyzes the types of HPC shrinkage and the mechanisms underlying its autogenous shrinkage. It examines the patterns and control mechanisms of autogenous shrinkage in HPC when using typical SCMs, both as single admixtures and in blends. This study quantitatively investigates the coupled effects of key factors such as admixture type and dosage, and reviews existing autogenous shrinkage prediction models. The core conclusions are as follows:

(1) HPC autogenous shrinkage results from the coupling of CS, self-drying effects, and capillary pressure. The scarcity of free water caused by a low w/b and an excessively high proportion of fine capillaries are the core reasons why its autogenous shrinkage is far greater than that of ordinary concrete. However, there remains controversy regarding the relative contributions of these three factors to the various stages of shrinkage and their quantitative decoupling.
(2) The regulatory effect of SCMS on HPC autogenous shrinkage is primarily governed by their own reactivity and particle characteristics, exhibiting strong coupling with dosage and w/b. Low-reactivity admixtures mainly suppress autogenous shrinkage, with effects that are “strong early on and weak later”. However, there is currently no consensus on whether the “promotion–inhibition” transition dosage threshold for highly active SCMs is universal, or on how w/b affects this transition range. These questions remain to be addressed through systematic and in-depth research.
(3) The use of composite SCMS is the mainstream approach for engineering control of HPC autogenous shrinkage. Binary admixtures achieve a balance between shrinkage and mechanical properties through “early suppression and late enhancement,” while the synergistic effects of ternary and multi-component admixtures better meet the stringent requirements of special engineering applications such as UHPC and marine HPC. However, the mechanism underlying the synergistic effects of multi-component blending systems remains unclear, and there is no unified criterion for evaluating the optimal blending schemes for different application scenarios.
(4) Existing autogenous shrinkage prediction models each have their own advantages and limitations. Although they can preliminarily quantify the influence of SCMs, they fall short in characterizing the synergistic effects of multi-component admixture systems, do not sufficiently account for the coupling of multiple factors, and exhibit poor adaptability to extreme environments. Consequently, there is still significant room for improvement in terms of universality, prediction accuracy, and engineering practicality.
(5) Currently, the HPC field lacks a comprehensive, standardized strength database. Existing mechanical test data are scattered across different test conditions and vary in terms of parameter dimensions, making them difficult to standardize and rendering them unsuitable for direct cross-comparison and model validation. There is an urgent need to establish a comprehensive database that links mix proportions and curing conditions to a full suite of strength indicators, such as compressive strength, splitting tensile strength, and modulus of elasticity. This is the fundamental prerequisite for conducting collaborative research on shrinkage and mechanical properties and for developing data-driven intelligent prediction models.

### 7.2. Outlook

Based on the shortcomings of existing research, this paper suggests that future research could focus on the following areas:

(1) Establish a unified quantitative evaluation system for both traditional and new types of green SCMs, and elucidate the mechanisms linking microstructure and macroscopic shrinkage behavior in single-admixture and composite cementitious systems.
(2) Elucidate the mechanisms of internal synergy in multi-component composite admixtures and establish a universal mix design method applicable to various composite cementitious systems.
(3) Combine multiscale modeling techniques with artificial intelligence algorithms to develop a high-precision autogenous shrinkage prediction model capable of accounting for multi-factor coupling effects and exhibiting greater generalizability.
(4) Develop new low-carbon admixtures and corresponding shrinkage control technologies suitable for extreme service environments such as high temperatures and saline–alkali conditions, thereby promoting the practical application of low-carbon HPC in engineering projects.

## Figures and Tables

**Table 1:** Effect of Fly Ash on HPC autogenous shrinkage.

Type of Fly Ash	W/B	Blend Ratio (%)	Age (Days)	Reduction in Autogenous Shrinkage (%)	References
I	0.33	10, 20, 30	7	19.8, 32.8, 37.1	[125]
I	0.26	15, 30, 45	7	21.3, 41.0, 55.7	[126]
F	0.30	15, 30, 45	3	23.9, 43.5, 58.7	[127]
-	0.32	18, 36, 54	60	13.8, 22.6, 31.8	[128]
F	0.30	30	7, 60	49.5, 23	[73]
F	0.35	25, 50	14	20.1, 45.3	[129]
I	0.34	15, 30, 45	7	16.4, 25.3, 29.8	[130]
I	0.30	15, 30, 45	7	28.6, 41.5, 53.8	[130]
I	0.28	20, 30, 40	28	17.5, 26.4, 32.6	[131]
I	0.24	10, 30, 50	28	12.5, 29.7, 41.3	[132]
I	0.32	40, 50	28	35.6, 29.8	[133]
F	0.35	10	7	57.0	[134]

**Table 2:** Comparison Table of the Characteristics of Different Nanoscale Silicon Dioxide Preparation Processes [178,179].

Preparation Method	Main Ingredients	Particle Size	Product Purity	Temperature	Energy Consumption	Market Share
Fumed	SiCl_4_, H_2_, O_2_	10~50	≥99.9%	1000–1400 °C	Very high	<10%
Precipitation	Na_2_SiO_3_, H_2_SO_4_/HCl	30~100	≥98%	~70 °C	Low	>90% (in China)
Sol-Gel	TEOS/TMOS	20~200	≥99.9%	~80 °C	Low to moderate	Mainly laboratory and small-batch specialty applications
Biogenic Synthesis	Rice husk ash	~276	≥99%	500~800 °C	Moderate	Emerging route, R&D and pilot stages

**Table 3:** Comprehensive comparison of different SCMs [156,186].

Types of SCMs	Resource Availability	Price (RMB/kg)	Autogenous Shrinkage Control Effect	Scalability of Engineering Projects
FA	Industrial solid waste, abundant reserves	0.06~0.19	Inhibition	Mass Concrete, Green and Low-Carbon Projects
			(pronounced in the early stages, diminishing in the later stages)	
BFS	Blast furnace slag from steel mills, stable supply	0.22~0.38	Promotes	High-Strength/Offshore Structures
			(increases as the dosage and fineness increase)	
SF	Byproduct of ferrosilicon production, limited output	0.5~6	Significantly promotes	UHPC
			(particularly noticeable in the early stages)	
LP	Natural mineral deposits	0.22~0.6	A slight increase at low dosages, and suppression at high dosages (due primarily to the dilution effect)	Green, Low-Carbon Concrete
MK	Reserves of high-quality raw materials are limited	0.85~1.6	Promotes growth in the early stages, but may inhibit it later	High-Performance Blended Systems
			(depending on activity and dosage)	
NS	Synthetic nanomaterials, complex manufacturing process	8~15	Extremely significant promotion (substantial increase in early spontaneous contractions)	UHPC or Special Engineering
RHA	Agricultural waste, but calcination conditions must be controlled	0.3~0.6	Inhibition	Suitable for a wide range of applications
			(significant effect, especially at high w/b)	
CC	Abundant clay resources	1.2~1.8	A slight increase at low dosage, inhibition at high dosage	LC3 System, Green HPC
			(strong synergistic effect with LP)	

Note: The price represents bulk engineering purchase prices in China. Actual cost varies with fineness, activity grade, purity, geographical location and transportation cost. Small-batch laboratory samples are generally much more expensive.

**Table 4:** Effect of Different Binary Blended Systems on the autogenous shrinkage of HPC.

Binary Blended Systems	W/B	Age (Days)	Change in Autogenous Shrinkage (%)	Reference
Slag (380 m^2^/kg) 30% and Fly Ash (Grade II) 20%	0.33	7, 28	−26.4, −19.5	[130]
Limestone Powder 15% and Fly Ash (Grade I) 15%	0.34	90	−14.8	[200]
Slag (740 m^2^/kg) 25% and Fly Ash (Grade I) 25%	0.30	7, 28	−35.2, −24.6	[130]
Slag (380 m^2^/kg) 40% and Silica Fume 6%	0.20	7, 28	−8.5, −6.2	[149]
Slag (420 m^2^/kg) 30% and Silica Fume 8%	0.25	7, 28	+4.8, +2.1	[149]
CC 25% and Slag 45%	0.30	7, 28	−17.5, −14.2	[190]
RHA (calcined at 700 °C) 30% and Fly Ash 20%	0.33	7, 28	−62.4, −48.0	[181]
Limestone Powder 20% and CC 10%	0.32	7, 28	+8.7, +6.5	[192]

Note: In the autogenous shrinkage changes, “+” indicates an increase and “−“ indicates a decrease.

**Table 5:** Summary of various shrinkage models.

Models	Core Assumptions	Applicable Systems	Advantages	Limitations
Model based on the modified Tazawa & Miyazawa model [73]	Autogenous shrinkage strain follows a logarithmic relationship with age; the influence of SCMs is characterized by a single mineral composition parameter and a hydration rate coefficient.	Conventional HPC and UHPC systems; systems with pure cement and single SCM dosage	Simple in form, easy to calculate, and highly practical for engineering applications; has been proven to serve as a foundational model for calculating the autogenous shrinkage of UHPC	The predefined functional form is rigid and struggles to capture multicomponent nonlinear interactions; parameter calibration relies on experimental data from specific systems
Model based on the modified GL2000 model [203]	The final shrinkage value is inversely proportional to the square root of compressive strength; shrinkage development correlates with age and surface-to-volume ratio; environmental effects are corrected using a relative humidity function.	Ordinary concrete and fiber-reinforced early-strength concrete; systems with single or binary SCM dosage	An internationally widely used standard for shrinkage prediction; incorporates the cement-type correction factor K to account for material factors	The original model was primarily developed for drying shrinkage and has limited predictive accuracy for the autogenous shrinkage of HPC with low water-to-cement ratios; its applicability to systems with multiple SCMs has not been systematically verified
Equivalent-age correction model based on the Arrhenius equation [205]	The hydration reaction rate is controlled solely by temperature and can be described by the Arrhenius equation; temperature history can be converted into equivalent age at a reference temperature using equivalent time	UHPC precast components; variable-temperature curing conditions (steam curing, summer/winter construction)	Effectively eliminates the temperature-time inequivalence issue and reflects the shrinkage development pattern at the same scale	Significant temporal differences in the hydration activity of different SCMs within multi-component systems render a unified activation energy (Ea) value ineffective; not applicable under isothermal sealed curing conditions
Hyperparameter-tuned ensemble machine learning model [179]	There is a stable nonlinear mapping between autogenous shrinkage, workability, and the rice husk dosage, fineness, water-to-binder ratio, and age; grid/Bayesian overshooting can eliminate overfitting issues in ensemble tree models	RHA-HPC systems; concrete systems incorporating agricultural solid waste SCMs	Strong nonlinear fitting capability and adaptability to multi-factor coupling; ensemble learning outperforms single models	High data dependency and weak physical interpretability; risk of overfitting with small sample sizes
BO-XGBoost machine learning prediction model [206]	Bayesian optimization automatically tunes XGBoost hyperparameters; high-precision predictions are achieved using small sample sizes	Prediction of early-age autogenous shrinkage and drying shrinkage in UHPC	Prediction accuracy (R^2^ ranging from 0.91 to 0.94) and stability outperform BPNN, RF, and SVM	Still requires sufficient training data; applicability is limited to the mix ratio range covered during modeling; extrapolation capability remains to be verified
Machine learning prediction model coupling hydration heat and internal relative humidity [205]	Machine learning is used to predict two key intermediate variables—hydration heat and internal relative humidity—which are then incorporated into a physical constitutive model to calculate autogenous shrinkage	Covers multi-component blended systems; balances physical consistency with data adaptability	Combines physical mechanisms with data-driven approaches, offering both prediction accuracy and physical interpretability; enables multi-objective mix design optimization via NSGA-II	High modeling complexity; the model is still in the development stage, with insufficient engineering validation data

## Data Availability

No new data were created or analyzed in this study. Data sharing is not applicable to this article.
